# Cancer-associated fibroblasts enhance colorectal cancer lymphatic metastasis via CLEC11A/LGR5-mediated WNT pathway activation

**DOI:** 10.1172/JCI194243

**Published:** 2025-10-15

**Authors:** Chuhan Zhang, Teng Pan, Yuyuan Zhang, Yushuai Wu, Anning Zuo, Shutong Liu, Yuhao Ba, Benyu Liu, Shuaixi Yang, Yukang Chen, Hui Xu, Peng Luo, Quan Cheng, Siyuan Weng, Long Liu, Xing Zhou, Jingyuan Ning, Xinwei Han, Jinhai Deng, Zaoqu Liu

**Affiliations:** 1 Department of Interventional Radiology and; 2 Department of Oncology, The First Affiliated Hospital of Zhengzhou University, Zhengzhou, Henan, China.; 3 Interventional Institute of Zhengzhou University, Zhengzhou, Henan, China.; 4 Interventional Treatment and Clinical Research Center of Henan Province, Zhengzhou, Henan, China.; 5 Longgang District Maternity and Child Healthcare Hospital of Shenzhen City (Longgang Maternity and Child Institute of Shantou University Medical College), Shenzhen, Guangdong, China.; 6 Shanghai Academy of Artificial Intelligence for Science, Shanghai, China.; 7 Tianjian Laboratory of Advanced Biomedical Sciences, Academy of Medical Sciences, Zhengzhou University, Zhengzhou, Henan, China.; 8 Department of Colorectal Surgery, The First Affiliated Hospital of Zhengzhou University, Zhengzhou, Henan, China.; 9 Department of Oncology, Zhujiang Hospital, Southern Medical University, Guangzhou, Guangdong, China.; 10 Department of Neurosurgery, Xiangya Hospital, Central South University, Changsha, Hunan, China.; 11 National Cancer Center/National Clinical Research Center for Cancer/Cancer Hospital, Chinese Academy of Medical Sciences and Peking Union Medical College, Beijing, China.; 12 Department of Hepatobiliary Surgery, The First Affiliated Hospital of Xi’an Jiaotong University, Xi’an, Shanxi, China.; 13 Department of Pediatric Surgery, The First Affiliated Hospital of Zhengzhou University, Zhengzhou, Henan, China.; 14 State Key Laboratory of Common Mechanism Research for Major Diseases & Department of Medical Genetics, Institute of Basic Medical Sciences & School of Basic Medicine, Chinese Academy of Medical Sciences & Peking Union Medical College, Beijing, China.; 15 Richard Dimbleby Laboratory of Cancer Research, Comprehensive Cancer Centre, King’s College London, London, United Kingdom.; 16 Institute of Basic Medical Sciences, Chinese Academy of Medical Sciences and Peking Union Medical College, Beijing, China.

**Keywords:** Gastroenterology, Oncology, Bioinformatics, Colorectal cancer, Machine learning

## Abstract

Hypoxia in the tumor microenvironment promotes lymphatic metastasis, yet the role of cancer-associated fibroblasts (CAFs) in this process remains insufficiently elucidated in colorectal cancer (CRC). In this study, we developed a large language model–based cellular hypoxia–predicting classifier to identify hypoxic CAFs (HCAFs) at single-cell resolution. Our findings revealed that HCAFs enhance CRC lymphatic metastasis by secreting CLEC11A, a protein that binds to the *LGR5* receptor on tumor cells, subsequently activating the WNT/β-catenin signaling pathway. This promotes epithelial-mesenchymal transition and lymphangiogenesis, facilitating the spread of tumor cells via the lymphatic system. Furthermore, we demonstrate that the hypoxia-induced transcription factor *HIF1A* regulates the conversion of normoxic CAFs to HCAFs, driving *CLEC11A* expression and promoting metastasis. In vivo and vitro experiments confirmed the pro-metastatic role of CLEC11A in CRC, with its inhibition reducing lymphatic metastasis. This effect was markedly reversed by targeting the *LGR5* receptor on tumor cells or inhibiting the WNT/β-catenin pathway, further elucidating the underlying mechanisms of CLEC11A-driven metastasis. These findings underscore the potential of targeting the CLEC11A-LGR5 axis to prevent lymphatic dissemination in CRC. Our study highlights the role of HCAFs in CRC progression and reveals mechanisms of lymphatic metastasis for intervention.

## Abstract 2

This study identifies hypoxic cancer-associated fibroblasts through a large language model-based cellular hypoxia classifier and reveals their biological mechanisms in promoting lymphatic metastasis of colorectal cancer.

## Graphical abstract

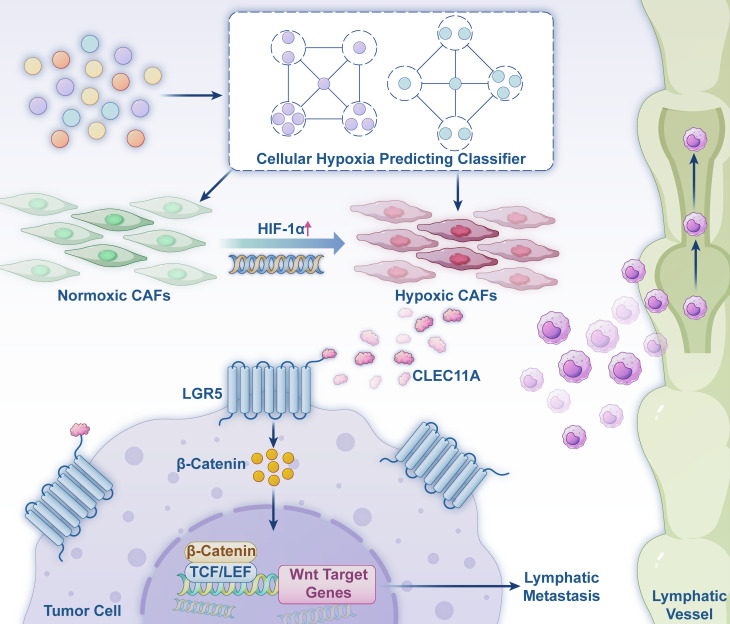

## Introduction

Colorectal cancer (CRC) is the third most prevalent malignancy and the second leading cause of cancer-related deaths worldwide (1, 2). Metastatic dissemination, particularly via the lymphatic system, is a major contributor to CRC mortality. Lymphatic metastasis plays a critical role in the progression of CRC and serves as an independent prognostic factor, strongly associated with poorer survival outcomes (3–5). Additionally, it is a key indicator for assessing tumor invasiveness, guiding clinical staging and surgical planning, informing the administration of postoperative adjuvant chemotherapy, and predicting tumor recurrence (6–10). Despite its well-established clinical importance, the molecular mechanisms underlying lymphatic metastasis in CRC remain poorly understood, highlighting the need for further investigation.

Cancer-associated fibroblasts (CAFs) are a fundamental component of the tumor microenvironment (TME) and play critical roles in diverse biological processes, including tumor angiogenesis, cell proliferation, treatment resistance, and immune escape (11–14). Recently, increasing attention has been paid to the involvement of CAFs in tumor lymphatic metastasis (15). For example, specific CAF subpopulations—such as PDGFRα^+^ITGA11^+^ CAFs in bladder cancer, periostin^+^ CAFs in breast cancer, and FAP^+^ CAFs in esophageal squamous cell carcinoma—have been shown to markedly enhance lymphatic metastasis (16–18). Similarly, in cervical cancer and cholangiocarcinoma, CAFs promote lymphatic metastasis via the secretion of PAI-1 and PDGF-BB, respectively (19, 20). Nevertheless, the mechanisms by which CAFs contribute to lymphatic metastasis in CRC remain to be elucidated.

Recent advancements in single-cell technologies have offered important insights into the functional heterogeneity of CAF subtypes in tumor progression. For example, distinct CAF subtypes have been identified: matrix CAFs (mCAFs) drive extracellular matrix (ECM) remodeling, inflammatory CAFs (iCAFs) secrete pro-inflammatory factors, and antigen presenting CAFs (apCAFs) participate in antigen presentation (21, 22). While the use of cellular markers for CAF identification has greatly advanced our understanding, it offers limited insight into the precise mechanisms by which CAFs contribute to lymphatic metastasis. Furthermore, the TME is inherently dynamic, with various environmental conditions inducing cellular state transitions and remodeling gene expression profiles (23–25). A recent study in pancreatic cancer demonstrated that differential activation of the MAPK pathway leads to substantial variations in the functional states and gene expression profiles of CAFs, even among cells expressing identical markers (26). These findings highlight the limitations of classical CAF classification based solely on static markers. A more nuanced classification may offer valuable biological insights by incorporating the influence of dynamic TME conditions, such as hypoxia, a well-known modulator that promotes lymphangiogenesis and lymphatic metastasis in breast cancer, cervical cancer, and melanoma (27–29). However, in CRC, how hypoxic conditions affect CAF state transitions and their potential association with lymphatic metastasis remain incompletely understood.

In this study, we integrated bulk, single-cell, and spatial transcriptomic data to systematically investigate the mechanisms by which hypoxic CAFs (HCAFs) drive lymphatic metastasis in CRC. We developed a predictive model based on a large language model (LLM) to accurately identify HCAFs at single-cell resolution, addressing the limitations of previous studies that primarily relied on control experiments and lacked reliable hypoxia ground-truth labels (30, 31). Our analyses revealed that HCAFs are spatially adjacent to tumor cells and engage in robust interactions, with their abundance strongly correlating with lymphatic metastasis. Furthermore, comprehensive bioinformatics analyses, in combination with in vivo and in vitro experiments, demonstrated that CLEC11A secreted by HCAFs binds to the *LGR5* receptor on tumor cells, subsequently activating the WNT/β-catenin signaling pathway to promote lymphatic dissemination. Overall, these findings demonstrate the central role of HCAFs in CRC lymphatic metastasis and suggest that targeting the CLEC11A-LGR5 axis may represent a promising therapeutic strategy.

## Results

### Cellular hypoxia–predicting classifier based on the LLM.

Despite the progress in single-cell analysis, several key challenges remain in accurately identifying hypoxic cells. First, existing methods often fail to achieve robust and generalizable performance across different datasets. While some approaches may outperform raw count–based methods on specific classification models, they often lack the ability to consistently deliver superior results across all datasets, raising concerns about potential overfitting and insufficient generalization. Second, the demand for a large amount of training data poses a hurdle. Although high information density could theoretically reduce the need for extensive training data, in practice, obtaining a sufficient volume of labeled data from various datasets is often unfeasible. Finally, the issue of gene mismatching due to differences in sequencing conditions and postsequencing processing further complicates the process. Traditional methods relying solely on raw counts are highly sensitive to gene missingness, rendering them ineffective when certain genes are absent. This lack of flexibility in handling incomplete gene data limits the applicability and transferability of trained models across diverse sequencing datasets (32, 33).

**Figure 1:**
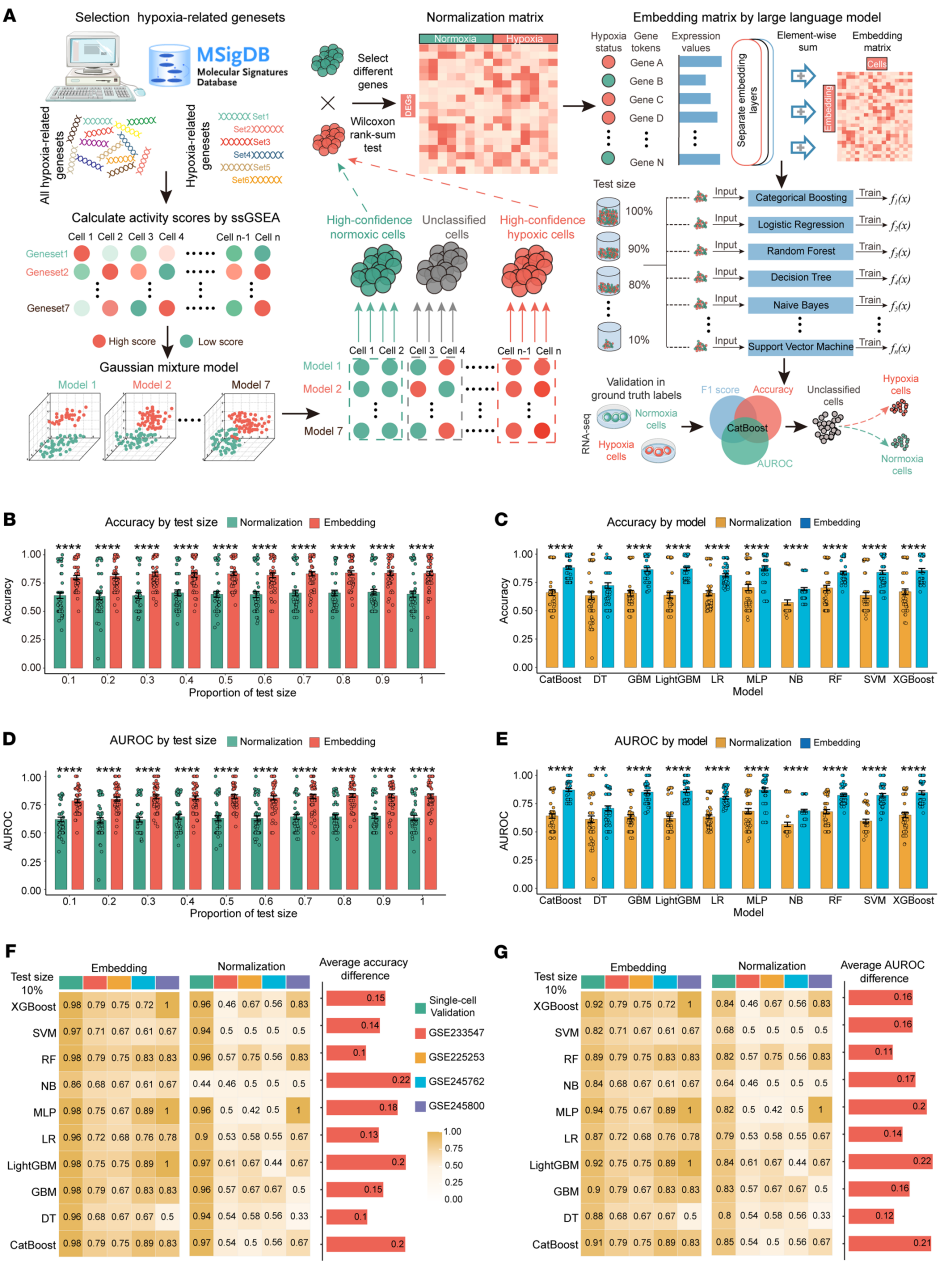
Construction and validation of the CHPC based on the LLM. (**A**) Overview of the CHPC based on the LLM. DEGs, differentially expressed genes; ssGSEA, single-sample gene set enrichment analysis. (**B**–**E**) Differences in accuracy (**B**) and AUROC (**D**) between the 2 matrices across varying test set sizes, as well as accuracy (**C**) and AUROC (**E**) across different machine-learning models. (**F** and **G**) Differences in accuracy (**F**) and AUROC (**G**) between the 2 matrices across various machine-learning models and datasets when using 10% of the training data. XGBoost, eXtreme Gradient Boosting; SVM, Support Vector Machine; RF, Random Forest; NB, Naive Bayes; MLP, Multilayer Perceptron; LR, Logistic Regression; LightGBM, Light Gradient Boosting Machine; GBM, Gradient Boosting Machine; DT, Decision Tree; CatBoost, Categorical Boosting. All data are presented as means ± SEM. *P < 0.05, **P < 0.01, ****P < 0.0001; by distribution type, normally distributed data were analyzed using paired *t* test, whereas non-normally distributed data were examined by Wilcoxon’s signed-rank test (**B**–**E**).

To better characterize cellular hypoxia in CRC, we integrated multiple machine-learning models to develop a cellular hypoxia–predicting classifier (CHPC) that leveraged an LLM-inspired framework (Figure 1A).

First, we analyzed 177,202 cells from 57 untreated CRC samples to characterize cellular hypoxia status. Cells were stratified into high-confidence hypoxic/normoxic populations and low-confidence groups using a Gaussian Mixture Model (GMM) based on activity scores derived from canonical hypoxia pathway signatures (34). Finally, we obtained 4,331 positive and 43,603 negative single-cell samples for further training.

Inspired by the capabilities of foundation models in single-cell representation, particularly their exceptional performance in cross-task transfer and few-shot learning, here we applied a pretrained single-cell model, scGPT (35), to generate cell embeddings. These LLM-derived embeddings serve as reliable feature inputs for constructing machine-learning classifiers, particularly for low-confidence cells.

To evaluate the advantages of our approach, we implemented a 10%–100% equal-interval sampling strategy to partition training and validation sets from both high-confidence single-cell cohorts and 4 independent real-world hypoxic cell line validation datasets (36–38). Through systematic comparisons of 10 machine-learning models, we found that classifiers based on the embedding matrix consistently outperformed traditional methods across all experimental conditions, achieving improvements in accuracy, area under the receiver operating characteristic curve (AUROC), and F1 score (Figure 1, B–E, and Supplemental Figure 1, A–E; supplemental material available online with this article; https://doi.org/10.1172/JCI194243DS1). Notably, even with only 10% training data, the embedding matrix demonstrated its high information density by improving mean accuracy by 0.16 (Figure 1F), AUROC by 0.17, and F1-score by 0.20 (Figure 1G and Supplemental Figure 1F), showcasing its superior generalization capability in low-sample-size scenarios. As the training set increased, performance gains were minimal (Supplemental Figure 2, A and B), further highlighting the efficiency of LLM-generated embeddings in capturing cellular features with limited data. Moreover, the LLM-inspired framework’s ability to tolerate gene missingness was evident, as the embeddings could effectively compensate for missing genes based on gene relationships, unlike raw count–based methods that fail when genes are absent. Ultimately, cross-validation based on the average rankings across all metrics identified the CatBoost model as the top performer among the evaluated classifiers (Supplemental Figure 1, G–J), and it was subsequently selected for hypoxia state classification of low-confidence cells.

### HCAFs exhibit stronger interactions with tumor cells, correlating with poor prognosis and increased lymphangiogenesis.

**Figure 2:**
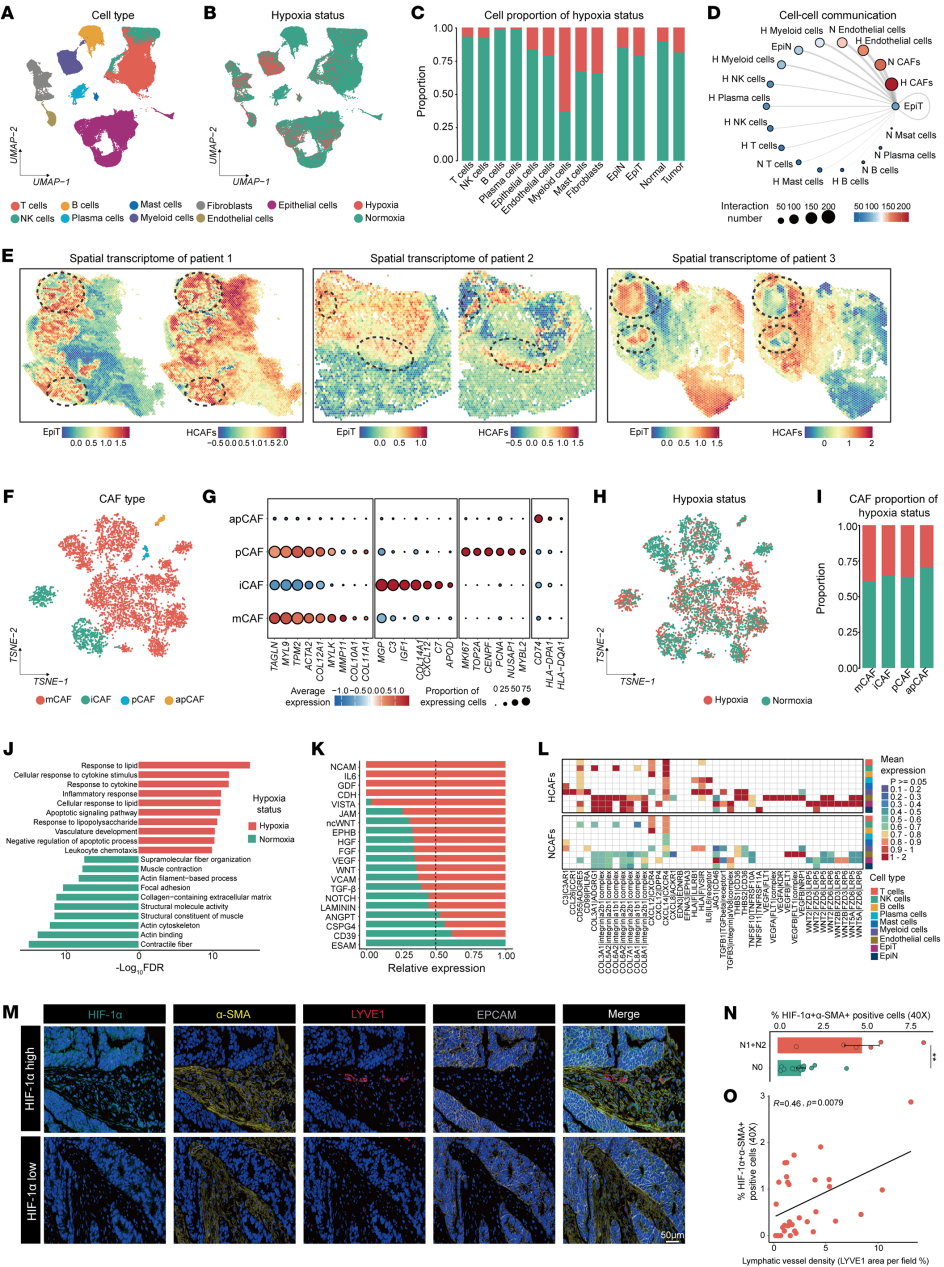
HCAFs interact strongly with tumor cells and are linked to lymphangiogenesis. (**A**) Dimensionality reduction plot showing the distribution of 9 major cell types. (**B**) Dimensionality reduction plot showing the distribution of the hypoxia status. UMAP, uniform manifold approximation and projection. (**C**) Stacked bar charts depicting the distribution of hypoxic and normoxic cells across major cell types, epithelial cells, and different tissues. (**D**) Intercellular communication network between malignant epithelial (EpiT) and other cells. (**E**) Spatial transcriptomics revealing the spatial proximity between HCAFs and EpiT. (**F**) Dimensionality reduction plot depicting the distribution of 4 CAF subtypes, including mCAFs, iCAFs, apCAFs, and pCAFs. (**G**) Dot plot showing the expression of classical CAF-type markers across identified cell populations. (**H**) Dimensionality reduction plot showing the distribution of the hypoxia status in classical CAF types. (**I**) Stacked bar charts depicting the distribution of hypoxic and normoxic cells across 4 CAF types. (**J**) Functional enrichment analysis of HCAFs and NCAFs. (**K**) Bar plots displaying the relative information flow of differential signaling pathways between HCAFs and NCAFs. (**L**) Prediction of ligand–receptor interactions between HCAFs, NCAFs, and other cells. (**M**) mIHC revealing the spatial relationships among HIF-1α, α-SMA, LYVE1, and EPCAM. Scale bar: 50 μm. (**N**) Box plot illustrating the distribution of HCAF proportions across different N stages (*n* = 20). (**O**) Correlation analysis showing a positive correlation between the number of HCAFs and lymphatic vessel density. All data are presented as means ± SEM. **P < 0.01, by Mann-Whitney *U* test (**N**) and Spearman’s rank correlation test (**O**).

Using classical cell markers, we identified 9 distinct cell types in single-cell RNA-Seq (scRNA-Seq**)** data (Figure 2A and Supplemental Figure 3A). The InferCNV algorithm (39) was applied to distinguish malignant epithelial cells (Supplemental Figure 3, B and C). We then applied the classifier to identify cellular hypoxia states, revealing that the proportion of hypoxic cells was more prevalent in myeloid cells, fibroblasts, and mast cells. Additionally, hypoxic cells were enriched in tumor tissues compared with adjacent normal tissue (Figure 2, B and C). To explore cell–cell interactions, we utilized the CellphoneDB algorithm (40), which demonstrated that HCAFs exhibited the highest interaction frequency with malignant epithelial cells (Figure 2D). Additionally, through spatial transcriptomic data and multiplex immunohistochemistry (mIHC) staining using various hypoxic markers (HIF-1α, CA9, and GLUT1), we further confirmed that HCAFs are predominantly enriched in the hypoxic regions of tumor tissues (Supplemental Figure 4, A–C, and Supplemental Figure 5, A–C). Furthermore, the spatial transcriptomics results revealed that HCAFs are in close spatial proximity to malignant epithelial cells (Figure 2E, Supplemental Figure 3D, and Supplemental Figure 6, A–C), suggesting that HCAFs may play a pivotal role in tumor progression. Next, we reclustered CAFs into 4 subgroups: mCAFs, iCAFs, apCAFs, and proliferative CAFs (pCAFs) (Figure 2, F and G) (21, 22, 41). Interestingly, there were no differences in the distribution of these classical CAF subpopulations between hypoxic and normoxic conditions (Figure 2, H and I, and Supplemental Figure 3E), indicating that traditional CAF markers may have limited sensitivity for capturing hypoxia-induced alterations.

Further analysis revealed that HCAFs exhibited biological functions in lipid metabolism, immune response, and angiogenesis, while normoxic CAFs (NCAFs) primarily retained typical fibroblast functions such as collagen contraction (Figure 2J). Cell–cell interaction analyses highlighted increased activity in multiple signaling pathways in HCAFs, including the IL6 pathway (inflammation and immunity), the VEGF pathway (angiogenesis), and pathways involved in cellular proliferation and differentiation (GDF, WNT, TGF-β, and NOTCH), compared with NCAFs (Figure 2K). At the ligand-receptor level, HCAFs showed heightened expression of ligands and receptors involved in WNT and TGF-β pathways with tumor cells, VEGF signaling with endothelial cells, and IL6 signaling with immune cells (Figure 2L). These findings are consistent with previous studies on the roles of HCAFs in angiogenesis, immune responses, ECM modulation, and metabolic reprogramming, further supporting the accuracy of the classifier in identifying hypoxic cells (42–45). Bulk transcriptomic analysis revealed the role of HCAFs in promoting CRC lymphatic metastasis (Supplemental Figure 3F). Then, mIHC and spatial transcriptomic analysis revealed that HCAFs were spatially adjacent not only to tumor cells but also in close proximity to lymphatic endothelial cells (Figure 2M and Supplemental Figure 6). In tissues with lymphatic metastasis, the abundance of HIF-1α^+^α-SMA^+^ cells was also significantly elevated (Figure 2N). The analysis also showed a positive correlation between HIF-1α^+^α-SMA^+^ cells and the extent of lymphatic vessel formation (Figure 2O; *R* = 0.46, *P* = 0.0079), indicating the involvement of HCAFs in lymphangiogenesis.

### HCAF-secreted CLEC11A is linked to unfavorable prognosis and lymphatic metastasis.

**Figure 3:**
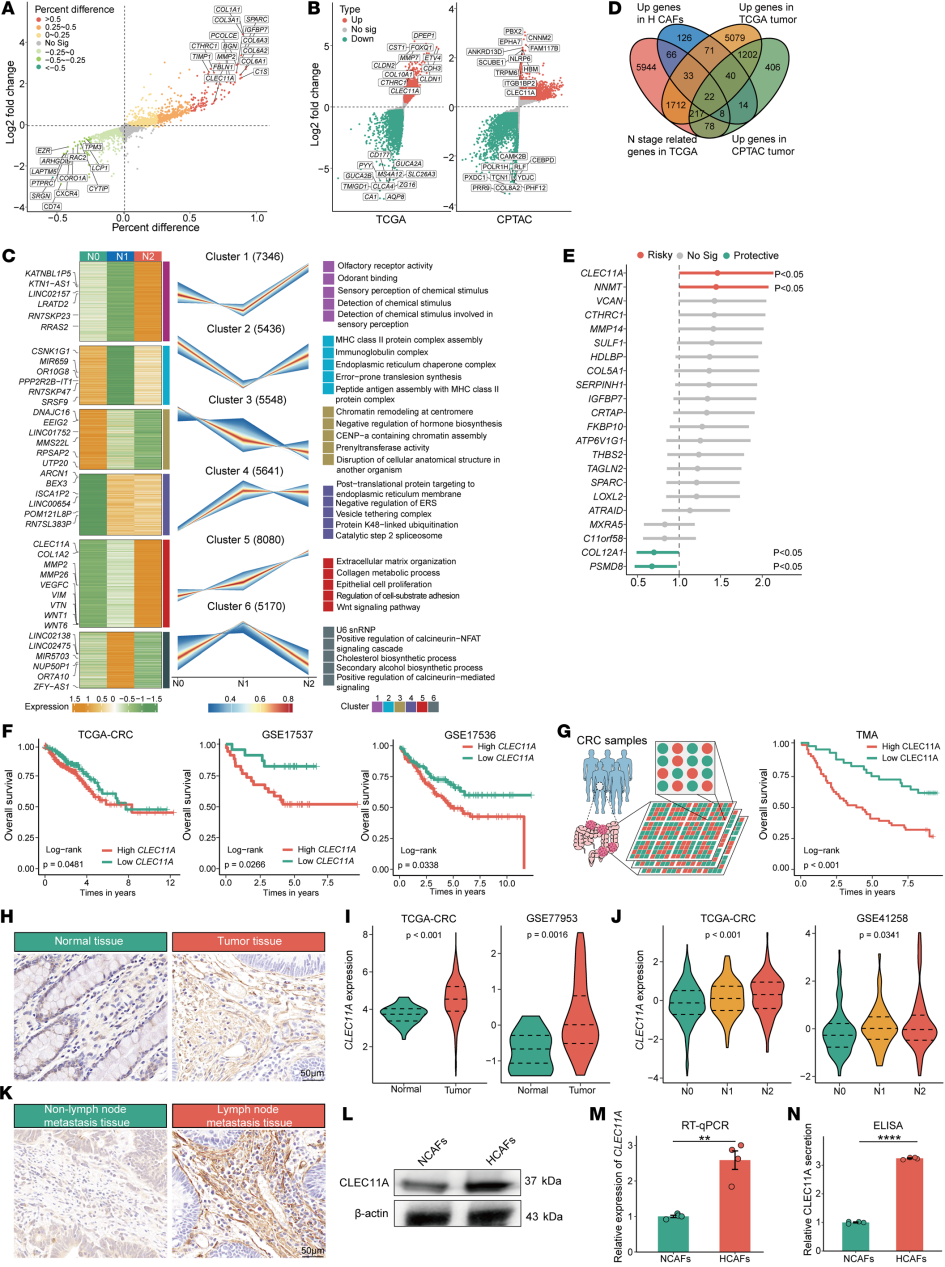
CLEC11A secreted by HCAFs is associated with poor prognosis and lymphatic metastasis. (**A**) Volcano plot displaying differentially expressed genes (DEGs) between HCAFs and NCAFs. (**B**) Volcano plot of DEGs between normal and tumor tissues from TCGA-CRC (left) and CPTAC datasets (right). (**C**) Mfuzz analysis revealing different gene expression patterns dependent on lymph node stages. The left panel shows the gene expression heatmap, the middle shows gene expression curves, and the right shows the results of pathway enrichment analysis. (**D**) Venn diagram of upregulated genes in HCAFs, tumor upregulated genes, and genes in the cluster 5. (**E**) Univariate Cox plot of the 22 shared genes. TMA, tissue microarray cohort. (**F** and **G**) Kaplan-Meier survival curve from independent CRC transcriptome datasets (**F**) and proteomic tissue microarray cohorts (*n* = 90) (**G**), indicating the poorer overall survival in patients with high CLEC11A expression. (**H**) Representative images of IHC staining for CLEC11A in CRC tissues. (**I**) Violin plots displaying *CLEC11A* expression levels in CRC tissues from 2 independent transcriptomes. (**J**) Violin plots displaying *CLEC11A* expression levels in different lymph node stages from 2 independent CRC transcriptomes. (**K**) IHC staining of CLEC11A in CRC tissues with and without lymph node metastasis. (**L**) WB analysis showing the expression levels of CLEC11A in HCAFs and NCAFs. (**M**) RT-qPCR analysis of *CLEC11A* mRNA levels in HCAFs and NCAFs (*n* = 4 per group). (**N**) ELISA quantification of CLEC11A levels in HCAFs and NCAFs (*n* = 4 per group). Scale bars: 50 μm (**H** and **K**). All data are presented as means ± SEM. **P < 0.01, ****P < 0.0001, by empirical Bayes moderated *t* test with Benjamini-Hochberg correction (**A**–**C**), Wald’s test (**E**), log-rank test (**F** and **G**), Student’s *t* test (**M**, **N**, and **I**; GSE77953), Mann-Whitney *U* test (**I**; TCGA-CRC), Kruskal-Wallis test (**J**; GSE41258), and 1-way ANOVA (**J**; TCGA-CRC).

To elucidate how HCAFs promote CRC progression, we first compared the differential expression profiles between NCAFs and HCAFs. This analysis revealed that HCAFs upregulated multiple ECM-related genes (Figure 3A). Consistently, differential analyses of TCGA-CRC transcriptomic and CPTAC proteomic datasets confirmed that ECM-related genes were markedly upregulated in tumor tissues (Figure 3B). Next, we applied the Mfuzz algorithm (46) to examine the transcriptional dynamics of CRC lymphatic metastasis, which identified 6 distinct gene clusters. Notably, expression in cluster 5 increased with advancing node stage (Figure 3C). Functional enrichment analysis indicated that genes within cluster 5 are predominantly involved in ECM remodeling, WNT signaling, cell adhesion and migration, and epithelial proliferation (Figure 3C). By integrating upregulated genes from single-cell, TCGA, and CPTAC data with those in cluster 5, we identified 22 shared genes (Figure 3D). Cox regression analysis indicated that *CLEC11A* exhibited the highest hazard ratio among these shared genes, suggesting a key role in CRC progression and lymphatic metastasis (Figure 3E).

Subsequent analyses across multiple transcriptomic datasets (Figure 3, F and G, and Supplemental Figure 7C) and a proteomic tissue microarray cohort confirmed that high CLEC11A expression was strongly associated with poor prognosis. Multivariate regression analysis demonstrated that elevated CLEC11A expression exhibited the most significant adverse impact on prognosis compared with other clinical parameters (*P* < 0.001; Supplemental Figure 8). Moreover, transcriptomic analysis and IHC showed CLEC11A was significantly upregulated in tumor tissues (Figure 3, H and I, Supplemental Figure 7D, and Supplemental Figure 9) as well as in primary tumors exhibiting lymphatic metastasis (Figure 3, J and K, and Supplemental Figure 9). Finally, experiments using CAFs under hypoxic conditions found a substantial upregulation of CLEC11A at the protein level (Western blot, Figure 3L), at the mRNA level (reverse transcription quantitative PCR [RT-qPCR], Figure 3M), and in its secreted form as detected in the culture supernatant by ELISA (Figure 3N).

### Hypoxia-activated HIF1A in CAFs and transcriptionally upregulated CLEC11A expression.

**Figure 4:**
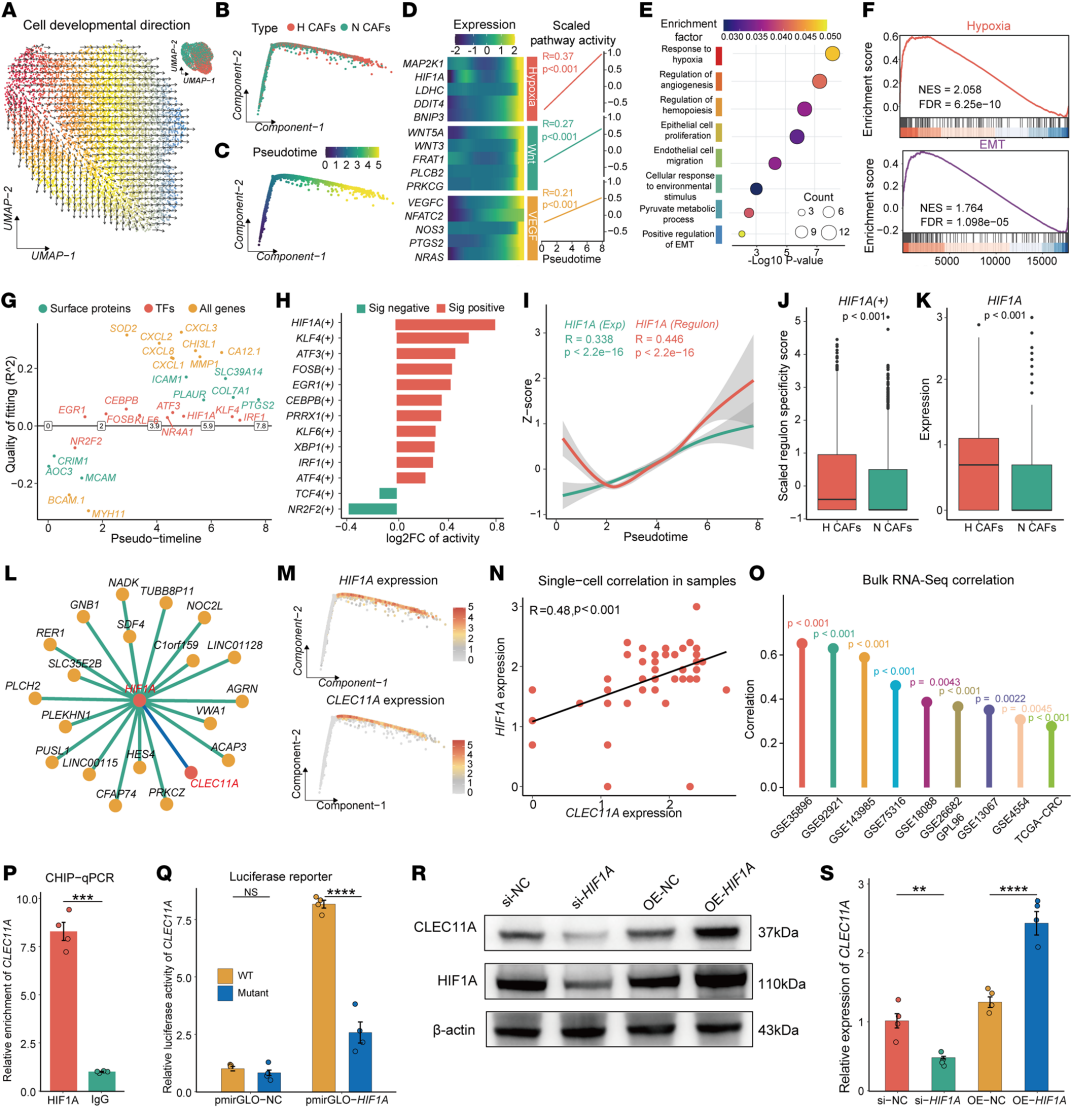
Hypoxia-activated HIF1A in CAFs transcriptionally enhanced the expression of CLEC11A. (**A**–**C**) VECTOR (**A**) and Monocle (**B** and **C**) analyses of the transition from NCAFs to HCAFs. UMAP, uniform manifold approximation and projection. (**D**) Changes in gene expression (left) and pathway activity correlation (right) along Monocle pseudotime. (**E**) Gene Ontology Biological Process enrichment analysis of pseudotime-associated genes. (**F**) Gene set enrichment analysis revealed the association of pseudotime-associated genes related to hypoxia and EMT pathways. NES, normalized enrichment score. (**G**) GeneSwitches analysis identifying key transcription factors involved in the transition from NCAFs to HCAFs. TFs, transcription factors. (**H**) Significant (*P* value < 0.05) differences in transcription factor activity between NCAFs and HCAFs. (**I**) *HIF1A* regulon activity, expression, and pseudotime correlation. (**J** and **K**) Box plot showing differences in *HIF1A* regulatory specificity (**J**) and expression (**K**) between NCAFs and HCAFs. (**L**) The downstream target gene network of *HIF1A*. (**M**) Expression dynamics of *HIF1A* and *CLEC11A* along Monocle pseudotime. (**N** and **O**) Correlation of *HIF1A* and *CLEC11A* in single-cell (**N**) and bulk transcriptomic datasets (**O**). (**P**) ChIP-qPCR analysis showing significant enrichment of HIF1A at the promoter region of *CLEC11A* (*n* = 4 per group). (**Q**) Luciferase assay showing that *HIF1A* enhances WT over mutant CLEC11A promoter activity (*n* = 4 per group). (**R**) WB analysis of CLEC11A and HIF1A protein levels in CAFs. (**S**) RT-qPCR analysis of *CLEC11A* mRNA levels in CAFs (*n* = 4 per group). All data are presented as means ± SEM. **P < 0.01, ***P < 0.001, ****P < 0.0001, by Spearman’s rank correlation test (**D, I**, and **O**), Pearson’s correlation test (**N**), hypergeometric test with Benjamini-Hochberg correction (**E**), permutation test with Benjamini-Hochberg correction (**F**), empirical Bayes moderated *t* test with Benjamini-Hochberg correction (**H**), Mann-Whitney *U* test (**J**, **K**, and **P**), and 1-way ANOVA with Tukey’s post test (**Q** and **S**).

To investigate the transition between NCAFs and HCAFs, we employed both the VECTOR (47) and Monocle (48) algorithms to reconstruct the differentiation trajectory (Figure 4, A–C). Monocle analysis revealed a positive correlation between pseudotime and the activity of hypoxia, WNT, and VEGF pathways, with gene expression gradually increasing along the differentiation trajectory (Figure 4D). Functional enrichment analysis of pseudotime-associated genes indicated notable involvement in hypoxic responses, angiogenesis, epithelial cell proliferation and migration, and epithelial-mesenchymal transition (EMT) (Figure 4, E and F).

To further elucidate the regulatory factors driving the NCAF-to-HCAF transition, we used the GeneSwitches (49) tool, which revealed dynamic changes in the activity of several transcription factors, including *IRF1*, *KLF4*, *ATF3*, *NR4A1*, and *HIF1A* (Figure 4G). Subsequent validation using the single-cell regulatory network inference and clustering (SCENIC) algorithm (50) confirmed that *HIF1A* exhibits strong regulatory activity specifically in HCAFs (Figure 4H). Moreover, correlation analysis showed a positive association between the regulatory activity and expression levels of *HIF1A* and the differentiated pseudotime (Figure 4I). *HIF1A*, a key transcription factor in the hypoxic response (51), exhibited regulatory and expression specificity in HCAFs (31, 52, 53), which was further validated by our findings (Figure 4, J and K).

*HIF1A* has been reported as a transcription factor of *CLEC11A* (Figure 4L) (54). The expression patterns of *CLEC11A* and *HIF1A* during the transition from NCAFs to HCAFs were similar (Figure 4M). Both single-cell and multiple bulk transcriptomic analyses revealed the positive correlation between *HIF1A* and *CLEC11A* (Figure 4, N and O). ChIP-qPCR analysis demonstrated significant enrichment of *HIF1A* at the *CLEC11A* promoter region (Figure 4P), and luciferase reporter assays showed that *HIF1A* markedly enhanced *CLEC11A* promoter activity (Figure 4Q). Furthermore, CAF cell lines overexpressing *HIF1A* (CAF-OE-*HIF1A*), generated via lentiviral transduction (Supplemental Figure 10), exhibited a significant increase in CLEC11A expression at both the protein level (Western blot, Figure 4R) and mRNA level (RT-qPCR, Figure 4S). In contrast, knockdown of *HIF1A* (CAF-si-*HIF1A*) led to a reduction in CLEC11A expression (Figure 4, R and S). These findings indicate that *HIF1A* plays a crucial role in driving the transition from NCAFs to HCAFs and transcriptionally upregulating *CLEC11A*.

### CLEC11A promotes lymphangiogenesis and lymphatic metastasis in vivo.

**Figure 5:**
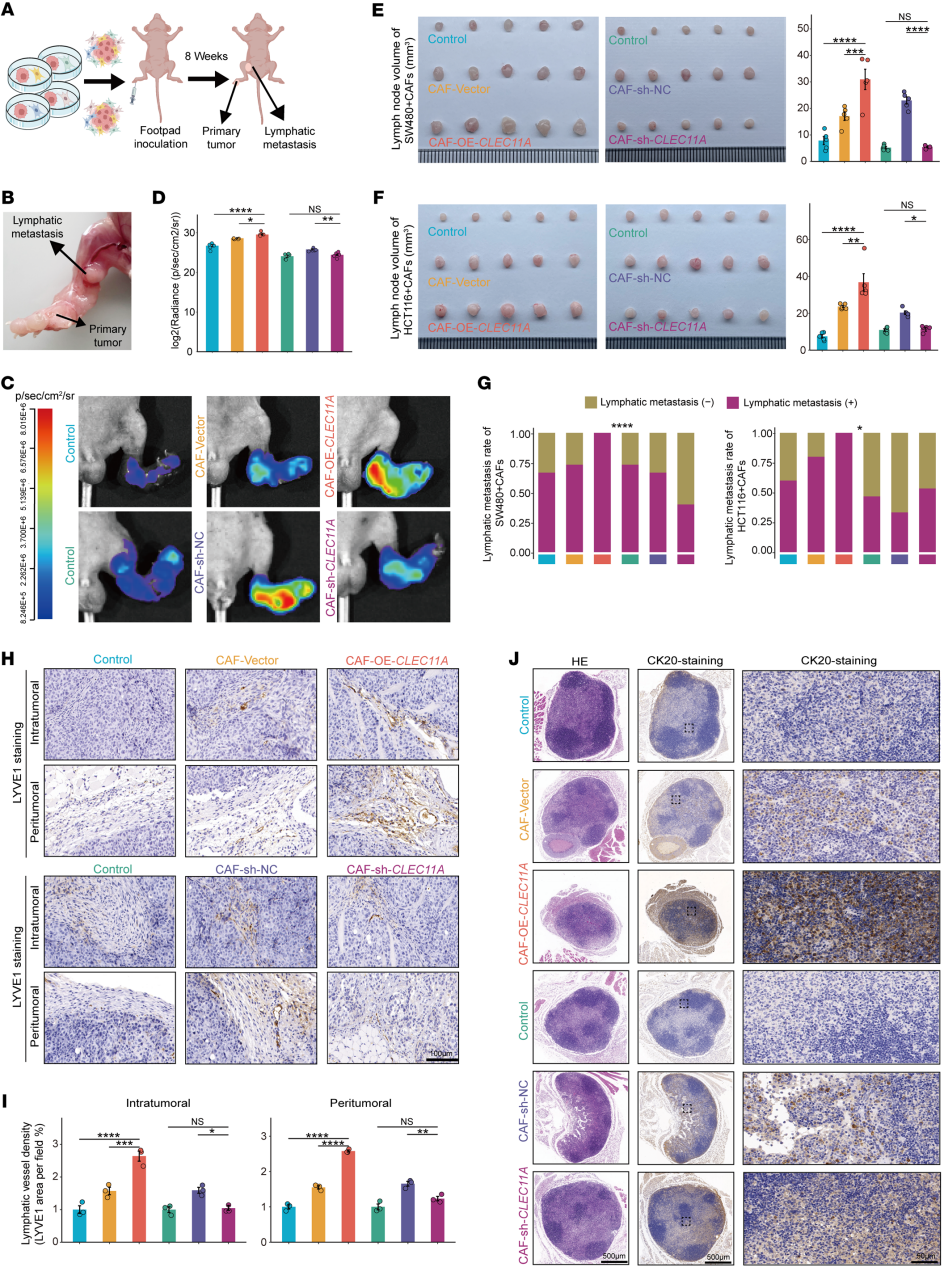
CLEC11A promotes lymphangiogenesis and metastasis in vivo. (**A**) Schematic diagram of popliteal lymph node metastasis model establishment in nude mice. (**B**) Representative images of popliteal lymph node metastasis in a nude mouse model. (**C** and **D**) Representative bioluminescence images (**C**) and bioluminescence quantification (**D**) of popliteal lymph node metastasis in the mouse model (*n* = 5 per group). (**E** and **F**) Representative images of the mouse popliteal lymph node metastasis model generated using specific CAFs and SW480 (**E**) or HCT116 (**F**) cell treatment. Histograms quantifying lymph node volumes (mm³) in nude mice (*n* = 5 per group). (**G**) Lymph node metastasis rates in nude mice inoculated with specific CAFs and SW480 (left) or HCT116 (right) cells (*n* = 15 per group). (**H** and **I**) Representative images (**H**) of anti-LYVE1 staining in plantar tumor tissues. and histogram (**I**) showing the ratio of LYVE1-positive lymphatic vessels (*n* = 3 per group). (**J**) IHC with anti–cytokeratin 20 (CK20) antibody and H&E staining was performed on the CLEC11A overexpression group and CLEC11A knockdown group, showing representative images of the popliteal lymph nodes. Scale bars: 100 μm (**H**), 50 μm (**J**, right), 500 μm (**J**, left and middle). All data are presented as means ± SEM. *P < 0.05, **P < 0.01, ***P < 0.001, ****P < 0.0001, by 1-way ANOVA with Tukey’s post test (**D**–**F** and **I**) and χ^2^ test (**G**).

To investigate the role of CLEC11A in CRC lymphatic metastasis, we established the popliteal lymph node metastasis model in immunodeficient nude mice. In this model, CAFs with stable overexpression (CAF-OE-*CLEC11A*) or knockdown (CAF-sh-*CLEC11A*) of *CLEC11A* were coinjected with SW480 or HCT116 cells into the footpad (Figure 5, A and B). In vivo fluorescence imaging results showed that the lymph node fluorescence intensity in the CAF-OE-*CLEC11A* and HCT116 cell coinjection group was higher than that in the tumor cell–only injection group and the CAF-vector coinjection group. Meanwhile, the fluorescence intensity in the CAF-sh-*CLEC11A* coinjection group was lower than that in the CAF-sh-NC coinjection group (Figure 5, C and D). In both the SW480 and HCT116 models, overexpression of CLEC11A in CAFs significantly increased the lymph node volume and metastasis rate compared with other groups. Conversely, the CAF-sh-*CLEC11A* group exhibited significantly smaller lymph node volumes and a reduced metastasis rate compared with the CAF-sh-NC group, further confirming the role of CLEC11A in promoting CRC lymphatic metastasis (Figure 5, E–G). Additionally, IHC analysis demonstrated that CLEC11A overexpression elevated lymph vessel density in footpad tumors and enhanced cytokeratin 20 expression in popliteal lymph nodes, indicating a higher level of metastatic spread. In contrast, CLEC11A knockdown suppressed these effects (Figure 5, H–J). These findings suggest that CLEC11A contributes to CRC lymphatic metastasis in vivo.

### Tumor cell–dependent CLEC11A promotes lymphatic vessel abnormalities and lymphangiogenesis in vitro.

**Figure 6:**
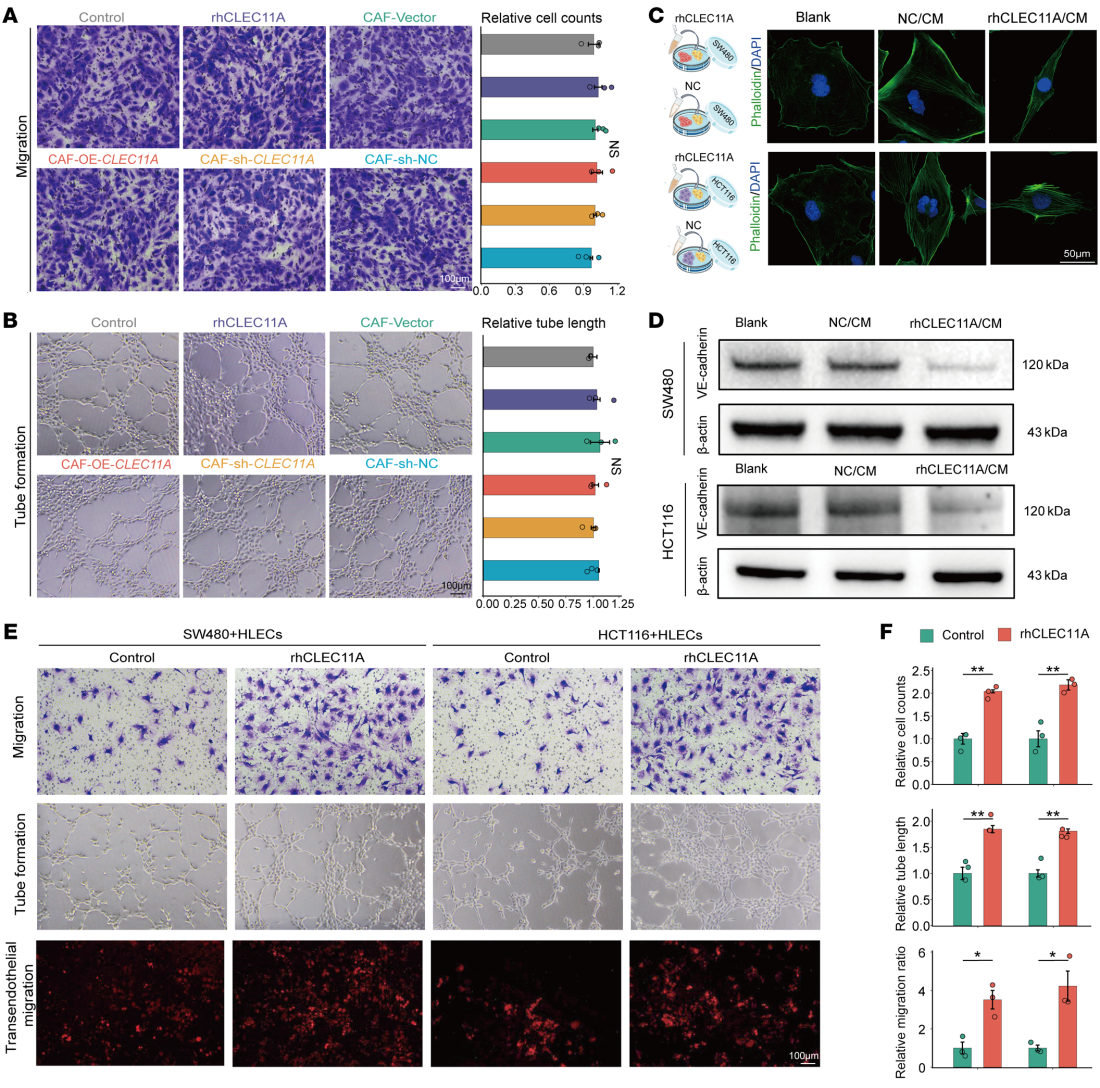
CLEC11A promotes lymphatic vessel abnormalities and lymphangiogenesis in vitro in a tumor cell–dependent manner. (**A** and **B**) Representative images (left) and quantification (right) of HLEC migration (**A**) and tube formation assays (**B**) (*n* = 3 per group). (**C**) Experimental grouping under different conditions and representative phalloidin/DAPI staining images of HLECs. (**D**) WB analysis of VE-cadherin in HLECs cultured in conditioned medium of CRC cell line with different treatments. (**E** and **F**) Representative images (**E**) and quantitative analysis (**F**) of HLEC migration (top), tube formation (middle), and SW480/HCT116 cell transendothelial migration (bottom) in coculture with HLECs using CRC cell line–conditioned media under different treatment conditions (*n* = 3 per group). Scale bars: 100 μm (**A**, **B**, and **E**), 50 μm (**C**). All data are presented as means ± SEM. *P < 0.05, **P < 0.01, by 1-way ANOVA (**A** and **B**) and Student’s *t* test (**F**).

Although CLEC11A promotes lymphangiogenesis and lymph node metastasis in vivo, its direct impact on human lymphatic endothelial cells (HLECs) under in vitro conditions remains unclear. In vitro experiments demonstrated that neither treatment of CAFs with recombinant human CLEC11A (rhCLEC11A) protein nor modulation of *CLEC11A* expression (overexpression/knockdown) in CAFs affected the tube formation or migration capabilities of HLECs (Figure 6, A and B). These findings suggest that, while CLEC11A contributes to lymphangiogenesis and lymph node metastasis in CRC in vivo, its direct effect on HLECs is limited under in vitro conditions. To further investigate whether the role of CLEC11A in promoting lymphangiogenesis and lymph node metastasis in vivo is tumor cell dependent, we used the conditioned medium of rhCLEC11A-treated SW480 cell line to culture HLECs. Phalloidin staining revealed that HLECs in the rhCLEC11A-treated group transitioned from a typical cobblestone morphology to a spindle shape (Figure 6C), suggesting potential alterations in their functional state. Western blot (WB) analysis further revealed that rhCLEC11A treatment reduced VE-cadherin expression in HLECs, indicating weakened intercellular adhesion and enhanced migratory capacity (Figure 6D). Functional assays showed that rhCLEC11A treatment markedly increased HLEC lymphangiogenic and migratory abilities while disrupting lymphatic vessel integrity (Figure 6, E and F). These findings suggest that CLEC11A may mediate HLEC dysfunction through tumor cells, promoting aberrant lymphangiogenesis and tumor lymphatic metastasis.

### CLEC11A targets tumor cells to promote EMT and VEGFC production, leading to lymphangiogenesis and lymphatic metastasis.

**Figure 7:**
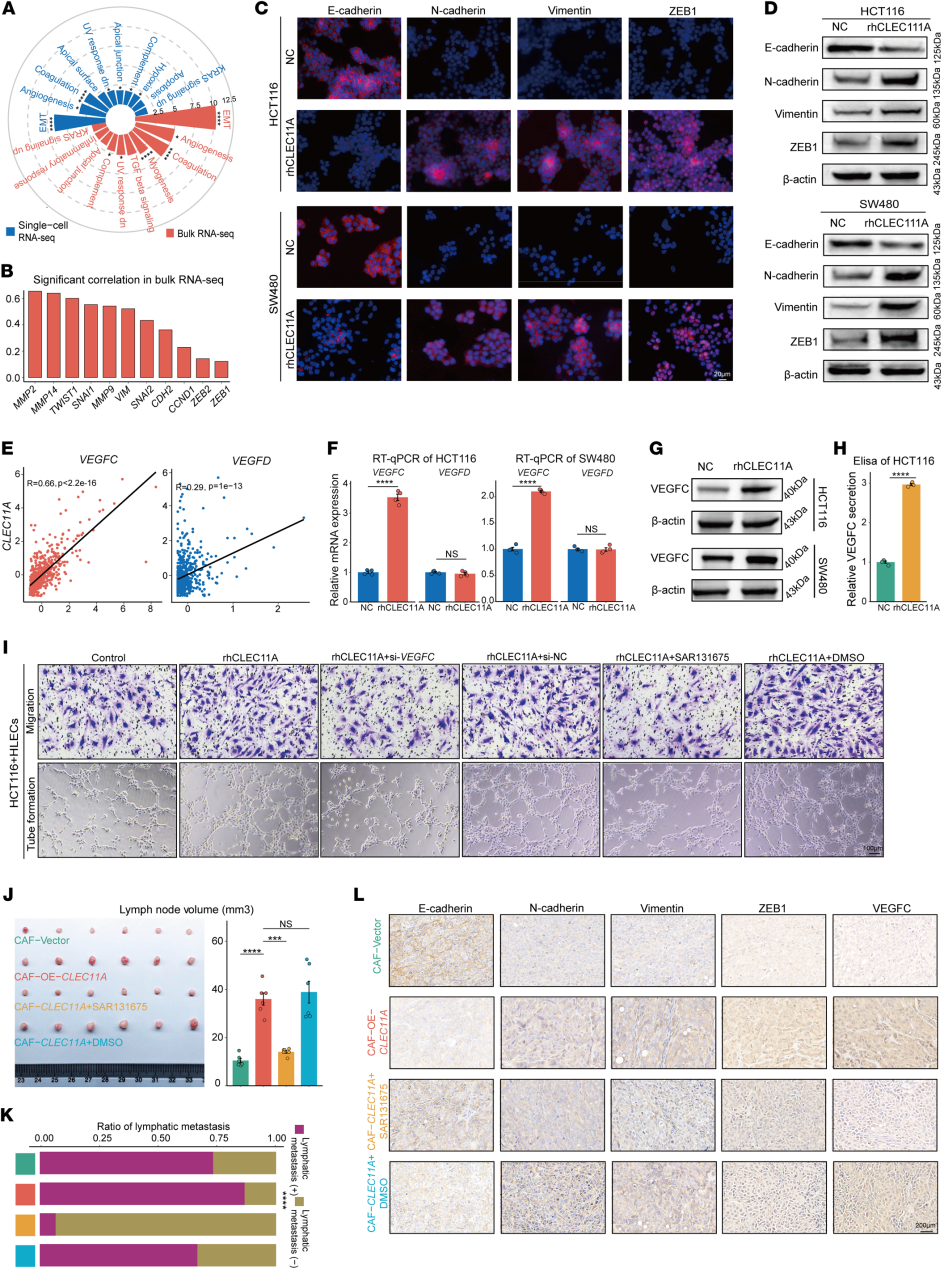
CLEC11A promotes EMT and VEGFC production in tumor cells, leading to lymphangiogenesis and lymphatic metastasis. (**A**) *CLEC11A* showing a strong association with EMT pathway activity in both single-cell and bulk datasets. (**B**) *CLEC11A* expression showing the significant correlation with EMT-related genes (*P* < 0.05) in the TCGA-CRC dataset. (**C** and **D**) Immunofluorescence (**C**) and WB (**D**) analysis of EMT-related genes in CRC cell lines treated with rhCLEC11A. (**E**) Correlation plot showing positive associations between *CLEC11A* and *VEGFC/VEGFD* gene expression in the TCGA-CRC dataset. (**F**) RT-qPCR analysis of *VEGFC* and *VEGFD* expression in SW480 and HCT116 cells treated with rhCLEC11A (*n* = 4 per group). (**G**) WB analysis of VEGFC expression in SW480 and HCT116 cells treated with rhCLEC11A. (**H**) ELISA quantification of VEGFC levels in HCT116 cells treated with rhCLEC11A (*n* = 4 per group). (**I**) Representative images of HLEC migration (top) and tube formation (bottom) assays cultured in conditioned media under specific treatments. (**J**) Representative images of popliteal lymph nodes from the mouse metastasis model established using HCT116 cells coinjected with CAFs subjected to specific treatments. Histograms quantify lymph node volumes (mm³) in nude mice (*n* = 6 per group). (**K**) Ratio of metastasis to total dissected lymph nodes in mice inoculated with specific CAFs and HCT116 cells (*n* = 15 per group). (**L**) IHC staining of E-cadherin, N-cadherin, Vimentin, ZEB1, and VEGFC. Scale bars: 20 μm (**C**), 100 μm (**I**), 200 μm (**L**). All data are presented as means ± SEM. *P < 0.05, ***P < 0.001, ****P < 0.0001, by Spearman’s rank correlation test (**A**, **B**, and **E**), Welch’s t test (**F** and **H**), 1-way ANOVA with Tukey’s post test (**J**), and χ^2^ test (**K**).

To further investigate how CLEC11A promotes lymphangiogenesis and lymphatic metastasis through its effects on tumor cells, we first analyzed the correlation between *CLEC11A* and cancer hallmark pathways (55). Both single-cell and bulk analyses revealed a significant positive correlation between *CLEC11A* expression and the EMT pathway, as well as with EMT-related genes (Figure 7, A and B). Immunofluorescence results showed that treatment with rhCLEC11A enhanced the expression of EMT-related genes in the SW480 and HCT116 cell lines (Figure 7C), which was further supported by WB analysis (Figure 7D).

Previous studies have demonstrated that VEGF family members, particularly VEGFC and VEGFD, are crucial in promoting lymphangiogenesis and lymphatic metastasis in various cancers (56). By analyzing the TCGA-CRC dataset, we identified a significant correlation between *CLEC11A* expression and that of *VEGFC* (*R* = 0.66, *P* < 2.2e-16; Figure 7E) and *VEGFD* (*R* = 0.29, *P* = 1e-13; Figure 7E). Subsequently, RT-qPCR and WB analyses revealed that rhCLEC11A treatment upregulated VEGFC expression in tumor cells, with no significant effect on VEGFD expression (Figure 7, F and G). Further ELISA analysis confirmed a significant increase in VEGFC expression at the protein level (Figure 7H and Supplemental Figure 11).

Next, we investigated whether CLEC11A-induced lymphangiogenesis and lymphatic metastasis depend on VEGFC. In vitro experiments demonstrated that silencing *VEGFC* or using the VEGFR3 inhibitor (SAR131675) to block the VEGFC/VEGFR3 signaling pathway reduced CLEC11A-induced lymphatic vessel migration and formation (Figure 7I and Supplemental Figure 12, A–E). In the popliteal lymph node metastasis animal model, blocking VEGFC/VEGFR3 signaling inhibited the effects of CLEC11A, resulting in smaller lymph nodes and reduced lymphatic metastasis (Figure 7, J and K, and Supplemental Figure 12, F and G). IHC analysis further showed that in the CLEC11A-overexpressing group, VEGFC and EMT-related gene expression levels were elevated. However, after blocking the VEGFC/VEGFR3 signaling pathway, their expression levels decreased (Figure 7L). In conclusion, these in vitro and in vivo findings indicate that CLEC11A promotes lymphangiogenesis and lymphatic metastasis by enhancing the EMT process in tumor cells and upregulating VEGFC expression.

### CLEC11A binds to the LGR5 receptor on tumor cells to promote lymphangiogenesis and lymphatic metastasis.

**Figure 8:**
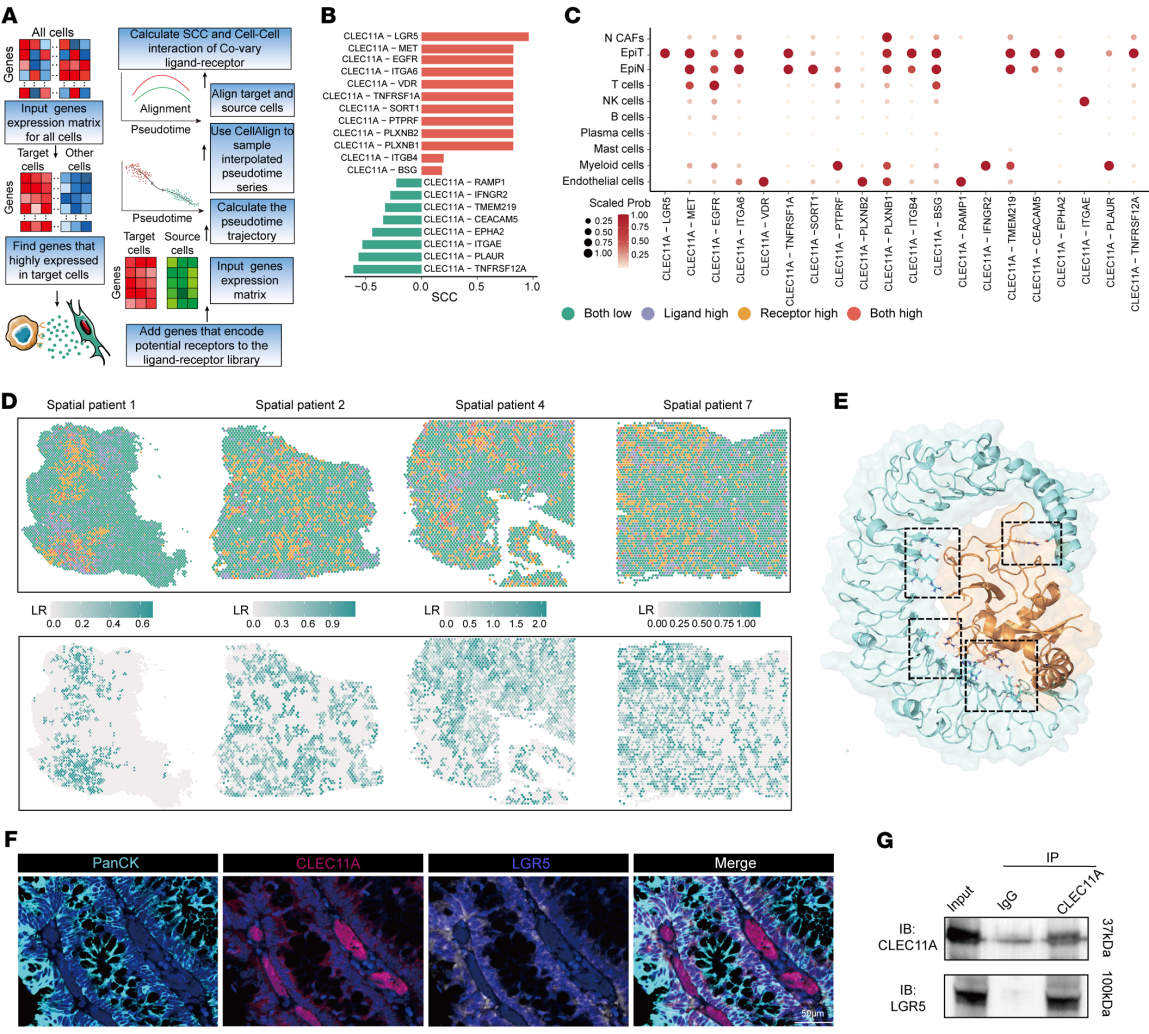
CLEC11A promotes lymphangiogenesis and lymphatic metastasis through its interaction with LGR5 on tumor cells. (**A**) Identification of ligand-receptor pairs and a schematic of the TimeCCI pipeline, illustrating the calculation of Spearman’s correlation coefficients (SCC) for covarying ligand-receptor pairs between HCAFs and tumor cells. (**B**) CLEC11A-LGR5 is the top ligand-receptor pair, with the highest SCC among CLEC11A interactions. (**C**) Normalized interaction probabilities between CLEC11A and its receptors across different cell types. (**D**) ST data showing CLEC11A-LGR5 interactions. (**E**) Molecular dynamics simulation of the CLEC11A-LGR5 complex, with structural visualization of key interacting residues. (**F**) mIHC revealing spatial colocalization between CLEC11A and LGR5. Scale bar: 50 μm. (**G**) Co-IP confirmed the physical interaction between CLEC11A and LGR5 in SW480 cells.

To investigate how CLEC11A secreted by HCAFs acts on tumor cells, we utilized the TimeCCI tool based on our previous study (57) to examine the temporal correlation of potential ligand–receptor interactions in cell–cell communications (Figure 8A). The analysis revealed that the CLEC11A–LGR5 interaction exhibited the highest Spearman’s correlation coefficient within the CLEC11A signaling (Figure 8B), suggesting a strong and specific interaction exclusively between HCAFs and tumor cells (Figure 8C). Spatial transcriptomic (ST) data further validated the extensive CLEC11A–LGR5 interactions within the TME (Figure 8D and Supplemental Figure 13A).

To evaluate the stability and binding affinity of the CLEC11A–LGR5 interaction, we conducted molecular dynamics simulations. These simulations indicated that CLEC11A and LGR5 interact through hydrogen bonds, contributing to the stability of the complex (Figure 8E). Root mean square deviation analysis indicated that the complex reached a stable state early in the simulation, and additional analyses of the radius of gyration and buried surface area confirmed a compact and stable interaction interface (Supplemental Figure 13B). These results suggest that the CLEC11A-LGR5 complex exhibited strong binding affinity and structural integrity. Using mIHC, we observed the spatial proximity between LGR5^+^ tumor cells and CLEC11A^+^ CAFs (Figure 8F). Furthermore, co-IP assays detected the specific interaction between CLEC11A and LGR5 (Figure 8G). Together, these results indicate that CLEC11A binds to LGR5 on tumor cells, providing insights into its role in promoting lymphangiogenesis and lymphatic metastasis.

### CLEC11A activates the WNT/β-catenin pathway via LGR5 on tumor cells to promote EMT and VEGFC secretion.

**Figure 9:**
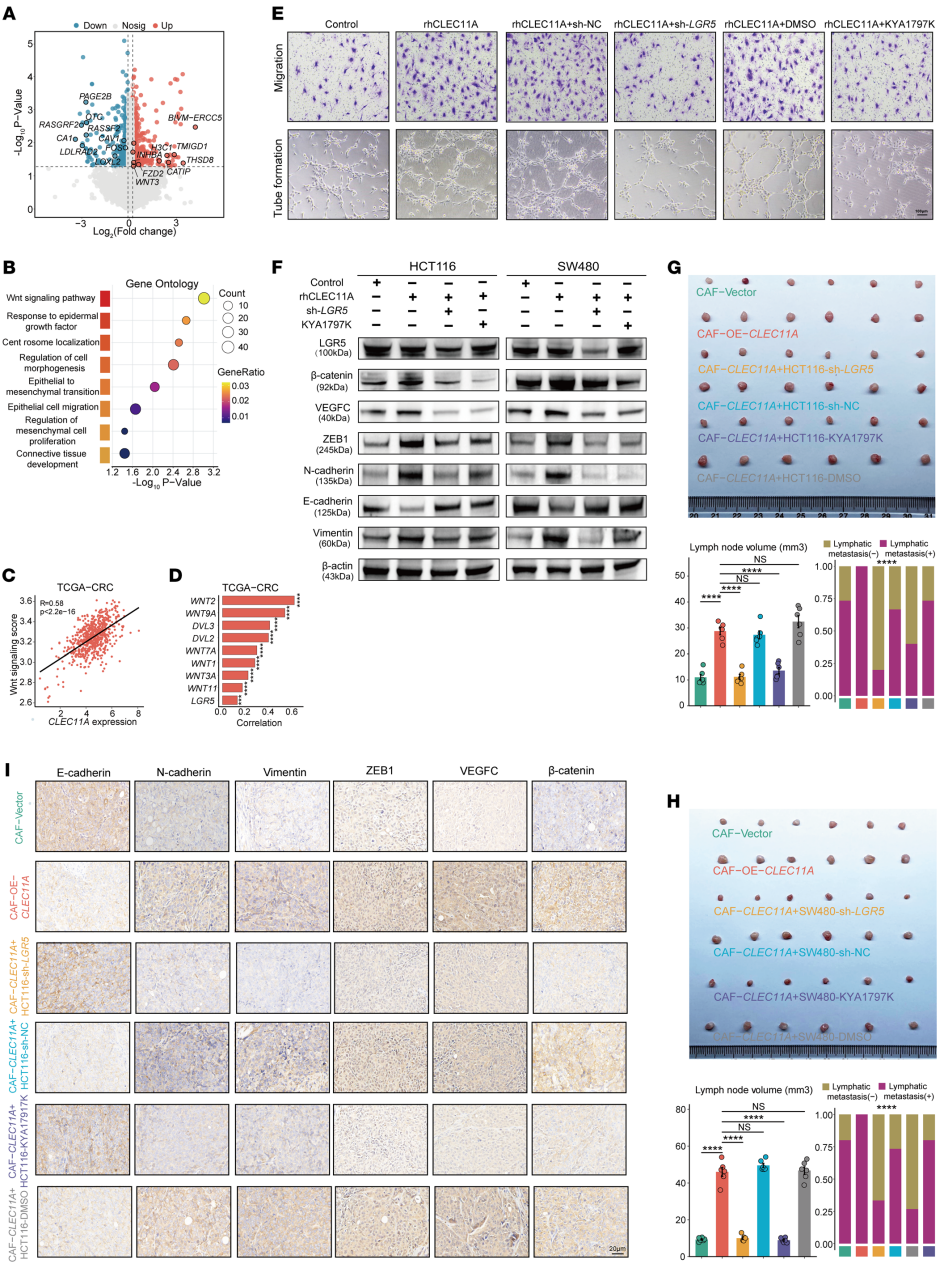
CLEC11A promotes EMT and VEGFC secretion of tumor cells through the interaction with LGR5 to activate the WNT/β-catenin pathway. (**A**) Volcano plot displaying differentially expressed genes (DEGs) between SW480 CRC cells treated with either PBS or rhCLEC11A. (**B**) Gene Ontology enrichment analysis of DEGs highlighting significant enrichment in the WNT signaling pathway. (**C**) Correlation analysis showing a positive association between *CLEC11A* expression and WNT pathway scores in the TCGA-CRC cohort. (**D**) Bar plots depicting correlations between *CLEC11A* expression and WNT-related genes in the TCGA-CRC cohort. (**E**) Representative images of HLEC migration (top) and tube formation (bottom) after culture with HCT116 cell line–conditioned media under different treatments. (**F**) WB analysis of LGR5, β-catenin, VEGFC, ZEB1, N-cadherin, E-cadherin, and Vimentin protein expression in HCT116 or SW480 cells treated with rhCLEC11A, sh-*LGR5*, or KYA1797K. (**G**) Representative images (top) and quantification (bottom left) (*n* = 6 per group) of popliteal metastatic lymph node volume in mice models generated using HCT116 cells and CAFs subjected to specific treatments. Metastasis rates and the ratio of metastatic to total dissected lymph nodes are shown (bottom right) (*n* = 15 per group). (**H**) Representative images (top) and quantification (bottom left) (*n* = 6 per group) of popliteal metastatic lymph node volume in mice models generated using SW480 cells and CAFs subjected to specific treatments. Metastasis rates and the ratio of metastatic to total dissected lymph nodes are shown (bottom right) (*n* = 15 per group). (**I**) IHC staining for protein expression of E-cadherin, N-cadherin, Vimentin, ZEB1, VEGFC, and β-catenin. Scale bars: 100 μm (**E**), 20 μm (**I**). All data are presented as means ± SEM. ***P < 0.001, ****P < 0.0001, by empirical Bayes moderated *t* test with Benjamini-Hochberg correction (**A**), hypergeometric test with Benjamini-Hochberg correction (**B**), Spearman’s rank correlation test (**C** and **D**), 1-way ANOVA with Tukey’s post test (**G** and **H**, bottom left), and χ^2^ test (**G** and **H**, bottom right).

To investigate the mechanism underlying the interaction between CLEC11A and LGR5 in promoting EMT and VEGFC expression in tumor cells, we performed transcriptomic sequencing on SW480 CRC cells treated with PBS or rhCLEC11A. The results revealed that ECM- and WNT pathway–related genes were upregulated in rhCLEC11A-treated cells (Figure 9A). Enrichment analysis revealed that the WNT signaling pathway was enriched in rhCLEC11A-treated cells (Figure 9B). Meanwhile, gene set variation analysis using the TCGA-CRC dataset showed a positive correlation between *CLEC11A* expression and WNT pathway activity, along with upregulation of WNT-related genes (Figure 9, C and D).

In vitro experiments demonstrated that inhibition of LGR5 or treatment with the WNT/β-catenin inhibitor (KYA1797K) suppressed the migratory and tube formation abilities of HLECs (Figure 9E and Supplemental Figure 14, A–F). WB analysis further demonstrated that LGR5 inhibition or KYA1797K treatment reversed the rhCLEC11A-induced upregulation of β-catenin, VEGFC, N-cadherin, ZEB1, and Vimentin expression in tumor cells, while restoring E-cadherin expression levels (Figure 9F). In the lymph node metastasis model, both the LGR5 knockdown group and the KYA1797K treatment group exhibited reduced lymph node volume and lower incidence of lymphatic metastasis (Figure 9, G and H). IHC analysis further showed that the LGR5 knockdown group and KYA1797K treatment group exhibited reduced expression of β-catenin, VEGFC, N-cadherin, ZEB1, and Vimentin, along with increased E-cadherin expression, compared with the control and CLEC11A overexpression groups (Figure 9I).

Overall, these findings suggested that CLEC11A promotes lymphatic metastasis in CRC by activating the WNT/β-catenin pathway via LGR5, thereby enhancing EMT and VEGFC secretion.

## Discussion

In this study, we developed a CHPC based on an LLM to identify HCAFs in CRC and explore their role in lymphatic metastasis. By leveraging this approach, we found that HCAFs promote CRC lymphatic metastasis through the secretion of CLEC11A, which interacts with the *LGR5* receptor on tumor cells to activate the WNT/β-catenin pathway. These findings illustrate the intricate interplay between the TME, cellular states, and metastasis, providing potential insights into CRC progression and therapeutic targets.

Although traditional machine-learning methods such as Support Vector Machines and Random Forests have been applied in scRNA-Seq data analysis, particularly for immune and neural cell type classification (58, 59), challenges including data sparsity, high noise, zero inflation, and gene dropout often result in unstable model performance and limited applicability (32). In contrast, LLMs leveraging pretraining and transfer learning can compensate for data scarcity through prior knowledge integration and extract meaningful signals from noisy data (60). The CHPC model proposed in this study can accurately identify cellular hypoxic states without requiring extensive labeled data, demonstrating strong robustness across multiple platforms and multiscale datasets, thereby providing a more efficient and stable solution for hypoxia state identification.

Recent research indicated that hypoxia promotes tumor lymphatic metastasis by altering various cellular states (27–29), yet the specific role of CAFs in this process remains largely unexplored. This study demonstrates that HCAFs can enhance CRC lymphatic metastasis by secreting CLEC11A. While hypoxia-driven conversion of CAFs to inflammatory phenotypes has been a major focus in cancer research, which involves immune-inflammatory factor secretion to modulate the inflammatory TME (30, 31, 61, 62), our findings describe the role of CAFs in promoting lymphatic dissemination. By identifying CLEC11A as a key secreted factor that interacts with LGR5 on tumor cells, we describe a mechanism through which CAFs influence tumor cell behavior and metastatic potential.

CLEC11A, a secreted protein originally recognized for its role in hematopoietic progenitor cell growth and bone remodeling (63, 64), has recently gained attention in cancer research due to its prognostic value in lung and gastric cancers (65, 66). However, its role in CRC has not been well characterized. This study identified a strong association between CLEC11A secreted by HCAFs and poor prognosis in patients with CRC. Given its secretion properties, CLEC11A may serve as a viable marker for liquid biopsy, facilitating early diagnosis and risk stratification in CRC. Moreover, its marked correlation with N stage suggests potential utility in predicting lymph node metastasis and informing personalized therapeutic strategies.

The WNT/β-catenin pathway plays an important role in CRC initiation and progression, where its aberrant activation drives tumor cell proliferation, invasion, metastasis, and angiogenesis (67). Currently, several pathway inhibitors (including LGK974, PRI-724, and Foxy-5) have entered early-phase clinical trials, though therapeutic development remains in its infancy (68). Our study demonstrates that targeting the *LGR5* receptor on CRC cells effectively inhibits CLEC11A-mediated WNT/β-catenin activation and lymphatic metastasis, thereby expanding potential intervention strategies for this pathway. Furthermore, combining this targeting strategy with existing therapies may suppress chemotherapy-induced compensatory activation of the WNT pathway (69), thereby enhancing conventional treatment efficacy and providing both theoretical foundations and practical approaches for comprehensive CRC therapy.

Another important aspect of our findings is the role of the hypoxia-induced transcription factor *HIF1A* in regulating the conversion of NCAFs to HCAFs. As a regulator of the cellular response to low oxygen levels, *HIF1A* has been shown to control gene expressions involved in angiogenesis, metabolism, and cell survival (51). Our study further reveals that *HIF1A* not only regulates the expression of CLEC11A but also drives the differentiation of NCAFs into HCAFs, contributing to CRC progression. These findings provide information about the molecular mechanisms governing CAF activation in the hypoxic TME and suggest that targeting *HIF1A* could be an effective strategy for preventing CAF-mediated metastasis.

Despite the promising results, there are several limitations to our study that warrant consideration. First, although CHPC demonstrates high accuracy in hypoxic cell recognition, its reliance on large-scale pretrained data may limit its generalizability in specific biological contexts. Furthermore, LLMs consume substantial computational resources when processing high-dimensional single-cell data, which may limit their applicability in certain settings. Mechanistically, we identified HIF1A as a driver of NCAF-to-HCAF transition, but the involvement of other transcription factors cannot be excluded. Furthermore, our analysis was focused on CRC, and further research is needed to validate the role of CLEC11A and HCAFs in lymphatic metastasis across other cancer types. Given the heterogeneity of the TME in different cancers, it will be important to explore whether similar mechanisms are at play in other cancers, such as breast, lung, or gastric cancers. Moreover, the broader biological functions of CLEC11A within the TME, such as its potential effects on immune cell infiltration, endothelial function, and ECM remodeling, require further investigation. The possibility that other signaling pathways may collaborate with the WNT/β-catenin pathway to mediate the metastasis-promoting function of CLEC11A cannot be excluded and warrants further investigation. Finally, while our study establishes CLEC11A as a mediator of lymphatic metastasis, the clinical application of targeting this pathway needs to be explored further. In particular, the development of specific inhibitors or monoclonal antibodies that can block CLEC11A–LGR5 interactions or inhibit the WNT/β-catenin signaling pathway could provide a promising strategy for treating CRC patients with high metastatic potential. Further preclinical and clinical studies will be necessary to evaluate the efficacy and safety of such therapeutic interventions.

In conclusion, our study provides evidence for the role of HCAFs in promoting CRC lymphatic metastasis via the CLEC11A–LGR5 interaction and the activation of the WNT/β-catenin pathway. The identification of *HIF1A* as a key regulator of HCAF differentiation in the hypoxic TME adds another layer of complexity to our understanding of CAF biology. Targeting the CLEC11A-LGR5 axis and *HIF1A* offers promising strategies for inhibiting CRC metastasis. These findings warrant future research aimed at translating these insights into clinical applications, potentially improving the prognosis and treatment outcomes for CRC patients.

## Methods

### Sex as a biological variable.

Sex was not considered as a biological variable in this study. Patient samples and mice of both sexes were used.

### Mouse popliteal lymphatic metastasis model.

Ethical approval for all procedures in this study was granted by Zhengzhou University’s Animal Care and Use Committee. Both male and female BALB/c nude mice, aged 4–6 weeks, were obtained from Vital River Laboratory Animal Technology. Lentivirally transduced CRC cells (5 × 10^6^ per mouse) were mixed with lentivirally transduced CAF cells (5 × 10^6^ per mouse) and injected into the footpads of the mice. After 8 weeks, the research team euthanized the mice and excised the footpad tumors and popliteal lymph nodes. Lymph node volumes were measured, and the tissues were subsequently fixed in formalin and paraffin embedded.

### Cell culture and treatments.

The human CRC cell lines SW480 and HCT116 (Pricella) were cultured in DMEM supplemented with 10% FBS (Biochannel) and 1% penicillin-streptomycin (Servicebio). CAF-vector, CAF-OE-*CLEC11A*, CAF-sh-*CLEC11A*, and CAF-sh-NC cells were cultured under the same conditions. HLECs (Fuhengbio) were cultured in HLEC-specific medium (Fuhengbio). All cells were incubated at 37°C with 5% CO_2_ for 24 hours. After the initial culture, the medium for CRC cells was replaced with complete medium containing 200 ng/mL rhCLEC11A. Subsequent experiments followed the study protocol.

### Lentiviral construction and stable cell line generation.

The human sh-*CLEC11A* sequence was introduced into the lentiviral vector pLKO.1-*EGFP-Puro*, and lentiviral particles were generated. For control purposes, the empty vector (sh-NC) was used. Cells were transduced with these lentiviral particles and subjected to puromycin selection for 14 days to establish stable CLEC11A knockdown cell lines (CAF-sh-*CLEC11A*). These stable knockdown cell lines were cultured up to passage 20 for experimental use. The same methodology was applied to generate control cell lines (sh-NC). Additionally, the full-length human *CLEC11A* gene was subcloned into the lentiviral vector pLent-*EF1a-FH-CMV-RFP-Puro* and packaged into pLent-*CLEC11A* lentiviral particles. An empty vector (pLent-empty) was used as a control. Cells were infected with either pLent-*CLEC11A* or pLent-empty lentiviral particles, followed by puromycin selection for 14 days, leading to the creation of stable CLEC11A-overexpressing CAF cell lines (CAF-OE-*CLEC11A*). These stable CAF-*CLEC11A* cell lines were used for experimental purposes alongside the control (CAF-NC) cells, with all cells maintained up to passage 20.

### Primary human CAF isolation.

Fresh CRC tissues were obtained from patients at The First Affiliated Hospital of Zhengzhou University, with ethical approval granted by the Zhengzhou University Ethics Committee. The tissues were washed multiple times with 5× PBS containing trypsin until the PBS was clear, with fat and necrotic material removed. The tissues were then minced into 1 mm³ sections and treated with 1 mg/mL type IV collagenase (Thermo Fisher Scientific) at 37°C for 2 hours. After centrifugation and filtration through a 200-mesh filter to remove the supernatant, the tissue fragments were resuspended in DMEM (high glucose) containing 10% FBS and seeded into 6 cm culture dishes. After 72 hours, the culture medium was changed to discard nonadherent cells.

### Generation of HCAFs.

Normal CAFs were cultured under normoxic conditions (21% O_2_, 5% CO_2_, 37°C) until 70%–80% confluence. For hypoxic induction, CAFs were incubated in a CO_2_ tri-gas incubator (Thermo Fisher Scientific) set to 1% O_2_, 5% CO_2_, and balanced N_2_ for 48 hours to generate HCAFs. NCAFs (21% O_2_) served as controls. The hypoxic response was verified using reverse transcription qPCR to analyze HIF-1α and VEGF expression.

### Collection and analysis of scRNA-Seq data.

In this study, we collected and analyzed 4 scRNA-Seq datasets (GSE132465, GSE144735, GSE166555, and GSE200997) from the Gene Expression Omnibus database. These datasets comprised 177,202 cells obtained from samples of 57 CRC patients. The R package Seurat was used for scRNA-Seq data preprocessing (70), and DoubletFinder was employed to identify and remove potential doublets (71). Cells with fewer than 500 detected genes, over 20% mitochondrial content, and high dropout genes were removed to prevent analysis interference. The SCTransform method was employed to normalize and scale the scRNA-Seq data. A principal component analysis matrix with 30 components was performed to achieve dimensionality reduction. The Harmony algorithm was applied for batch correction prior to clustering analysis to remove batch effects (72). Using the Seurat functions FindNeighbors and FindClusters, we identified distinct cell clusters. These clusters were subsequently visualized through the t-distributed stochastic neighbor embedding technique. Markers previously identified in published literature were utilized to distinguish each cluster during the initial phase of annotations: T cells (*CD2*, *CD3D*, *CD3E*, *TRBC1*, *CD8A*, and *CD8B*), NK cells (*PRF1*, *KLRF1*, *KLRD1*, *FGFBP2*, and *NKG7*), B cells (*CD19*, *CD79A*, *CD79B*, and *MS4A1*), plasma cells (*TNFRSF17*, *MZB1*, *IGHG1*, and *IGHA1*), myeloid cells (*CD14*, *CD68*, *CD163*, *LYZ*, *S100A8*, and *FCGR3A*), mast cells *(TPSAB1*, *TPSB2*, and *MS4A2*), fibroblasts (*COL1A1*, *COL1A2*, *COL3A1*, *DCN*, *MYH11*, and *ACTA2*), epithelial cells (*EPCAM*, *CD24*, *KRT18*, *KRT8*, and *CEACAM5*), and endothelial cells (*VWF*, *PECAM1*, *CDH5*, *ENG*, *CLDN5*, and *ACKR1*). Large-scale chromosomal copy-number variations were inferred from single-cell transcriptome profiles using the InferCNV R package to distinguish malignant from nonmalignant epithelial cells (39).

### CHPC based on the LLM.

To identify hypoxic cells from single-cell data, we designed a workflow as follows.

Selection of hypoxia-related pathways: We screened hypoxia-associated pathways from the Molecular Signatures Database based on the following criteria: (a) pathways supported by human data and upregulated under hypoxic conditions and (b) exclusion of pathways involving knockout experiments or chemical synthesis. After removing redundant gene sets, 7 hypoxia-related gene sets were retained (34).

High-confidence hypoxic and normoxic cell classification: Using single-sample gene set enrichment analysis, we calculated activity scores for each cell across the 7 hypoxia-related gene sets. A GMM was applied to classify cells into high- and low-scoring groups for each gene set. Cells consistently assigned to high-scoring groups across all 7 gene sets were classified as high-confidence hypoxic cells, while those consistently assigned to low-scoring groups were classified as high-confidence normoxic cells. The remaining cells were categorized as low confidence.

Classification of hypoxic states in low-confidence cells: To further differentiate hypoxic states in low-confidence cells, we developed a machine-learning classifier embedded with the LLM based on high-confidence cells. (a) Differential gene expression analysis: Wilcoxon’s rank-sum test was used to identify differentially expressed genes between high-confidence hypoxic and normoxic cells (*P* value < 0.05, LogFC > 0.25), retaining protein-coding genes, which resulted in 573 hypoxia signature genes. (b) To address the issue of gene loss, we utilized the LLM scGPT, specifically designed for single-cell transcriptomic data, to construct the embedding matrix. This model is based on the Transformer architecture, integrating a multihead attention mechanism and a custom attention masking strategy. During the pretraining phase, the model learns the regulatory relationships and coexpression patterns between genes. After the input data are provided, the model, based on the pretraining results, captures the associations between known genes through the attention mechanism and transfers them to the missing genes. Simultaneously, the attention masking strategy blocks irrelevant information, ultimately compressing the high-dimensional sparse matrix into a dense, gene-agnostic embedding matrix, effectively compensating for the missing data (35). (c) Classifier modeling and performance evaluation: We utilized 10 machine-learning algorithms, including Logistic Regression, eXtreme Gradient Boosting, Support Vector Machine, Random Forest, Light Gradient Boosting Machine, Naive Bayes, Decision Tree, Categorical Boosting, Multilayer Perceptron neural network, and Gradient Boosting Machine. Subsequently, we employed a 10%–100% stratified sampling strategy to partition training and test sets in a high-confidence single-cell cohort and 4 hypoxic cell line real-world independent validation cohorts. Accuracy, AUROC, and F1 score were then evaluated across 10 machine-learning models. Algorithms ranked in the top 3 across all metrics on average were intersected to identify CatBoost as the optimal classifier.

Application of the optimal classifier: The best-performing CatBoost model was used to classify hypoxic states in low-confidence cells.

### Statistics.

All data analysis, including processing, statistical evaluation, and plotting, was conducted using R software (version 4.3.1). Data normality and homogeneity of variance were assessed using Shapiro-Wilk and Levene’s tests, respectively, guiding the selection of either parametric tests (2-tailed paired *t* test, 2-tailed Student’s *t* test, Welch’s *t* test, 1-way ANOVA, 1-way ANOVA with Tukey’s post test) or nonparametric alternatives (Wilcoxon’s signed-rank, Mann-Whitney *U*, and Kruskal-Wallis tests). Categorical variables were analyzed using χ^2^ tests, while correlation analyses employed Pearson’s or Spearman’s methods as appropriate. Cox regression and Kaplan-Meier analysis were performed using the survival R package. All statistical tests were 2 sided. *P* value < 0.05 was regarded as statistically significant.

### Study approval.

All animal and human studies were approved by Zhengzhou University Life Science Institutional Review Board (ZZUIRB 2023-320). Animal procedures adhered to institutional guidelines, using 4- to 6-week-old BALB/c nude mice (both sexes; Vital River Laboratory Animal Technology). Human CRC tissues were collected from The First Affiliated Hospital of Zhengzhou University with patients’ informed consent.

### Data availability.

The scRNA-Seq and bulk RNA-Seq datasets used in this study are publicly accessible through the GEO database (https://www.ncbi.nlm.nih.gov/geo/) and the TCGA (https://www.cancer.gov/ccg/research/genome-sequencing/tcga). The GEO accession numbers are as follows: scRNA-Seq data GSE132465, GSE144735, GSE166555, and GSE200997; bulk RNA-Seq data: GSE35896, GSE92921, GSE143985, GSE75316, GSE18088, GSE26682, GSE13067, GSE4554, GSE17537, GSE17536, GSE77953, GSE41258, GSE18105, GSE21510, GSE71187, GSE25071, and GSE39582). Additionally, the ST datasets were retrieved from the scCRLM atlas (http://www.cancerdiversity.asia/scCRLM/), 10X Genomics (https://www.10xgenomics.com/), and the National Center for Biotechnology Information (https://www.ncbi.nlm.nih.gov/geo/query/acc.cgi?acc=GSE225857). The transcriptomic data are available at https://doi.org/10.7303/syn62787929 The source code for the TimeCCI tool is available on GitHub at https://github.com/Zaoqu-Liu/TimeCCI (commit ID: 183306c and commit URL: https://github.com/Zaoqu-Liu/TimeCCI/commit/183306c51f640581feaec26b21163d2e1ff05279). Values for all data points in graphs are reported in the Supporting Data Values file.

Additional methods applied in this study are available in Supplemental Methods.

## Author contributions

ZL conceived, designed, and supervised the research. XH and TP provided project guidance and funding support. CZ, ZL, and TP conducted the bioinformatics analysis. YB, AZ, SL, and HX performed the experiments, with YZ conducting the statistical analysis of experimental trials. CZ and JD wrote the manuscript. BL and SY provided samples. YW, TP, and JD provided technical assistance. TP, JD, PL, and QC offered research guidance. CZ, TP, JD, YZ, SW, YC, JN, LL, and XZ collected the data. ZL, JD, and TP revised the manuscript. The order of co–first authors was determined by the volume of work each contributed to the study.

## Supplementary Material

Supplemental data

Unedited blot and gel images

## Version 1 10/15/2025

Electronic publication

## References

[1] Bray F (2024). Global cancer statistics 2022: GLOBOCAN estimates of incidence and mortality worldwide for 36 cancers in 185 countries. CA Cancer J Clin.

[2] Gupta GP, Massagué J (2006). Cancer metastasis: building a framework. Cell.

[3] Biller LH, Schrag D (2021). Diagnosis and treatment of metastatic colorectal cancer: a review. JAMA.

[4] Pepper MS (2001). Lymphangiogenesis and tumor metastasis: myth or reality?. Clin Cancer Res.

[5] Compton CC, Greene FL (2004). The staging of colorectal cancer: 2004 and beyond. CA Cancer J Clin.

[6] Greene FL (2002). A new TNM staging strategy for node-positive (stage III) colon cancer: an analysis of 50,042 patients. Ann Surg.

[7] Choi GS, Kim HJ (2024). The role of lateral pelvic lymph node dissection in advanced rectal cancer: a review of current evidence and outcomes. Ann Coloproctol.

[8] Sasaki T (2021). Horizontal spread of pericolic lymph node metastasis as a prognostic factor for recurrence in stage III colorectal cancer. Colorectal Dis.

[9] Nelson H (2001). Guidelines 2000 for colon and rectal cancer surgery. J Natl Cancer Inst.

[10] Jiao S (2023). Prognostic impact of increased lymph node yield in colorectal cancer patients with synchronous liver metastasis: a population-based retrospective study of the US database and a Chinese registry. Int J Surg.

[11] Caligiuri G, Tuveson DA (2023). Activated fibroblasts in cancer: perspectives and challenges. Cancer Cell.

[12] Kalluri R (2016). The biology and function of fibroblasts in cancer. Nat Rev Cancer.

[13] Zhang Y (2025). Cancer-associated fibroblast-derived SEMA3C facilitates colorectal cancer liver metastasis via NRP2-mediated MAPK activation. Proc Natl Acad Sci U S A.

[14] Liu L (2025). FOS-driven inflammatory CAFs promote colorectal cancer liver metastasis via the SFRP1-FGFR2-HIF1 axis. Theranostics.

[15] Zheng H (2024). Roles of cancer-associated fibroblast functional heterogeneity in shaping the lymphatic metastatic landscape: new insights and therapeutic strategies. Cancer Biol Med.

[16] Null JL (2023). Periostin+ stromal cells guide lymphovascular invasion by cancer cells. Cancer Res.

[17] Zheng H (2024). PDGFRα^+^ITGA11^+^ fibroblasts foster early-stage cancer lymphovascular invasion and lymphatic metastasis via ITGA11-SELE interplay. Cancer Cell.

[18] Kashima H (2019). Cancer-associated fibroblasts (CAFs) promote the lymph node metastasis of esophageal squamous cell carcinoma. Int J Cancer.

[19] Wei WF (2023). Cancer-associated fibroblast-derived PAI-1 promotes lymphatic metastasis via the induction of EndoMT in lymphatic endothelial cells. J Exp Clin Cancer Res.

[20] Yan J (2024). Cancer-associated fibroblasts promote lymphatic metastasis in cholangiocarcinoma via the PDGF-BB/PDGFR-β mediated paracrine signaling network. Aging Dis.

[21] Liu T (2019). Cancer-associated fibroblasts: an emerging target of anti-cancer immunotherapy. J Hematol Oncol.

[22] Lavie D (2022). Cancer-associated fibroblasts in the single-cell era. Nat Cancer.

[23] De Visser KE, Joyce JA (2023). The evolving tumor microenvironment: from cancer initiation to metastatic outgrowth. Cancer Cell.

[24] Wagner A (2016). Revealing the vectors of cellular identity with single-cell genomics. Nat Biotechnol.

[25] Kotliar D (2019). Identifying gene expression programs of cell-type identity and cellular activity with single-cell RNA-Seq. Elife.

[26] Veghini L (2024). Differential activity of MAPK signalling defines fibroblast subtypes in pancreatic cancer. Nat Commun.

[27] Ji RC (2014). Hypoxia and lymphangiogenesis in tumor microenvironment and metastasis. Cancer Lett.

[28] Chen XJ (2021). A novel lymphatic pattern promotes metastasis of cervical cancer in a hypoxic tumour-associated macrophage-dependent manner. Angiogenesis.

[29] Rofstad EK (2002). Hypoxia promotes lymph node metastasis in human melanoma xenografts by up-regulating the urokinase-type plasminogen activator receptor. Cancer Res.

[30] Choi JI (2023). Hypoxic microenvironment determines the phenotypic plasticity and spatial distribution of cancer-associated fibroblasts. Clin Transl Med.

[31] Schwörer S (2023). Hypoxia potentiates the inflammatory fibroblast phenotype promoted by pancreatic cancer cell-derived cytokines. Cancer Res.

[32] Dou B (2023). Machine learning methods for small data challenges in molecular science. Chem Rev.

[33] Tian T (2019). Clustering single-cell RNA-seq data with a model-based deep learning approach. Nat Mech Intell.

[34] Zhang Y (2023). Single-cell RNA sequencing identifies critical transcription factors of tumor cell invasion induced by hypoxia microenvironment in glioblastoma. Theranostics.

[35] Cui H (2024). scGPT: toward building a foundation model for single-cell multi-omics using generative AI. Nat Methods.

[36] Kostyunina DS (2024). Transcriptomics and proteomics revealed sex differences in human pulmonary microvascular endothelial cells. Physiol Genomics.

[37] Zhang Y (2023). Identifying key genes related to the peritubular capillary rarefaction in renal interstitial fibrosis by bioinformatics. Sci Rep.

[38] De Oliveira KG (2024). Decoding of the surfaceome and endocytome in primary glioblastoma cells identifies potential target antigens in the hypoxic tumor niche. Acta Neuropathol Commun.

[39] Patel AP (2014). Single-cell RNA-seq highlights intratumoral heterogeneity in primary glioblastoma. Science.

[40] Efremova M (2020). CellPhoneDB: inferring cell-cell communication from combined expression of multi-subunit ligand-receptor complexes. Nat Protoc.

[41] Zhou X (2024). THBS2 + cancer-associated fibroblasts promote EMT leading to oxaliplatin resistance via COL8A1-mediated PI3K/AKT activation in colorectal cancer. Mol Cancer.

[42] Topalovski M et al (2016). Hypoxia and Transforming Growth Factor β Cooperate to Induce Fibulin-5 Expression in Pancreatic Cancer. J Biol Chem.

[43] De Francesco EM (2013). HIF-1α/GPER signaling mediates the expression of VEGF induced by hypoxia in breast cancer associated fibroblasts (CAFs). Breast Cancer Res.

[44] Ziani L (2021). Hypoxia increases melanoma-associated fibroblasts immunosuppressive potential and inhibitory effect on T cell-mediated cytotoxicity. Oncoimmunology.

[45] Sun K (2019). Oxidized ATM-mediated glycolysis enhancement in breast cancer-associated fibroblasts contributes to tumor invasion through lactate as metabolic coupling. EBioMedicine.

[46] Kumar L M EF (2007). Mfuzz: a software package for soft clustering of microarray data. Bioinformation.

[47] Zhang F (2020). Unsupervised inference of developmental directions for single cells using VECTOR. Cell Rep.

[48] Qiu X (2017). Reversed graph embedding resolves complex single-cell trajectories. Nat Methods.

[49] Cao EY (2020). GeneSwitches: ordering gene expression and functional events in single-cell experiments. Bioinformatics.

[50] Aibar S (2017). SCENIC: single-cell regulatory network inference and clustering. Nat Methods.

[51] Chen Z (2023). Hypoxic microenvironment in cancer: molecular mechanisms and therapeutic interventions. Signal Transduct Target Ther.

[52] Zheng N (2023). Multiregion single cell analysis reveals a novel subtype of cancer-associated fibroblasts located in the hypoxic tumor microenvironment in colorectal cancer. Transl Oncol.

[53] Mello AM (2022). Hypoxia promotes an inflammatory phenotype of fibroblasts in pancreatic cancer. Oncogenesis.

[54] Zhang Q (2020). hTFtarget: a comprehensive database for regulations of human transcription factors and their targets. Genomics Proteomics Bioinformatics.

[55] Liberzon A (2011). Molecular signatures database (MSigDB) 3.0. Bioinformatics.

[56] Wu Z (2023). CRIP1 reshapes the gastric cancer microenvironment to facilitate development of lymphatic metastasis. Adv Sci (Weinh).

[57] Liu Z (2025). THBS2-producing matrix CAFs promote colorectal cancer progression and link to poor prognosis via the CD47-MAPK axis. Cell Rep.

[58] Hu Y (2016). A machine learning approach for the identification of key markers involved in brain development from single-cell transcriptomic data. BMC Genomics.

[59] Aybey B (2023). Immune cell type signature discovery and random forest classification for analysis of single cell gene expression datasets. Front Immunol.

[60] Ma Q, Xu D (2022). Deep learning shapes single-cell data analysis. Nat Rev Mol Cell Biol.

[61] Xu Y (2021). Hypoxia facilitates the proliferation of colorectal cancer cells by inducing cancer-associated fibroblast-derived IL6. Neoplasma.

[62] Giaccia AJ, Schipani E (2010). Role of carcinoma-associated fibroblasts and hypoxia in tumor progression. Curr Top Microbiol Immunol.

[63] Wang M (2020). Molecular structure, expression, and functional role of Clec11a in skeletal biology and cancers. J Cell Physiol.

[64] Yue R (2016). Clec11a/osteolectin is an osteogenic growth factor that promotes the maintenance of the adult skeleton. Elife.

[65] Lin TY (2022). EGFR mutation-harboring lung cancer cells produce CLEC11A with endothelial trophic and tumor-promoting activities. Cancers (Basel).

[66] Zheng Q (2024). Identification and characterization of CLEC11A and its derived immune signature in gastric cancer. Front Immunol.

[67] Zhu Y, Li X (2023). Advances of Wnt signalling pathway in colorectal cancer. Cells.

[68] Zhao H (2022). Wnt signaling in colorectal cancer: pathogenic role and therapeutic target. Mol Cancer.

[69] Cho YH (2020). 5-FU promotes stemness of colorectal cancer via p53-mediated WNT/β-catenin pathway activation. Nat Commun.

[70] Hao Y (2021). Integrated analysis of multimodal single-cell data. Cell.

[71] McGinnis CS (2019). DoubletFinder: doublet detection in single-cell RNA sequencing data using artificial nearest neighbors. Cell Syst.

[72] Korsunsky I (2019). Fast, sensitive and accurate integration of single-cell data with Harmony. Nat Methods.

