# Supplementary material for: Cancer-associated fibroblasts enhance colorectal cancer lymphatic metastasis via CLEC11A/LGR5-mediated WNT pathway activation
Source: J Clin Invest. 2025 Oct 15;135(20):e194243. doi: 10.1172/JCI194243 (PMC12520694; doi:10.1172/JCI194243)
Supplement: Supplemental data [file jci-135-194243-s343.pdf]

Cancer-associated fibroblasts enhance colorectal cancer lymphatic metastasis via CLEC11A/LGR5-mediated WNT pathway activation

Supplemental figures 1-16

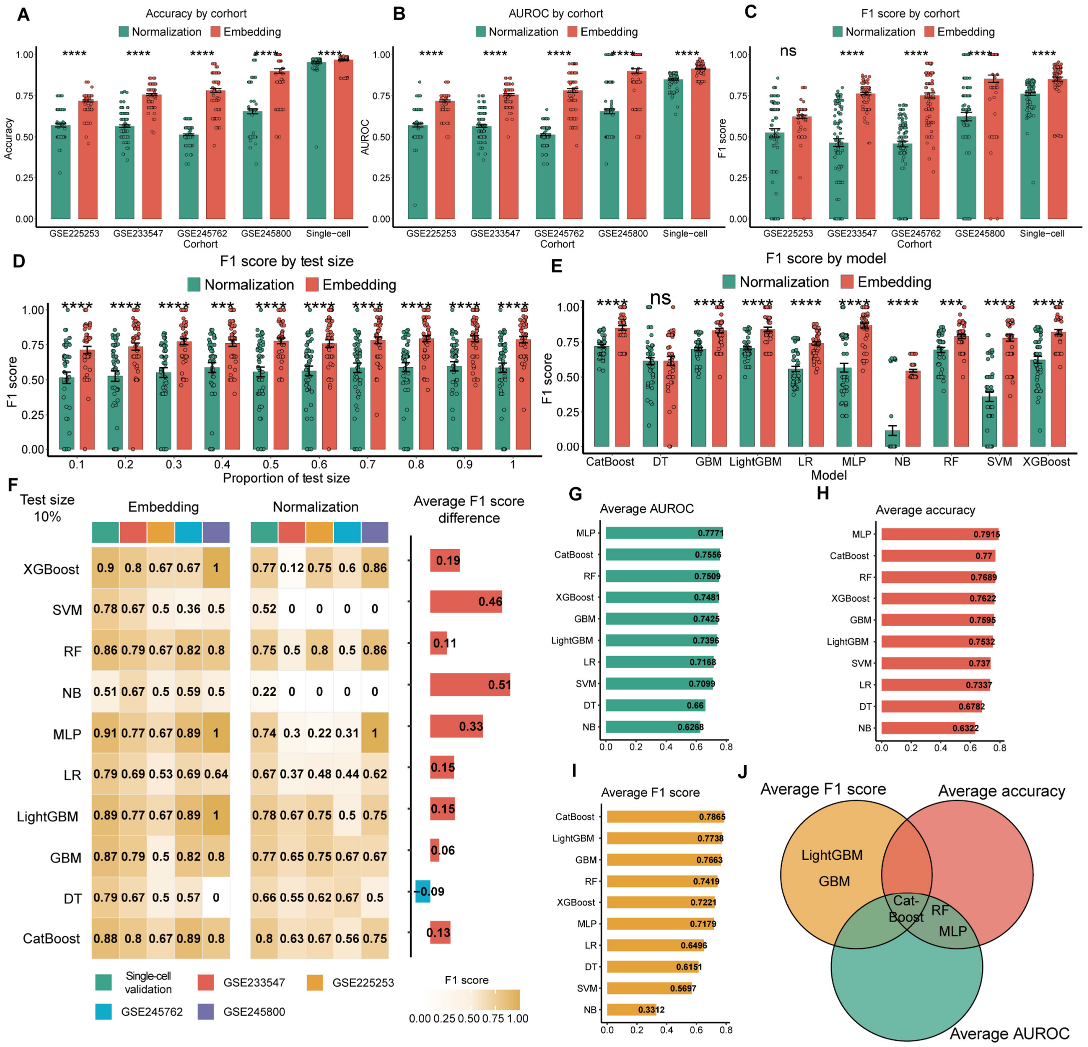

Supplemental figure 1. Construction and validation of the cellular hypoxia predicting classifier (CHPC) based on the large language model (LLM).

A-C. Differences in accuracy (**A**), AUROC (**B**), and F1 score (**C**) between the two matrices across various datasets.

D-E. Differences in F1 score between the two matrices across varying test set sizes (**D**) and different machine-learning models (**E**).

F. Differences in F1 score between the two matrices across various machine-learning models and datasets when using 10% of the training data.

G-I. Ranking of AUROC (**G**), accuracy (**H**)and F1 score (**I**) for cell embedding matrices across ten machine-learning models.

J. Venn diagram showing the intersection of the top three rankings across the three metrics.

All data are presented as means ± SEM. $^{***}P < 0.001$, $^{****}P < 0.0001$, by Shapiro-Wilk testing, normally distributed data were analyzed using Paired t-test, whereas non-normally distributed data were examined by Wilcoxon signed-rank test (**A-E**).

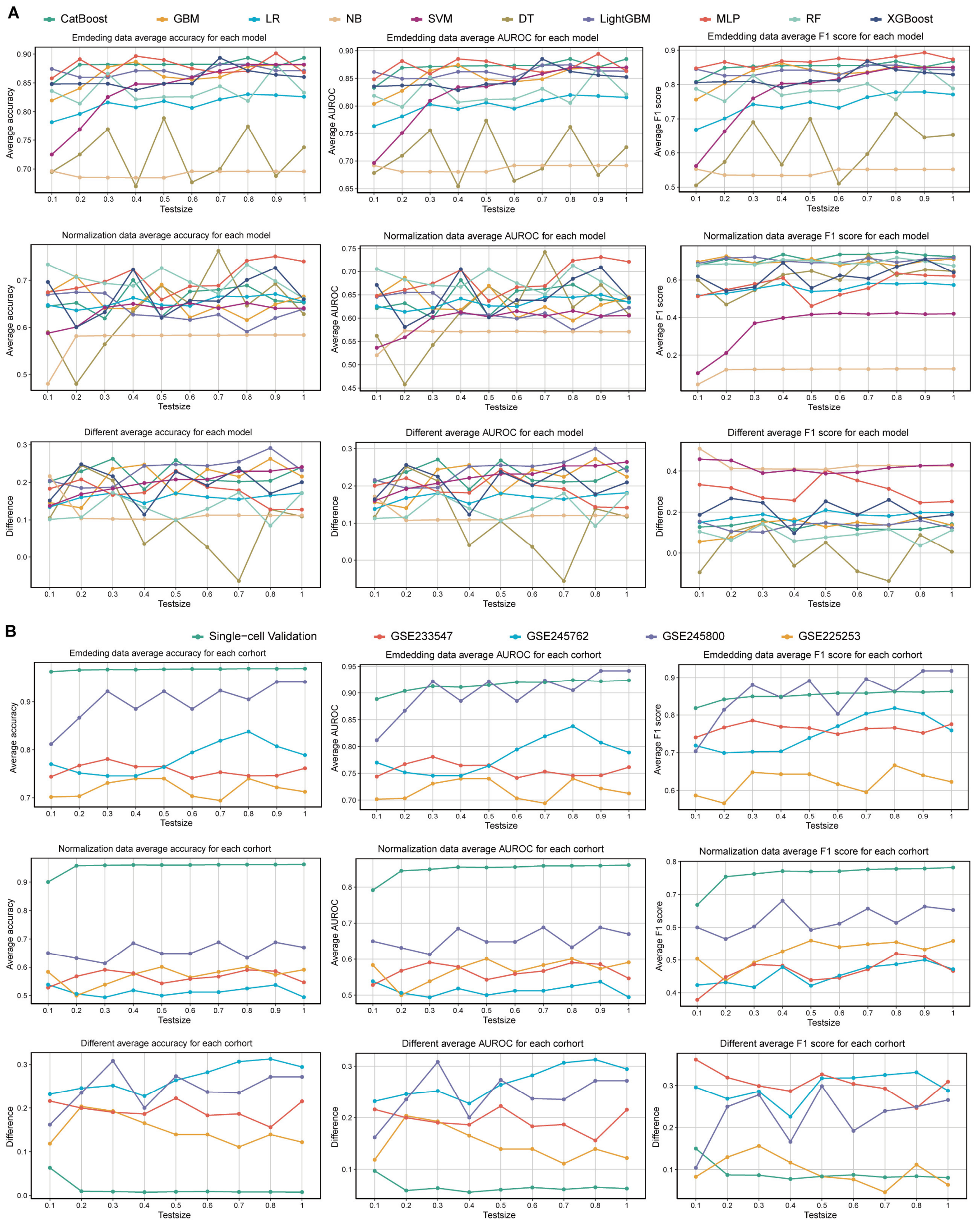

Supplemental figure 2. Comparison of predictive metrics between the two matrices.

A. Line plots illustrating accuracy (left), AUROC (center), and F1 score (right) for the cell embedding matrix (top), normalized matrix (middle), and their differences (bottom) across various machine-learning models and test set sizes.

B. Line plots showing accuracy (left), AUROC (center), and F1 score (right) for the cell embedding matrix (top), normalized matrix (middle), and their differences (bottom) across different datasets and test set sizes.

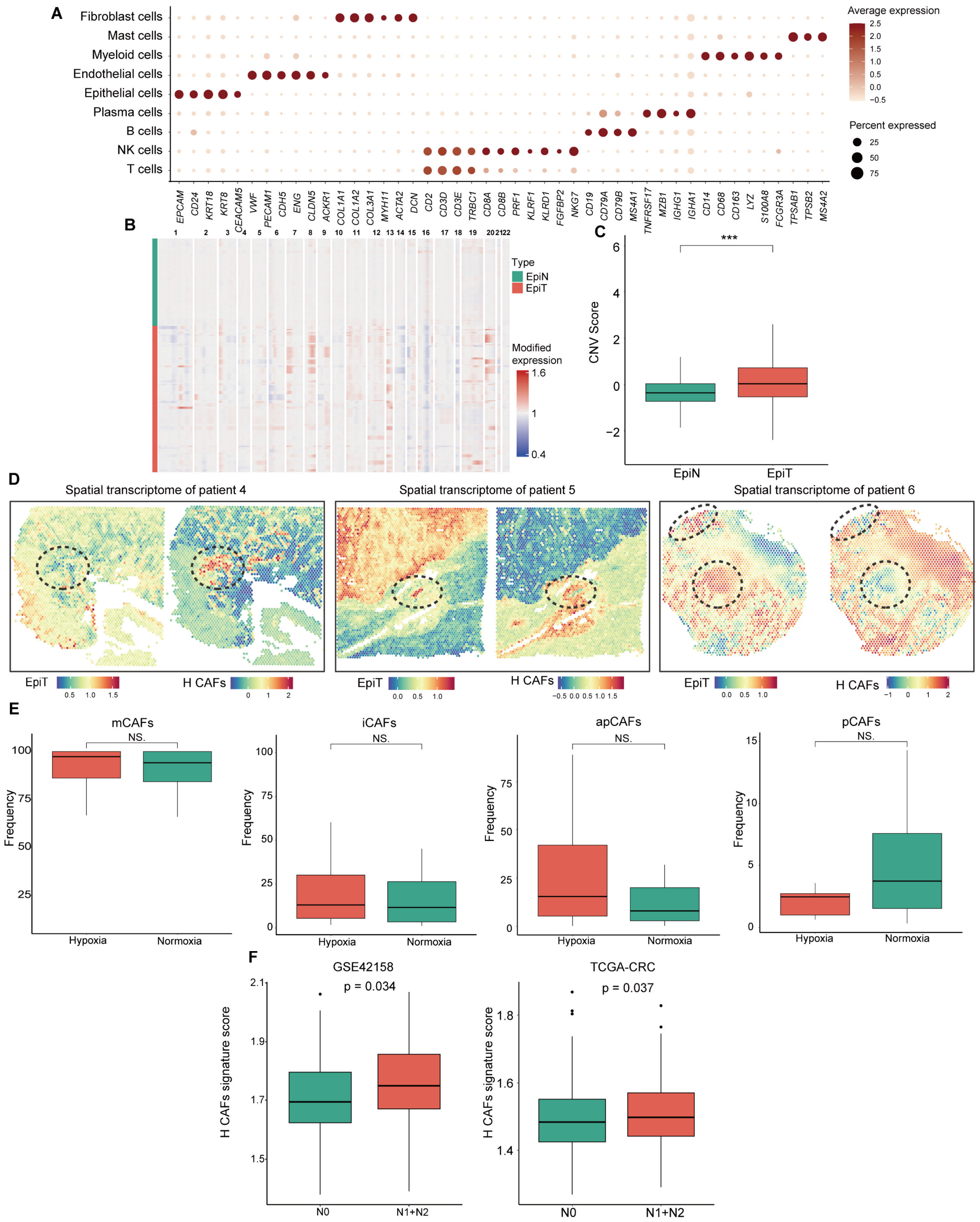

Supplemental figure 3. HCAFs interact with tumor cells and are associated with lymphatic metastasis.

A. Dot plot showing the expression of classical cell type markers across identified cell populations.

B. The heatmap displaying the chromosomal map of large-scale copy number variations in epithelial cells inferred through scRNA-seq.

C. The box plot displaying the CNV scores of epithelial cells.

D. Spatial transcriptomics revealing the spatial proximity between HCAFs and EpiT.

E. The box plot showing the proportion of hypoxic and normoxic cells across different CAF types

F. The box plot showing the distribution of HCAF proportions across different N stages in the GSE42158 and TCGA-CRC cohorts.

****P* < 0.001, by Mann-Whitney U test (**C** and **E**), and Student's t-test (**F**)

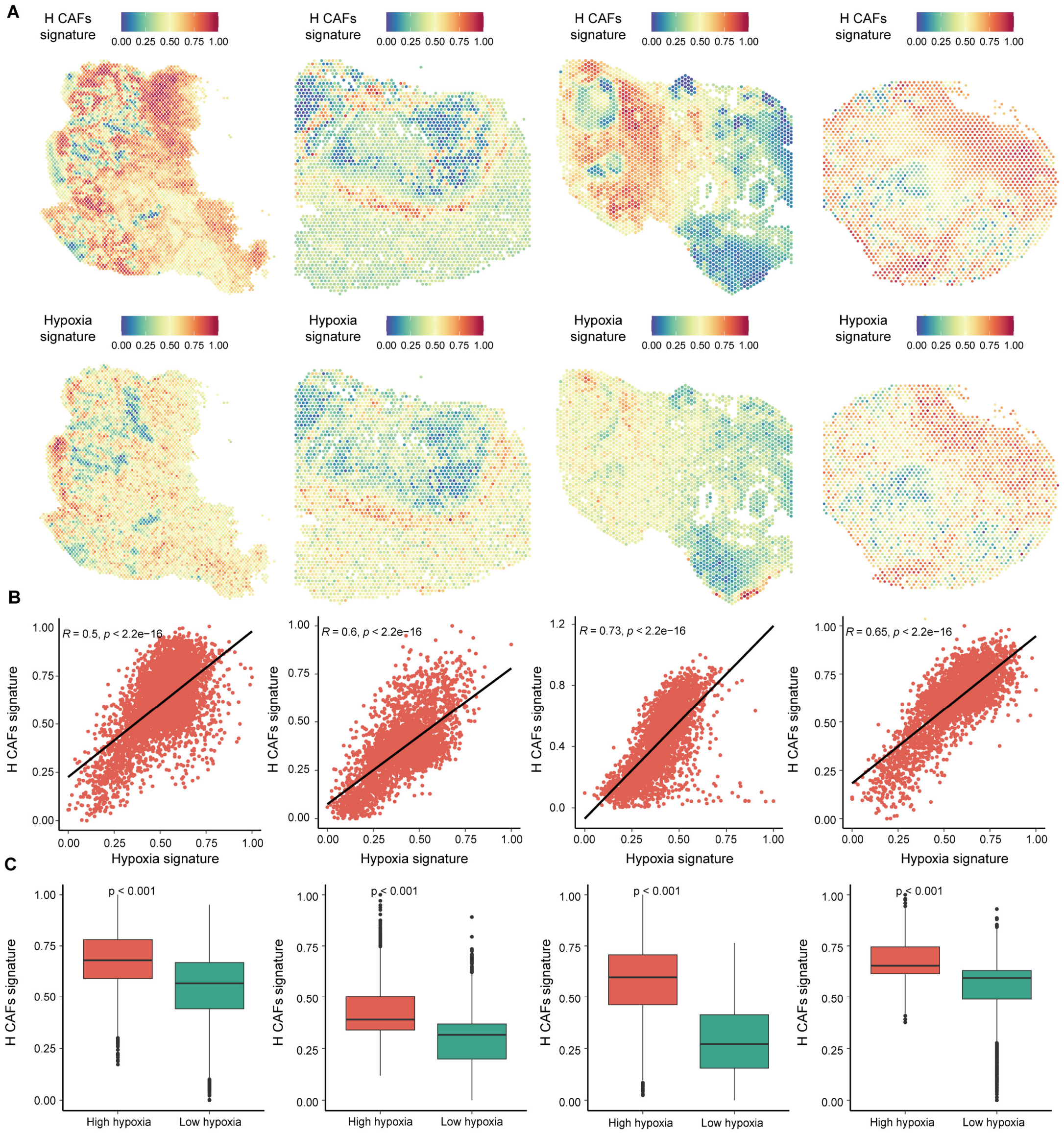

Supplemental figure 4. Spatial transcriptome distribution of HCAFs and hypoxic regions in tumors.

A. Spatial transcriptomic distribution of HCAFs signature and Hallmark hypoxia pathway signature in four CRC tumor slices.

B. Correlation between HCAFs signature and Hallmark hypoxia pathway signature (corresponding by column).

C. Expression of HCAFs signature in hypoxic regions classified by Hallmark hypoxia pathway signature (corresponding by column).

Spearman's rank correlation test (**B**) and Mann-Whitney U test (**C**).

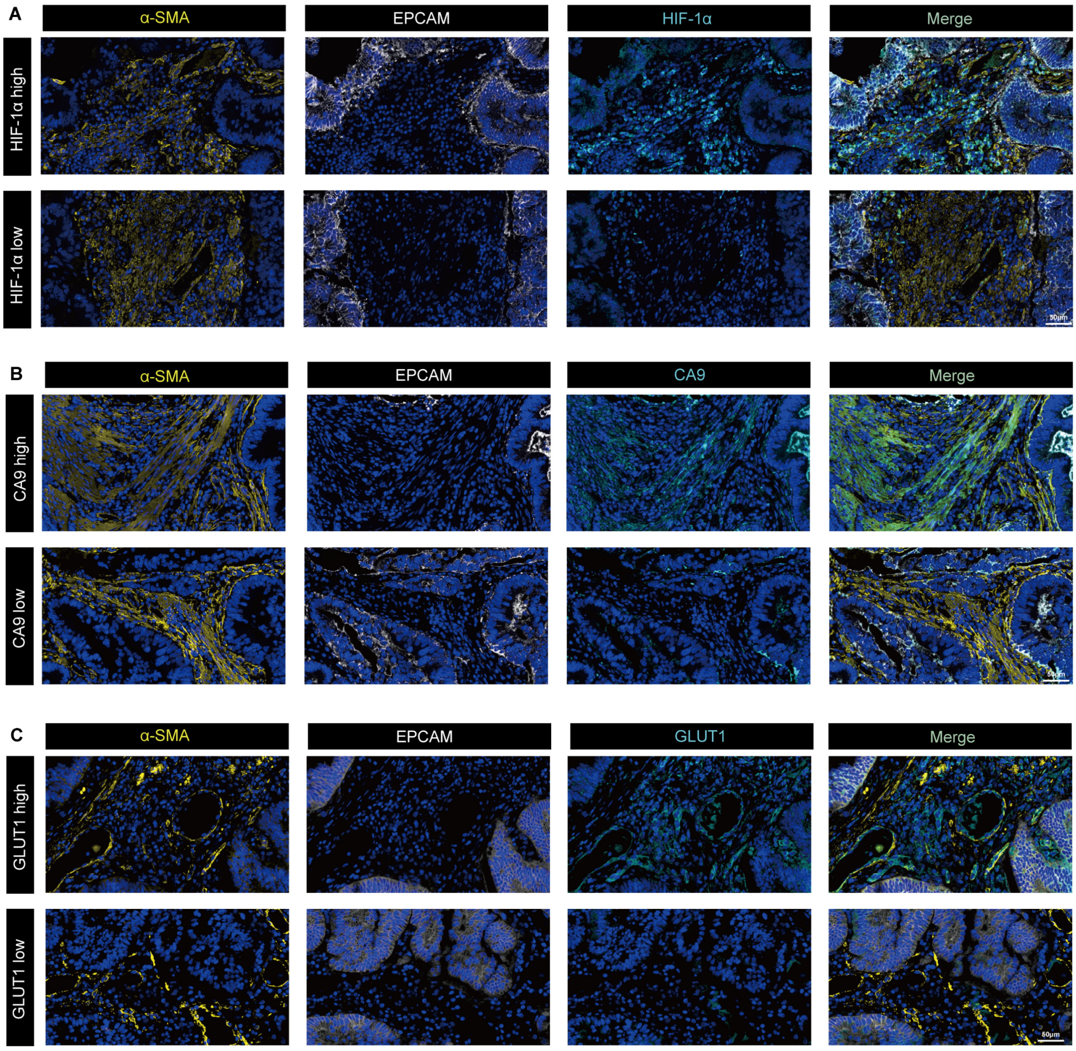

Supplemental figure 5. Distribution of hypoxic areas and HCAFs in tumor tissues.

A. Multiplex immunohistochemistry (mIHC) revealing the co-localization of α-SMA, EPCAM, and HIF-1α.

B. mIHC revealing the co-localization of α-SMA, EPCAM, and CA9.

C. mIHC revealing the co-localization of α-SMA, EPCAM, and GLUT1.

Scale bar: 50 μm (**A, B** and **C**).

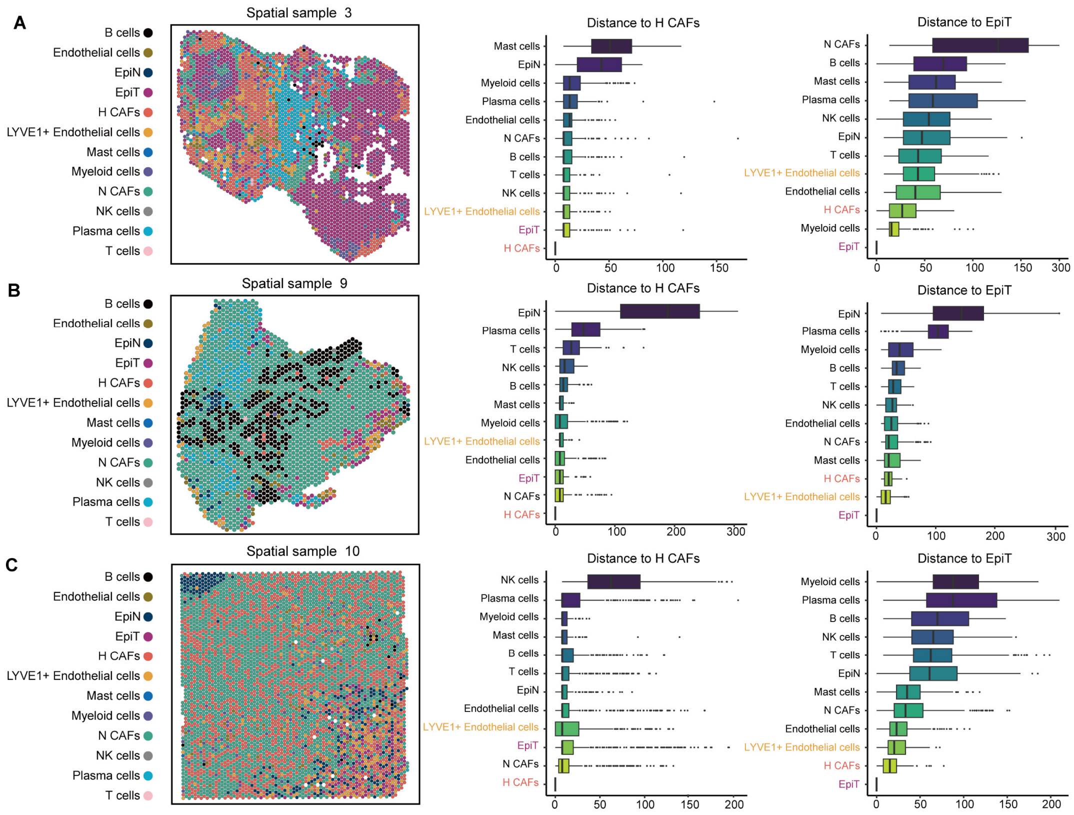

Supplemental figure 6. Spatial distance from other cells to HCAFs and tumor cells. A-C. Spatial transcriptomic cell type identification based on the Robust Cell Type Decomposition (RCTD) algorithm (left panel). The minimum spatial distances from various cell types to HCAFs (middle panel) and tumor cells (right panel) were quantified.

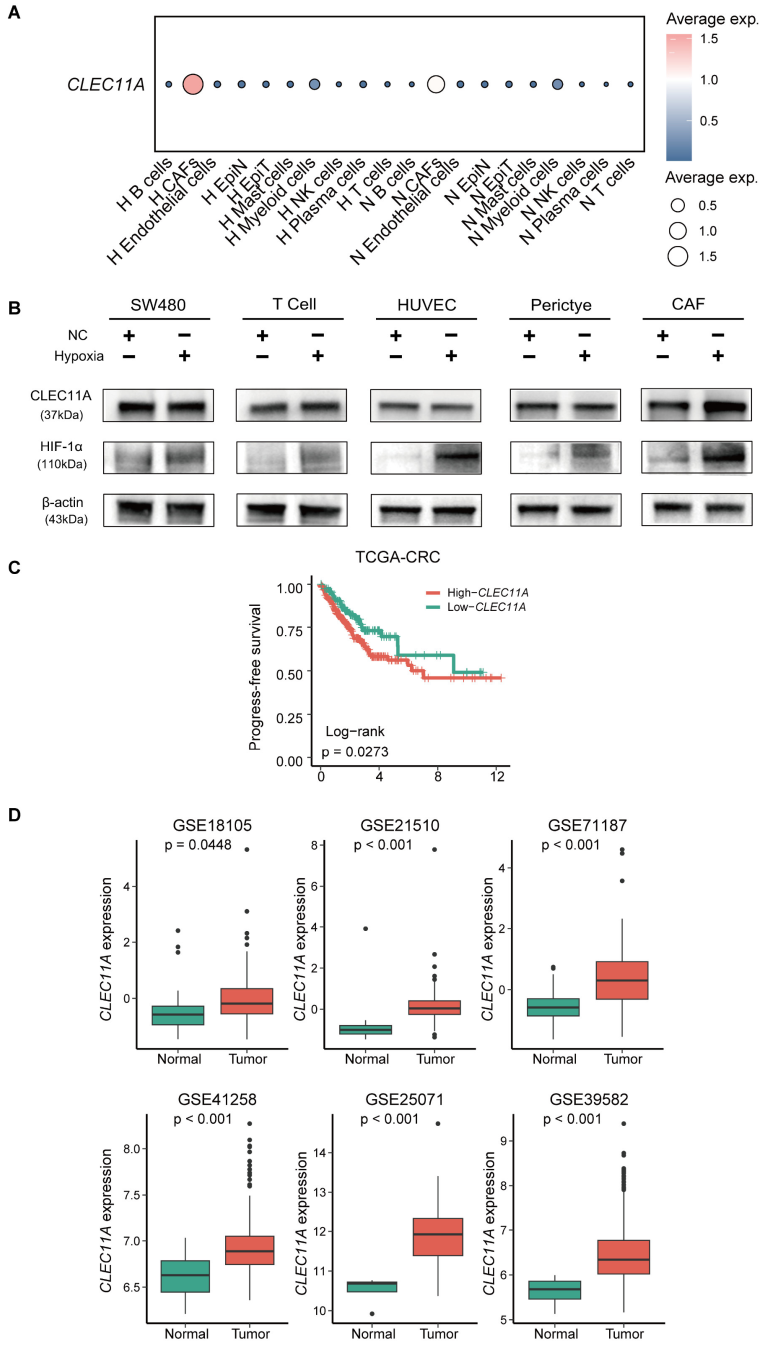

Supplemental figure 7. Cellular Expression Specificity and Clinical Significance of CLEC11A

A. *CLEC11A* expression across distinct cell populations in the single-cell dataset.

B. Western blot analysis of CLEC11A and HIF1-α expression under normoxic and hypoxic conditions.

C. The Kaplan-Meier survival curve from the TCGA-CRC dataset indicates that the progression-free survival (PFS) of patients with high expression of *CLEC11A* is significantly poor.

D. Multiple independent transcriptomic datasets showing that *CLEC11A* is highly expressed in colorectal cancer tumor tissues.

Log-rank test (**C**), Student's t-test (**D: GSE25071**) or Mann-Whitney U test (**D**).

		HR	Lower 95% CI	Upper 95% CI	*P* value
CLEC11A	*n* = 90	1.032	1.013	1.052	*P* <0.001
Age	*n* = 90	1.013	0.985	1.041	0.367
Gender	Female (*n* = 42)	Reference			
	Male (*n* =47)	1.318	0.685	2.536	0.408
T	T1/T2 (*n* = 8)	Reference			
	T3/T4 (*n* = 75)	2.297	0.659	8.006	0.192
N	N0 (*n* = 58)	Reference			
	N1/N2 (*n* = 32)	1.176	0.588	2.353	0.647
M	M0 (*n* = 88)	Reference			
	M1 (*n* = 2)	10.139	1.142	90.032	0.038

No.events: 55; global *P* value (log-rank): 2.966×10^{-27}

AIC: 366.692; concordance index: 0.690

Supplemental figure 8. Multivariate Cox regression analysis of overall survival (OS) in the TMA cohort (*n* = 90) evaluated the CLEC11A protein expression along with age, gender, local invasion depth (T), lymph node involvement (N), and distant metastasis extent (M). P values for individual covariates were obtained using the Wald test.

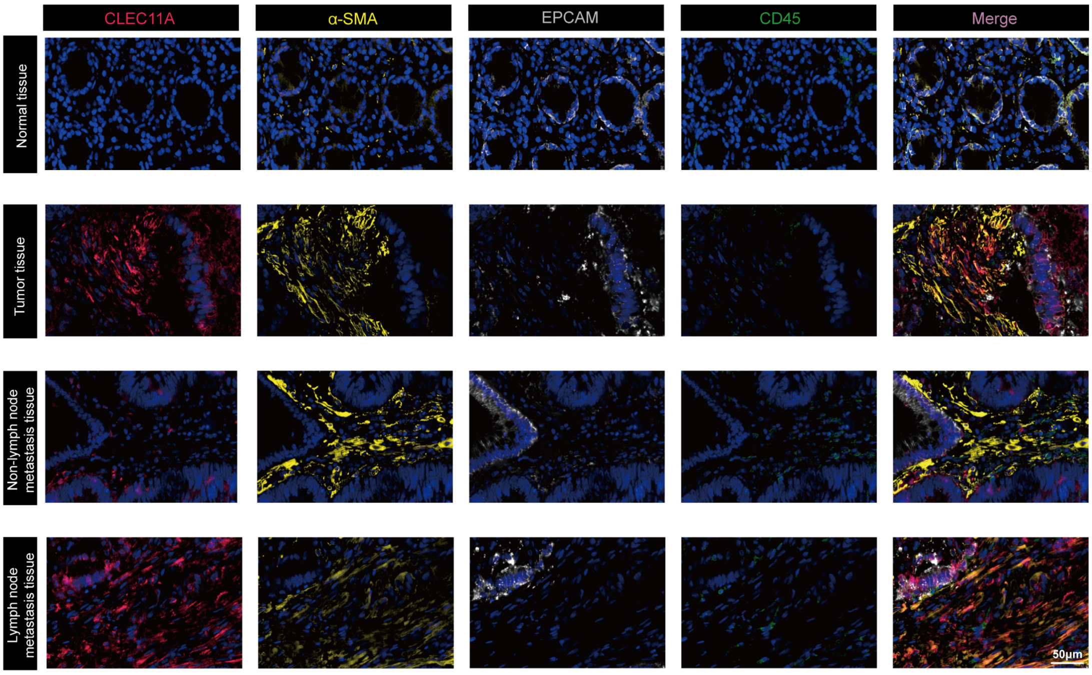

Supplemental figure 9. Multiplex immunohistochemistry analysis of CLEC11A expression in colorectal tissues. Scale bar: 50 μm.

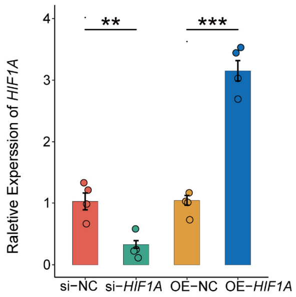

Supplemental figure 10. qPCR analysis of *HIF1A* mRNA levels in CAFs with HIF1A knockdown or overexpression (*n*=4 per group). All data are presented as means ± SEM. ***P* < 0.01, ****P* < 0.001, by 1-way ANOVA test with Tukey's post-test.

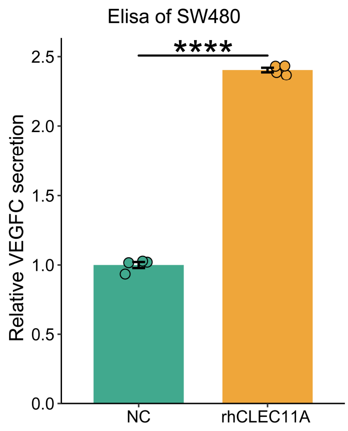

Supplemental figure 11. ELISA quantification of VEGFC levels in SW480 cells treated with rhCLEC11A (*n*=4 per group). All data are presented as means ± SEM. *******P* < 0.0001, by Welch's t test.

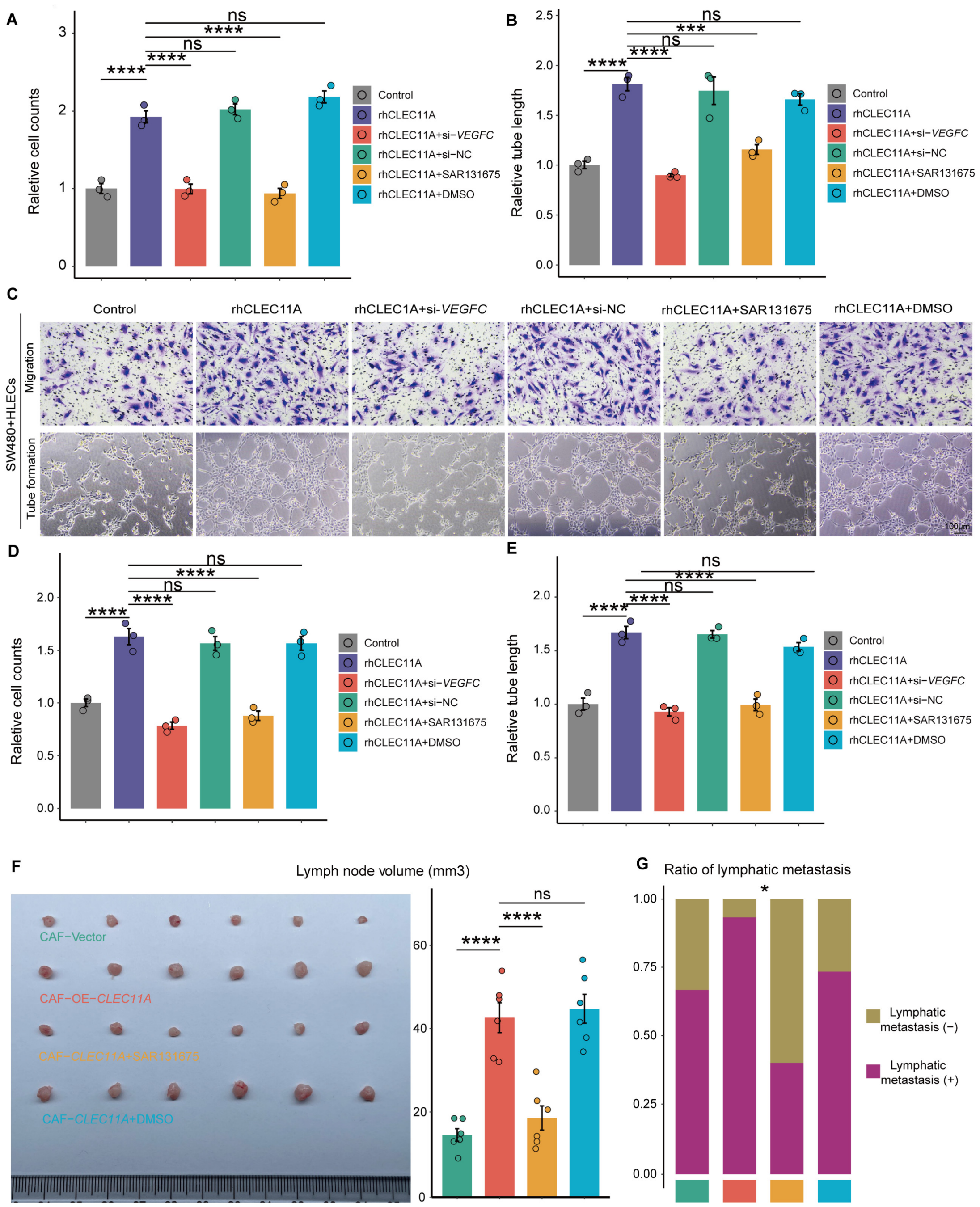

Supplemental figure 12. CLEC11A promotes EMT and VEGFC production in tumor cells, leading to lymphangiogenesis and lymphatic metastasis.

A-B. Histograms showing HLEC migration (**A**) and tube formation (**B**) after culture with HCT116 cell line-conditioned media under different treatments (*n*=3 per group).

C. Representative images of HLEC tube formation and migration assays cultured in conditioned media under specific treatments.

D-E. Histograms showing HLEC migration (**D**) and tube formation (**E**) after culture with SW480 cell line-conditioned media under different treatments (*n*=3 per group).

F. Representative images of popliteal lymph nodes from the mouse metastasis model established using SW480 cells co-injected with CAFs subjected to specific treatments. Histograms quantify lymph node volumes (mm^3) in nude mice (*n*=6 per group).

G. Ratio of metastasis to total dissected lymph nodes in mice inoculated with specific CAFs and SW480 cells (*n* = 15 per group).

Scale bar: 100 μm (**C**). All data are presented as means ± SEM. $*P < 0.05$, $***P < 0.001$, $****P < 0.0001$, by 1-way ANOVA test with Tukey's post-test (**A, B, D, E** and **F**) and Chi-square test (**G**).

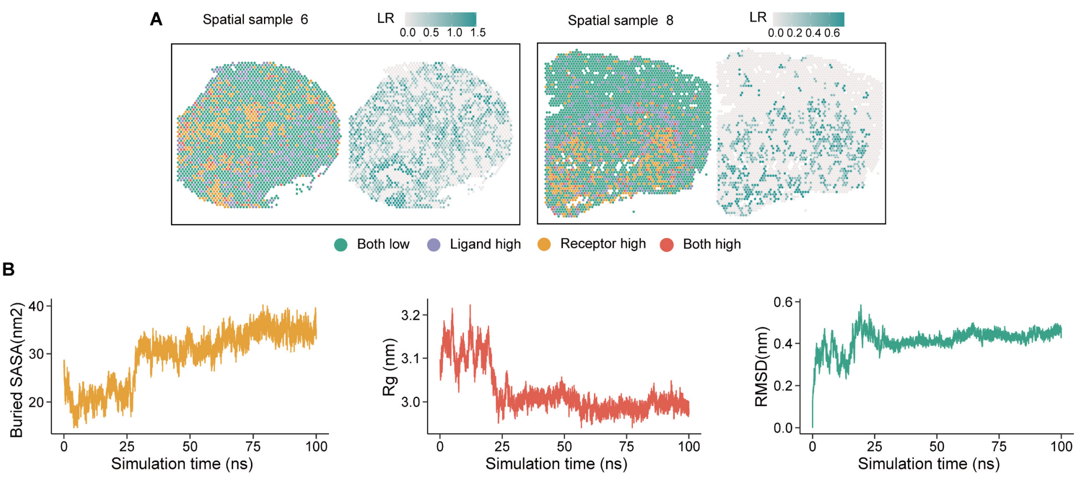

Supplemental figure 13. CLEC11A interacts with LGR5 on tumor cells.

A. ST data showing CLEC11A-LGR5 interactions

B. Quantitative analysis of the CLEC11A-LGR5 complex stability over a 100-nanosecond simulation.

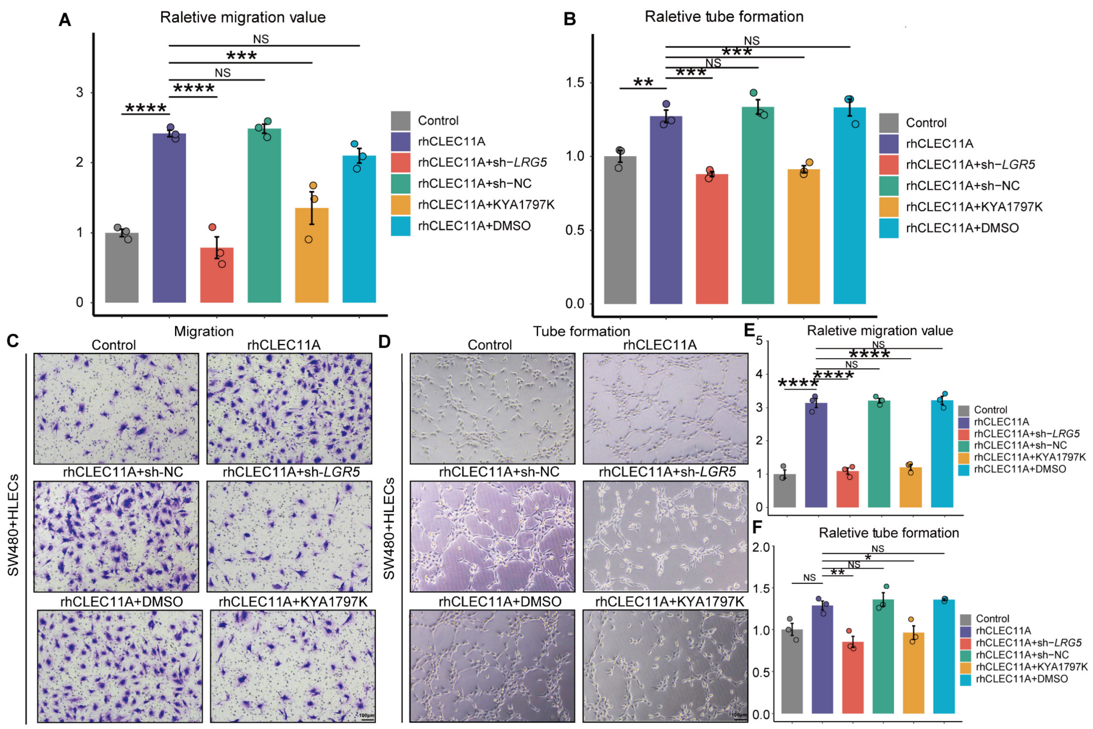

Supplemental figure 14. CLEC11A activates the WNT/β-catenin pathway via LGR5 on tumor cells to promote EMT and VEGFC secretion.

A-B. Histograms showing HLEC migration (**A**) and tube formation (**B**) after culture with HCT116 cell line-conditioned media under different treatments (n=3 per group).

C-D. Representative images of HLEC migration (**C**) and tube formation (**D**) assays in response to conditioned media from SW480 cells treated under specific conditions.

E-F. Histograms showing HLEC migration (**E**) and tube formation (**F**) after culture with SW480 cell line-conditioned media under different treatments (n=3 per group).

Scale bar: 100 μm (**C** and **D**). All data are presented as means ± SEM. $*P < 0.05$, $**P < 0.01$, $***P < 0.001$, $****P < 0.0001$, by 1-way ANOVA test with Tukey's post-test (**A, B, E** and **F**).

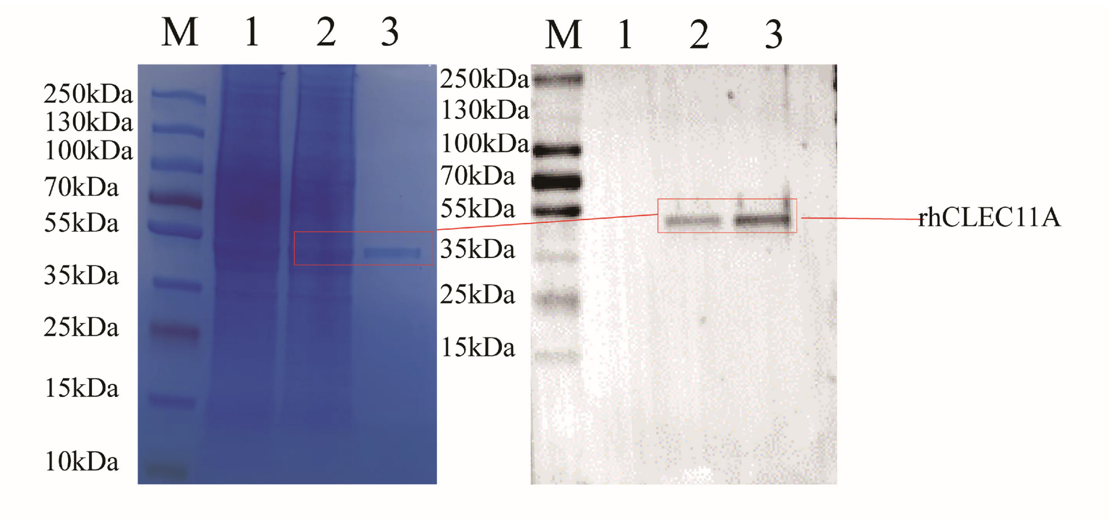

Supplemental figure 15. Construction of recombinant proteins.

In the SDS-PAGE analysis, Lane M represents the protein marker; Lane 1 contains the lysate from E. coli transformed with pQE-80L/hCLEC11A before induction; Lane 2 shows the lysate after induction; and Lane 3 contains the purified recombinant proteins (rhCLEC11A). In the Western blot, Lane 1 corresponds to the pre-induction lysates, which were not detected by the anti-His tag monoclonal antibody (McAb), whereas Lanes 2 and 3, representing the post-induction lysates and purified proteins, were successfully recognized by the antibody.

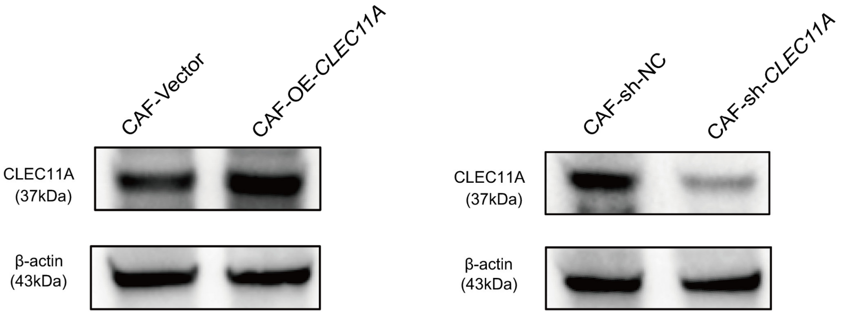

Supplemental figure 16. Western blot analysis of CLEC11A expression in established stable CAF cell lines.

Cancer-associated fibroblasts enhance colorectal cancer lymphatic metastasis via CLEC11A/LGR5-mediated WNT pathway activation

Supplemental methods

Migration assays

For the migration assays, 2×10^4 transfected HLEC cells were plated in the upper chamber of Transwell inserts, with 500 μL of complete medium added to the lower chamber. Fixation of the migrated cells was carried out using 4% paraformaldehyde (Beyotime, Shanghai, China), followed by staining with crystal violet (Solarbio, Beijing, China). The stained cells were subsequently examined and imaged using an optical microscope (Cewei, Shanghai, China). ImageJ software was utilized to quantify the number of migrated cells.

Lymphatic endothelial cell tube formation assay

The pipette tips and 24-well plates were pre-chilled, and the growth factor-reduced Matrigel (BD, Corning, USA) was left to thaw overnight. Matrigel was applied to the wells of a 24-well plate and incubated at 37°C for 30 minutes to solidify. Prior to the assay, 2×10^4 HUVECs were seeded into the gel and incubated with tumor cell or fibroblast-conditioned medium for 12 hours. Tube formation of HLECs was observed under a microscope, and the total branching length was quantified using ImageJ software.

Chromatin immunoprecipitation (ChIP)

ChIP was conducted to assess the interaction between *HIF1A* and the *CLEC11A* promoter using the ChIP-IT Express Kit (Active Motif, Shanghai, China) following the manufacturer's protocol. In brief, cells were fixed at room temperature for 10 minutes to crosslink histones to DNA. Following this, the cells were rinsed twice with cold PBS and shaken briefly for 5 seconds. Once the PBS was discarded, glycine stop solution was introduced to terminate the fixation process, and the plate was shaken for an additional 5 minutes at room temperature. The cells were collected using chilled cell scraping buffer, centrifuged for 10 minutes at low temperature, and lysed in 1 ml of pre-chilled lysis buffer for 30 minutes to isolate the nuclei. Sonication of the chilled lysate was performed, and soluble chromatin was obtained by centrifugation and resuspended in ChIP dilution buffer. The supernatant was saved as a DNA input control, while the

remainder was immunoprecipitated using HIF-1α (Abcam, Shanghai, China) and IgG antibodies (Proteintech, 10284-1-AP). "Immunoprecipitated complexes and protein A beads were incubated overnight at 4°C, followed by sequential washing with ChIP Buffers I and II. Crosslinking between DNA and proteins was reversed using reverse crosslinking buffer, and DNA was purified via phenol-chloroform extraction for PCR analysis. All primer sequences employed in this analysis are systematically listed in Table S1.

Luciferase reporter assay

Luciferase activity was quantified using the Dual-Luciferase Reporter Assay (Beyotime, Shanghai, China), following the guidelines provided by the manufacturer. Post-transfection, cells were lysed directly in the culture dishes using a specific lysis buffer. The lysates were centrifuged at maximum speed for 1 minute in a microcentrifuge to clarify the samples. Measurements of luciferase activity were conducted using a Modulus™ TD20/20 Luminometer (Turner Biosystems, USA). To adjust for differences in transfection efficiency, Renilla luciferase activity was utilized as an internal control for normalization.

Transendothelial migration assay (TrEM)

HLECs (50,000 cells) were seeded into an 8 μm pore transwell insert pre-coated with 2 $\mu g/cm^2$ fibronectin. Once the HLECs formed a confluent monolayer, 5×10^4 RFP-labeled HCT116 or SW480 cells were added to the upper chamber containing the endothelial monolayer. Following 24 hours of incubation, the non-migrated cells in the upper chamber were discarded, and cells that migrated to the bottom of the insert were fixed with 4% formaldehyde. One random field per insert was captured using an inverted fluorescence microscope, and the transendothelial migrating cells were quantified with ImageJ software based on the fluorescence images.

Histopathological analyses

Colorectal cancer tissue microarrays (OD-CT-DgRec02-001, n = 32; HCol-Ade180Sur-06, n = 180) were purchased from Outdo Biotech (Shanghai, China).

Tissue sections for immunohistochemical staining were first deparaffinized in xylene and then rehydrated through a graded alcohol series. Antigen retrieval was carried out using EDTA buffer (pH 8.0). To block non-specific binding, the sections were incubated with PBS containing 5% bovine serum albumin (BSA) for 30 minutes at room temperature. Next, the sections were incubated with primary antibodies overnight at 4°C. Following extensive washing, the sections were treated with suitable secondary antibodies for 1 hour at room temperature. Visualization was achieved using DAB (Servicebio, Wuhan, China), followed by counterstaining with hematoxylin. Additionally, Hematoxylin-eosin (H&E) was conducted using the Hematoxylin-eosin HD constant dye kit (Servicebio, Wuhan, China) as the manufacturer's instructions. Bright-field images were captured using an ECLIPSE E100 microscope (Nikon, Tokyo, Japan), and digital scanning was performed with a Pannoramic MIDI (3DHISTECH, Budapest, Hungary).

The slides for multiplex immunohistochemistry were initially deparaffinized and rehydrated, and antigen retrieval was then performed using microwave treatment. The endogenous peroxidase activity and non-specific binding sites were subsequently blocked. Primary antibodies were applied, followed by secondary HRP-conjugated polymers from the TSA fluorescent staining kit. The covalent reaction was subsequently followed by additional antigen retrieval to eliminate any background signal before proceeding to the next step. After several cycles of labeling, nuclear counterstaining with DAPI was performed to complete the staining procedure. Color images were acquired using the ZEISS AXIOSCAN slide scanning system (ZEISS, Oberkochen, Germany).

Preparation of recombinant protein

The cDNA sequence for human CLEC11A (hCLEC11A) was obtained from GenBank (NM_002975.3). The plasmid pQE-80L/hCLEC11A was constructed by Sangon

Biotech (Shanghai, China). rhCLEC11A was expressed in Escherichia coli BL21(DE3) strains, induced with 0.5 mM isopropyl β-D-1-thiogalactopyranoside (IPTG) (Sangon Biotech, Shanghai, China) at 28°C for 12 hours. rhCLEC11A was purified using nickel affinity chromatography (Sangon Biotech, Shanghai, China). Expression of rhCLEC11A was confirmed by SDS-PAGE and Western blot analysis. Detailed antibody information is provided in Table S2.

Co-immunoprecipitation (Co-IP)

Co-immunoprecipitation (Co-IP) was performed using the Pierce™ Crosslink Magnetic IP/Co-IP Kit (Thermo Fisher Scientific, 88805, Massachusetts, USA) according to the manufacturer's instructions. SW480 cells were incubated with human recombinant CLEC11A protein for 48 hours, then lysed in IP buffer supplemented with protease and phosphatase inhibitors. For immunoprecipitation, 10 μg of CLEC11A-specific antibody or species-matched normal IgG (negative control) was first incubated with Protein A/G magnetic beads overnight at 4°C with gentle rotation to allow antibody conjugation. The antibody-bead complexes were then incubated with the whole-cell lysate at room temperature for 2 hours under gentle rotation to facilitate the formation of immune complexes. The immunoprecipitated proteins were eluted by heating in SDS sample buffer following extensive washing to remove non-specifically bound proteins. Western blot analysis was performed using total cell lysates as input, while normal IgG served as the negative control.

Immunofluorescence

Cells were seeded and subjected to specified treatments prior to fixation with 4% paraformaldehyde at room temperature. Blocking was performed with 5% bovine serum albumin (BSA) in PBS. The cells were then incubated overnight at 4°C with primary antibodies. Following PBS washing, the cells were treated with the appropriate secondary antibodies for 1 hour at room temperature. Nuclei were stained with DAPI. The slides were mounted, and observations were made using a NIKON ECLIPSE C1

microscope (Nikon, Tokyo, Japan). Images were documented using a Pannoramic MIDI scanner (3DHISTECH, Budapest, Hungary).

Rhodamine phalloidin staining method

Cell samples were fixed in 4% PFA, treated with 0.1% Triton X-100 for 10 minutes to permeabilize, and then blocked with 5% BSA for 1 hour. Rhodamine phalloidin (R415, Invitrogen, Carlsbad, California, U.S.A) was dissolved in methanol at 6.6 μM and stored at −20 °C. Just prior to use, phalloidin was diluted (1:40) in 1% BSA. The samples were incubated with diluted phalloidin for 30 min. Nuclei were stained with DAPI by incubation for 10 min. Then, the samples were washed three times with PBS for 5 min each. Images of F-actin were captured on a Pannoramic MIDI scanner (3DHISTECH, Budapest, Hungary).

Western blot

The cells were cultured under designated protein or medium conditions, followed by lysis on ice for 30 minutes with RIPA buffer containing protease inhibitors (Beyotime, Shanghai, China). To collect total protein, the lysates were centrifuged at 12,000 rpm for 20 minutes at 4°C. Protein samples (30 μg each) were separated using SDS-PAGE and subsequently transferred onto PVDF membranes (Millipore, Massachusetts, USA). Membranes were blocked using a rapid blocking solution for 20 minutes at room temperature, followed by a 1-hour incubation with primary antibodies. HRP-conjugated secondary antibodies were used for detection, and signals were visualized with Super ECL Plus (UElandy, Suzhou, China) on a Tanon chemiluminescent imaging system (Tanon, Shanghai, China). Detailed antibody information is provided in Table S2.

Enzyme-linked immunosorbent assay (ELISA)

Cells were cultured in 10 cm dishes at a density of 4×10^6 cells per dish for 36 hours. Following incubation, the culture medium was collected and centrifuged at 1,000 rpm for 5 minutes at 4°C to remove cellular debris. The supernatant was transferred to a new labeled tube, and 100 μL was diluted tenfold for the assay. CLEC11A and VEGFC

levels were quantified using CLEC11A ELISA Kit (JL18140-96T, JONLNBIO, Shanghai, China) and VEGF-C ELISA Kit (EK1154, MULTI SCIENCES, Zhejiang, China), according to the manufacturer's instructions.

RNA isolation, reverse transcription, and qPCR

Total RNA was extracted using TRIzon reagent (CWbiotech, Jiangsu, China), and complementary DNA (cDNA) synthesis was performed using the HiFiScript cDNA Synthesis Kit (CWbiotech, Jiangsu, China). Quantitative PCR was conducted on a LineGene 9600 Plus Real-Time PCR System (Bioer, Hangzhou, China) with RapidStart Universal SYBR Green qPCR Mix (Monad, Suzhou, China). Relative mRNA expression levels were calculated using the cycle threshold (Ct) method, normalized to GAPDH, and quantified by the 2−ΔΔCt method. Primer sequences used in the analysis are provided in Table S3.

Cell–cell interaction analysis

Cellular communication analysis was performed with CellphoneDB, on the basis of the ligand-receptor interactions in different cell types. The normalized gene expression matrix and cell type metadata were used as input for CellphoneDB. The significance threshold of *P*-value <0.05 was established to identify meaningful ligand-receptor interactions.

To identify potential ligand-receptor pairs between source and target cells, we first calculated potential receptors specifically expressed in target cells, and then extend receptor-ligand pairs by adding these receptors and target ligands in source cells to the communication database. TimeCCI, a computational tool previously developed to elucidate dynamic cellular communication patterns, was used to quantify the temporal correlation of potential ligand expression (https://github.com/Zaoqu-Liu/TimeCCI). By integrating the expression profiles of the two cell types, we used Monocle to reconstruct developmental trajectories. Subsequently, the interpolated pseudotime series was sampled utilizing the CellAlign framework, and the spearman correlation coefficient (SCC) was computed for each ligand-receptor pair throughout the pseudotime

continuum. Finally, based on the normalized expression data of potential ligand-receptor pairs with high SCC scores, we employed CellChat to calculate the communication probabilities between source and target cells for these potential pairs.

Trajectory analysis

The VECTOR and Monocle R packages were used for single-cell trajectory analysis to infer the differentiation trajectories of CAFs under different hypoxic conditions. The pseudo-time calculated by the Monocle algorithm was used for enrichment analysis and other calculations. The GeneSwitches tool was employed to identify regulatory switches in the evolution of fibroblast hypoxic states, detecting switch genes and transcription factors that potentially play regulatory roles during this process.

Transcription factors (TFs) speculation

To identify the core TFs in HCAFs, we conducted the SCENIC analysis. Initially, the GENIE3 algorithm was employed to infer the gene regulatory network from the input single-cell expression matrix. Next, we used RcisTarget for DNA-motif analysis to identify potential regulators, scoring the motifs of gene promoter regions 500 bp upstream and 10 kb surrounding the transcription start sites (TSS) using the hg38 database. Finally, the regulatory activity of potential transcription factors was analyzed using AUCell, and the transcription factor cell-type specificity score (RSS) was computed.

Biological enrichment analysis

Differential expression analysis of genes was carried out using the limma R package on bulk transcriptome data, while in scRNA-seq data, FindMarkers was employed. Subsequently, we utilized the clusterProfiler R package to assess the enrichment of pathway activity, referencing the Molecular Signatures Database (MSigDB, https://www.gsea-msigdb.org/gsea/msigdb) and the Gene Ontology (GO) Biological Process Ontology (http://geneontology.org).

Spatial transcriptomics data collection and analysis

The ST datasets for CRC, was sourced from the scCRLM atlas (http://www.cancerdiversity.asia/scCRLM/), 10x Genomics (https://www.10xgenomics.com/) and GSE225857. Publicly available spatial transcriptomics (ST) datasets were processed using the R package Seurat. Spots with fewer than 300 gene counts or mitochondrial gene fractions exceeding 30% were flagged as low quality and removed. To reveal the cell type enrichment within the ST spots, the AddModuleScore function provided by the Seurat platform was applied to execute the required computation. Additionally, we used the SpaGene R package to identify the spatial colocalization of potential ligand-receptor pairs.

Bulk transcriptome data collection and analysis

A total of 12 bulk transcriptome datasets from patients with CRC, along with clinical information, were obtained from the Cancer Genome Atlas (TCGA) and GEO. Data preprocessing and normalization followed previous research methods. Subsequently, based on specific cell markers of HCAFs, we utilized ssGSEA from the GSVA R package to assess the infiltration level of HCAFs in bulk transcriptome datasets. To identify genes exhibiting differential expression patterns correlated with N stage, we employed the Mfuzz R package to cluster gene expression profiles related to N stage in TCGA-CRC dataset.

Four cell line datasets (GSE233547, GSE225253, GSE245762, GSE245800) containing hypoxic and normoxic cells were used to evaluate the accuracy of the LLM-based classifier in identifying hypoxic cells after transcripts per million (TPM) normalization.

Cancer-associated fibroblasts enhance colorectal cancer lymphatic metastasis via CLEC11A/LGR5-mediated WNT pathway activation

Supplemental tables

Supplemental table 1. Detailed information of primer sequences used in chromatin immunoprecipitation	
**Genes**	**Primer sequences**
CLEC11A	1F:GAAAGAGGAGGGTGTGCGAG
	1R:CTCTGTGTGTGTCTCTCCCC

Supplemental table 2. Detailed information of antibodies used in this study		
**Antibodies**	**Source**	**Item number**
VE-cadherin antibody	Affiniity	Cat No : AF6265
E-cadherin antibody	Proteintech	Cat No : 20874-1-AP
N-cadherin antibody	Proteintech	Cat No : 22018-1-AP
Vimentin antibody	Proteintech	Cat No : 10366-1-AP
ZEB1 antibody	Proteintech	Cat No : 21544-1-AP
VEGFC antibody	Santa Cruz Biotechnology	Cat No : sc-130289
CLEC11A antibody	Proteintech	Cat No : 60295-1-Ig
HIF-1 alpha Polyclonal antibody	Proteintech	Cat No :20960-1-AP
LGR5 Polyclonal antibody	Proteintech	Cat No :30007-1-AP
β-catentin Polyclonal antibody	Cell Signaling Technology	Cat No :8480S
IgG	Proteintech	Cat No : 10283-1-AP
Alpha smooth muscle actin specific Recombinant antibody	Proteintech	Cat No : 80008-1-RR
Anti-LYVE1 antibody	Abcam	Cat No : ab219556
LYVE1 Monoclonal Antibody	Invitrogen	Cat No : 14-0443-82
Cytokeratin 20 Polyclonal antibody	Proteintech	Cat No : 17329-1-AP
Anti-pan Cytokeratin antibody	Abcam	Cat No : ab215838
Anti-HIF-1 alpha antibody	Abcam	Cat No : ab51608
VEGF-C Polyclonal antibody	Proteintech	Cat No : 22601-1-AP
His-Tag	Proteintech	Cat No : 66005-1-Ig

β-actin	Proteintech	Cat No :66009-1-Ig
HRP-conjugated Affinipure Goat Anti-Mouse IgG(H+L)	Proteintech	Cat No : SA00001-1
HRP-conjugated Affinipure Goat Anti-Rabbit IgG(H+L)	Proteintech	Cat No : SA00001-2

Supplemental table 3. Detailed information of primer sequences used in qPCR

**Genes**	**Primer sequences**
VEGFC	F: GCCAATCACACTTCCTGCCGAT
	R:AGGTCTTGTTCGCTGCCTGACA
VEGFD	F:GACTGGAAGCTGTGGAGATGCA
	R:GGCTGCACTGAGTTCTTTGCCA
CLEC11A	F:ACACCCGCGATGCCGTGCAAG
	R:CGAGAGCAGGAAGCACTTGTGG
HIF1A	F:TATGAGCCAGAAGAACTTTTAGGC
	R:CACCTCTTTTGGCAAGCATCCTG
GAPDH	F:GTCTCCTCTGACTTCAACAGCG
	R: ACCACCCTGTTGCTGTAGCCAA

Supplemental table 4. *CLEC11A* promoter fragment used in this study

***CLEC11A* promoter fragment**
GCTGAATGCTCAGAGCTCCTTGAGCACACAACCTAGCGTCTGTCGATTTCACCTCCTGGAGAAACCTTCCCTGACTGCTCCGCCAGACTCCTCACCCTCTCCCATCTCCAGCCCCCTCATCCCCGGAGCTATCTGGCTGCTGGTCTGCAAGCAGCTGGAGTCCCCAGCATCACCCAGCCCACAGCCTGGCCTGCAGGAGGCTTCTAGTAAAGTGGCCAATTAATCCCCGAGCCCCGTCTTCCAGGGTCATGCACACAGTCACAC***GCGTGC***TCTTCACATGTGTCACCCTGCCCCCACACACACTCACTTGGATCTCCAGACAAGGGCTTGGAGGGTAGGATGGGCAATCTAGGGGGCAGGAGGGGCCCTGGAGTCTCCTCCATTGAGTGTGCAGGCGATGGTGGCCACAGGGTAATAACAACCTCAGCGAGACCCTGAGGTCTGCCCCTCCTTAGGAATGGCCAGGCAGGGAAGAAGGGAGCAGTGCTCGGTCCCAGCATCCCACCACTGCCGAGAT
***CLEC11A* promoter-MUT fragment**
CCTTGAGCACACAACCTAGCGTCTGTCGATTTCACCTCCTGGAGAAACCTTCCCTGACTGCTCCGCCAGACTCCTCACCCTCTCCCATCTCCAGCCCCCTCATCCCCGGAGCTATCTGGCTGCTGGTCTGCAAGCAGCTGGAGTCCCCAGCATCACCCAGCCCACAGCCTGGCCTGCAGGAGGCTTCTAGTAAAGTGGCCAATTAATCCCCGAGCCCCGTCTTCCAGGGTCATGCACACAGTCACAC*AAAAAA*TCTTCACATGTGTCACCCTGCCCCCACACACACTCACTTGGATCTCCAGACAAGGGCTTGGAGGGTAGGATGGGCAATCTAGGGGGCAGGAGGGGCCCTGGAGTCTCCTCCATTGAGTGTGCAGGCGATGGTGGCCACAGGGTAATAACAACCTCAGCGAGACCCTGAGGTCTGCCCCTCCTTAGGAATGGCCAGGCAGGGAAGAAGGGAGCAGTGCTCGGTCCCAGCATCCCACCACTGCCGAGAT

Supplemental table 5. Hypoxia-related gene sets
**ELVIDGE_HYPOXIA_UP**
ADGRE2,ADM,ADORA2B,AHNAK2,AK4,AKAP12,ALDOC,ANG,ANGPTL4,ANKZF1,ARTN,ASPH,ATF3,ATG14,ATXN1,B3GNT4,BBX,BCOR,BHLHE40,BNIP3,BNIP3L,CA9,CADM1,CAV1,CAVIN1,CCN1,CCN5,CCNG2,CD59,CEMIP,CITED2,CSGALNACT1,CSRP2,CXCR4,CYB5A,CYP1B1,DAAM1,DDIT4,DDR1,DPYSL2,DPYSL4,DSC2,DST,DTNA,DUSP1,ECE1,EFNA3,EGFR,EGLN1,EGLN3,EGR1,ELF3,ENO2,ERO1A,FAM13A,FAM162A,FAM216A,FLNB,FOS,FYN,GADD45B,GBE1,GDF15,GJA1,GLRX,GPR87,GPRC5A,GYS1,HCFC1R1,HEY1,HILPDA,HK2,HLA-DRB1,IGFBP3,IGFBP5,ILVBL,INHA,INSIG2,ISG20,ITPR1,JUN,KDM3A,KDM4B,KLF6,KLF7,KLHL24,KRT15,KRT7,LIMCH1,LOX,LOXL1,LOXL2,MAFF,MET,MT-ND5,MXI1,NAP1L1,NDRG1,NFIL3,NOL3,NR3C1,NREP,OBSL1,OLFML2A,OPN3,ORAI3,P4HA1,P4HA2,PAM,PDGFB,PDGFRL,PDK1,PFKFB3,PFKP,PGAP1,PGK1,PGM1,PIM1,PLAC8,PLAUR,PLIN2,PPFIA4,PRKCA,PXDN,QSOX1,RASA4,RBCK1,RBPJ,RLF,RNASE4,RRAGD,S100A2,S100A4,S100A6,SAMD4A,SAT1,SCARB1,SCNN1B,SERPINE1,SFXN3,SH3GL3,SLC2A1,SLCO4A1,SORL1,SOX9,SPAG4,SPOCK1,SPRY1,SRD5A3,SRPX,STBD1,STC1,STC2,TBC1D3F,TGFBI,TIPARP,TMEFF1,TMEM265,TMEM45A,TNFAIP8,TXNIP,UPK1A,VEGFA,VEGFC,VLDLR,WSB1,YEATS2,YPEL1,ZMYND8,ZNF292,ZNF395,ZNF654
**FARDIN_HYPOXIA_11**
AK4,ALDOC,ANGPTL4,ANKRD37,BHLHE40,BNIP3,BNIP3L,BTG1,DDIT4,EGLN1,EGLN3,FAM162A,FUT11,IGF1R,IGFBP3,KDM3A,MAPT,MTFP1,MXI1,NDRG1,P4HA2,PDK1,PFKFB4,PGK1,PGM1,PLOD1,SLC2A3,TNIP1,TPI1,TXNIP,VEGFA,ZNF395
**HALLMARK_HYPOXIA**
ACKR3,ADM,ADORA2B,AK4,AKAP12,ALDOA,ALDOB,ALDOC,AMPD3,ANGPTL4,ANKZF1,ANXA2,ATF3,ATP7A,B3GALT6,B4GALNT2,BCAN,BCL2,BGN,BHLHE40,BNIP3L,BRS3,BTG1,CA12,CASP6,CAV1,CAVIN1,CAVIN3,CCN1,CCN2,CCN5,CCNG2,CDKN1A,CDKN1B,CDKN1C,CHST2,CHST3,CITED2,COL5A1,CP,CSRP2,CXCR4,DCN,DDIT3,DDIT4,DPYSL4,DTNA,DUSP1,EDN2,EFNA1,EFNA3,EGFR,ENO1,ENO2,ENO3,ERO1A,ERRFI1,ETS1,EXT1,F3,FAM162A,FBP1,FOS,FOSL2,FOXO3,GAA,GALK1,GAPDH,GAPDHS,GBE1,GCK,GCNT2,GLRX,GPC1,GPC3,GPC4,GPI,GRHPR,GYS1,HAS1,HDLBP,HEXA,HK1,HK2,HMOX1,HOXB9,HS3ST1,HSPA5,IDS,IER3,IGFBP1,IGFBP3,IL6,ILVBL,INHA,IRS2,ISG20,JMJD6,JUN,KDELR3,KDM3A,KIF5A,KLF6,KLF7,KLHL24,LALBA,LARGE1,LDHA,LDHC,LOX,LXN,MAFF,MAP3K1,MIF,MT1E,MT2A,MXI1,MYH9,NAGK,NCAN,NDRG1,NDST1,NDST2,NEDD4L,NFIL3,NOCT,NR3C1,P4HA1,P4HA2,PAM,PCK1,PDGFB,PDK1,PDK3,PFKFB3,PFKL,PFKP,PGAM2,PGF,PGK1,PGM1,PGM2,PHKG1,PIM1,PKLR,PKP1,PLAC8,PLAUR,PLIN2,PNRC1,PPARGC1A,PPFIA4,PPP1R15A,PPP1R3C,PRDX5,PRKCA,PYGM,RBPJ,RORA,RRAGD,S100A4,SAP30,SCARB1,SDC2,SDC3,SDC4,SELENBP1,SERPINE1,SIAH2,SLC25A1,SLC2A1,SLC2A3,SLC2A5,SLC37A4,SLC6A6,SRPX,STBD1,STC1,STC2,SULT2B1,TES,TGFB3,TGFBI,TGM2,TIPARP,TKTL1,TMEM45A,TNFAIP3,TPBG,TPD52,TPI1,TPST2,UGP2,VEGFA,VHL,VLDLR,WSB1,XPNPEP1,ZFP36,ZNF292
**HARRIS_HYPOXIA**
ADM,AK3,ALDOA,ANGPT2,APEX1,BHLHE40,BIK,BNIP3,BNIP3L,CA12,CA9,CCL2,CCNG2,CD99,CDKN1A,CDKN1B,COL5A1,CP,CXCL8,DDIT3,EDN1,EDN2,ENO1,ENPEP,EP

AS1,EPO,F3,FGF3,FLT1,FOS,FTL,GAPDH,HDAC9,HGF,HIF1A,HK1,HK2,HMOX1,IGF2,IGFBP1,IGFBP2,IGFBP3,IL6,JUN,L1CAM,LDHA,LRP8,MIF,MMP13,NFKB1,P4HA1,PDGFB,PFKL,PFKP,PGF,PGK1,PKM,PLAUR,PRPS1,PTGS2,RP1,SAT1,SLC2A1,SLC2A3,SPP1,STC1,TAGLN,TEK,TF,TFF3,TFRC,TGFA,TGFB1,TGFB3,TGM2,TH,TXN,VEGFA,VIM,XRCC5,XRCC6
**KIM_HYPOXIA**
ALDOA,ALDOC,AMPD3,ANGPTL4,BACH1,BNIP3L,ENO2,F3,GYS1,IGFBP3,JUN,LDHA,NKX3-2,PDLIM4,PFKP,PLIN2,SLC2A1,TPI1,VEGFA
**LEONARD_HYPOXIA**
ADORA2B,AK4,ALDOA,BHLHE40,BNIP3,CCNG2,CDKN1B,CDKN1C,DDIT3,DDIT4,DEPP1,DUSP1,EFNA1,F3,FOSL2,HNRNPA1,HOXA4,HOXA5,JMJD6,JUNB,KDM3A,MUC1,NFIL3,P4HA1,P4HA2,PDGFB,PFKFB4,PFKP,PGK1,PHC2,PLIN2,PPP1R3C,RASGRP1,SAP30,SERPINE1,SIK1,SLC16A3,SLC2A1,SLC2A3,SLC7A5,STC2,TGFA,VEGFA
**MENSE_HYPOXIA_UP**
ABCB6,ADM,ALDOC,ANG,ANGPTL4,ANKRD37,ASPH,ATF3,BHLHE40,BHLHE41,BNIP3,BNIP3L,C4orf3,CDK19,CEBPB,CEBPD,CLK3,CLPB,CTTN,DIDO1,DPCD,EEF1AKMT3,EGLN1,ELL2,ENO2,ERICH1,ERO1A,FAM110C,FAM13A,FAM162A,FOXD1,FUT11,GADD45A,GBE1,GNA13,GOSR2,GPI,GPRIN3,HCFC1R1,HILPDA,HK2,INSIG1,INSIG2,JMJD6,KDM3A,KDM4B,KDM4C,KIAA2013,KLF4,KLF7,KLHL24,LARP6,LCORL,LOX,MAFF,MED6,MIR210,MXI1,NADSYN1,NAMPT,NDRG1,NFIL3,NOL3,OSMR,P4HA1,P4HA2,PDK1,PEX13,PFKFB3,PFKFB4,PGK1,PLIN2,PLOD2,PPFIA4,PPP1R15A,PPP1R3C,PRPSAP1,RBPJ,RIOK3,RNASE4,RORA,SCD,SERGEF,SLC2A3,SOD2,SPAG4,STARD4,STC2,STK4,TC2N,THAP8,TIPARP,TMEM65,TSLP,VEGFA,WDR54,ZBTB25

**Supplemental table 6. TMA Corhort**											
**Time of operation**	**OS**	**Follow-up time**	**OS.time**	**Organizational code**	**Tumor surgery organ**	**Gender**	**Age**	**T**	**N**	**M**	**CLEC11A area per field %（40X）**
2006-07-25	0	2015.7	108	RDgCol0609A0277	colon	Male	62	T3	N0	M0	5.817
2006-07-31	0	2015.7	108	RDgCol0609A0280	colon	Female	81	T3	N0	M0	0
2006-07-31	1	2009/2/17	31	RDgCol0609A0281	colon	Male	70	T3	N0	M0	25.08
2006-08-05	0	2015.7	107	RDgCol0609A0284	colon	Male	71	T3	N0	M0	8.291
2006-08-11	1	2011/3/23	55	RDgCol0609A0285	colon	Female	70	T3	N1a	M0	31.867

2006-08-18	0	2015.7	107	RDgCol0609A0287	colon	Male	55	T3	N0	M0	0.158
2006-08-19	1	2009/2/12	30	RDgCol0609A0288	colon	Female	58	T3	N2a	M0	56.389
2006-08-26	1	2007/1/25	5	RDgCol0609A0289	colon	Male	72	T3	N0	M0	18.612
2006-09-04	0	2015.7	106	RDgCol0609A0291	colon	Female	62	T3	N0	M0	4.359
2006-09-06	0	2015.7	106	RDgCol0609A0292	colon	Male	85		N0	M0	0
2006-09-06	1	2011/11/27	62	RDgCol0609A0293	colon	Female	75		N1b	M0	27.702
2006-09-08	1	2010/12/5	51	RDgCol0609A0295	colon	Female	24	T3	N2a	M0	50.969
2006-09-11	1	2011/4/12	55	RDgCol0609A0296	colon	Male	80	T3	N0	M0	22.468
2006-09-11	1	2008/9/30	24	RDgCol0609A0297	colon	Male	75	T4a	N1b	M0	66.021
2006-09-14	0	2015.7	106	RDgCol0609A0298	colon	Female	59	T3	N0	M0	3.206
2006-09-21	1	2008/11/3	26	RDgCol0611A0309	colon	Male	83	T4b	N1b	M0	36.423
2006-09-28	1	2008/10/16	25	RDgCol0611A0311	colon	Female	52	T3	N2a	M0	48.586
2006-09-28	0	2015.7	106	RDgCol0611A0312	colon	Male	80	T3	N0	M0	0
2006-10-07	1	2011/6/29	56	RDgCol0611A0313	colon	Male	81	T3	N0	M0	18.235
2006-10-09	1	2009/8/24	34	RDgCol0611A0314	colon	Male	75	T3	N0	M0	0.702
2006-10-16	1	2007/11/24	13	RDgCol0611A0317	colon	Male	75	T3	N1b	M0	38.438
2006-10-19	0	2015.7	105	RDgCol0611A0318	colon	Male	成	T3	N0	M0	0
2006-10-21	1	2007/10/2	12	RDgCol0611A0319	colon	Female	90	T3	N0	M0	24.353
2006-10-29	0	2015.7	105	RDgCol0611A0322	colon	Male	56		N0	M0	0
2006-10-30	0	2015.7	105	RDgCol0611A0324	colon	Male	65	T3	N0	M0	23.73
2006-10-30	0	2015.7	105	RDgCol0611A0325	colon	Female	65		N1b	M0	21.447
2006-11-01	1	2007/7/24	8	RDgCol0611A0327	colon	Male	71	T3	N1b	M1b	26.315

2006-11-03	1	2009/3/8	28	RDgCol0612A0334	colon	Female	76	T4b	N1a	M0	41.085
2006-11-07	0	2015.7	104	RDgCol0612A0335	colon	Male	72	T4a	N0	M0	9.721
2006-11-07	1	2011/3/9	52	RDgCol0612A0336	colon	Male	76	T3	N0	M0	1.492
2006-11-08	1	2007/12/31	13	RDgCol0612A0337	colon		72	T3	N2a	M1b	34.179
2006-11-16	0	2015.7	104	RDgCol0612A0340	colon	Male	成	T3	N0	M0	0
2006.11	1	2013/6/5	79	RDgCol0612A0341	colon	Female	65	T4a	N0	M0	11.015
2006-11-21	0	2015.7	104	RDgCol0612A0343	colon	Male	79	T3	N0	M0	32.652
2006-11-23	1	2007/5/26	6	RDgCol0612A0344	colon	Female	61	T3	N0	M0	29.007
2006-11-27	1	2008/7/5	20	RDgCol0612A0345	colon	Male	75		N1a	M0	58.017
2006-11-29	1	2006/12/26	1	RDgCol0612A0346	colon	Female	74	T4b	N2a	M0	42.459
2006-12-01	1	2014/9/2	93	RDgCol0612A0348	colon	Female	79	T4a	N1a	M0	11.811
2006-12-06	1	2010/4/21	40	RDgCol0612A0352	colon	Male	50	T3	N0	M0	0.819
2006-12-06	1	2015/6/13	102	RDgCol0612A0353	colon	Female	82	T3	N1b	M0	19.902
2006-12-12	1	2014/3/6	87	RDgCol0612A0356	colon	Female	56	T4a	N0	M0	14.193
2006-12-13	0	2015.7	103	RDgCol0701A0395	colon	Female	83	T2	N0	M0	0
2006-12-18	0	2015.7	103	RDgCol0701A0398	colon	Male	72	T3	N0	M0	9.159
2006-12-22	1	2009/4/14	28	RDgCol0701A0400	colon	Female	成	T3	N0	M0	8.991
2006-12-27	1	2008/11/7	23	RDgCol0701A0402	colon	Male	60	T3	N0	M0	14.971
2006-12-29	1	2012/12/5	72	RDgCol0701A0403	colon	Male	78	T3	N1a	M0	50.763
2007-01-05	1	2007/7/13	6	RDgCol0701A0404	colon	Male	80	T4b	N0	M0	10.681
2007.1	1	2008/1/7	12	RDgCol0701A0405	colon	Female	62	T3	N2b	M0	0
2007.1	1	2011/3/9	50	RDgCol0704A0431	colon	Male	62	T3	N0	M0	0

2007-01-15	0	2015.7	102	RDgCol0704A0434	colon	Female	75	T2	N0	M0	11.707
2007-01-17	1	2008/5/14	16	RDgCol0704A0435	colon	Male	成	T3	N0	M0	44.872
2007-01-24	0	2015.7	102	RDgCol0704A0436	colon	Female	成	T3	N0	M0	17.982
2007-01-21	1	2008/7/10	18	RDgCol0704A0437	colon	Female	71	T2	N0	M0	37.739
2007.1	0	2015.7	102	RDgCol0704A0438	colon	Female	70	T3	N1b	M0	28.158
2007.2	0	2015.7	101	RDgCol0704A0439	colon	Male	63	T3	N0	M0	0
2007.2	1	2008/10/6	20	RDgCol0704A0440	colon	Male	74		N1a	M0	38.546
2007.2	1	2008/6/26	16	RDgCol0704A0441	colon	Female	79	T4b	N1b	M0	42.318
2007-02-14	1	2007/7/15	5	RDgCol0704A0476	colon	Male	48	T4b	N0	M0	39.009
2007.2	1	2012/12/17	70	RDgCol0704A0442	colon	Male	89	T3	N0	M0	22.476
2007-02-18	1	2010/2/14	36	RDgCol0704A0475	colon	Male	90	T3	N0	M0	31.622
2007-02-26	1	2008/11/5	21	RDgCol0704A0444	colon	Female	62		N2b	M0	44.491
2007.2	1	2011/6/26	52	RDgCol0704A0447	colon	Female	80	T3	N0	M0	44.377
2007.3	1	2008/1/22	10	RDgCol0704A0452	colon	Female	67	T4a	N1a	M0	46.965
2007.3	1	2011/4/9	49	RDgCol0704A0455	colon	Female	78	T3	N1a	M0	55.305
2007-03-20	0	2015.7	100	RDgCol0704A0458	colon	Female	57	T2	N0	M0	21.912
2007.3	0	2015.7	100	RDgCol0704A0459	colon	Male	65	T3	N0	M0	9.608
2007-03-22	1	2012/5/23	62	RDgCol0704A0460	colon	Male	80	T3	N0	M0	2.874
2007-03-28	1	2010/11/2	44	RDgCol0704A0463	colon	Male	67	T3	N1b	M0	25.675
2007.3	1	2010/4/22	37	RDgCol0704A0465	colon	Male	72	T3	N1a	M0	20.74
2007-03-31	0	2015.7	100	RDgCol0704A0466	colon	Male	62	T3	N0	M0	0
2007.3	1	2014/2/21	83	RDgCol0705A0524	colon	Female	82	T2	N0	M0	48.54

2007-04-04	0	2015.7	99	RDgCol0705A0525	colon	Female	57	T3	N1a	M0	27.332
2007.4	0	2015.7	99	RDgCol0705A0528	colon	Male	78	T1	N0	M0	19.36
2007.4	0	2015.7	99	RDgCol0705A0529	colon	Female	77	T3	N0	M0	25.372
2007.4	0	2015.7	99	RDgCol0705A0530	colon	Female	84	T2	N0	M0	29.591
2007.4	0	2015.7	99	RDgCol0705A0531	colon	Male	80	T3	N1b	M0	22.346
2007.4	0	2015.7	99	RDgCol0705A0532	colon	Female	65	T3	N0	M0	52.123
2007.4	1	2010/2/5	34	RDgCol0705A0533	colon	Female	76	T3	N0	M0	60.916
2007.4	1	2014/2/9	82	RDgCol0705A0535	colon	Male	74	T3	N0	M0	26.76
2007.4	0	2015.7	99	RDgCol0705A0537	colon	Female	65	T3	N2a	M0	38.098
2007-04-27	1	2008/7/25	15	RDgCol0705A0539	colon	Female	71	T3	N2a	M0	47.009
2007-04-27	0	2015.7	99	RDgCol0705A0540	colon	Female	54	T4a	N1a	M0	44.084
2007.4	1	2009/4/19	24	RDgCol0705A0541	colon	Female	67	T3	N1b	M0	64.632
2007.4	0	2015.7	99	RDgCol0705A0544	colon	Male	58	T3	N0	M0	18.813
2007.4	1	2013/12/14	80	RDgCol0706A0575	colon	Male	62	T2	N0	M0	17.645
2007.5	0	2015.7	98	RDgCol0706A0576	colon	Female	54	T3	N0	M0	3.358
2007.5	1	2008/4/29	11	RDgCol0706A0577	colon	Male	76	T3	N0	M0	37.332
2007.5	1	2007/11/29	6	RDgCol0706A0579	colon	Female	73	T3	N0	M0	33.288
2007.5	0	2015.7	98	RDgCol0706A0580	colon	Male	53	T3	N0	M0	4.95
2007.5	1	2009/9/3	28	RDgCol0706A0583	colon	Male	64	T3	N0	M0	9.539
