# Supplementary material for: Cancer-associated fibroblasts enhance colorectal cancer lymphatic metastasis via CLEC11A/LGR5-mediated WNT pathway activation
Source: J Clin Invest. 2025 Oct 15;135(20):e194243. doi: 10.1172/JCI194243 (PMC12520694; doi:10.1172/JCI194243)

Full unedited blot for Figure 3L

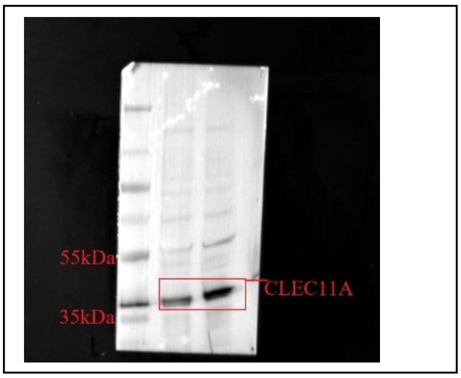

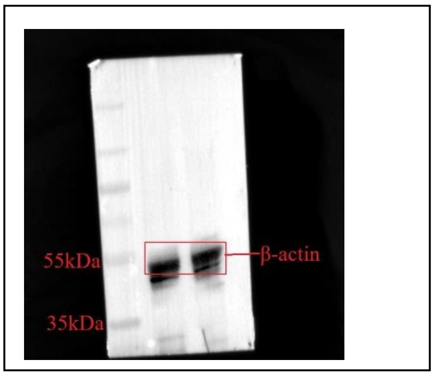

Full unedited blot for Figure 4R

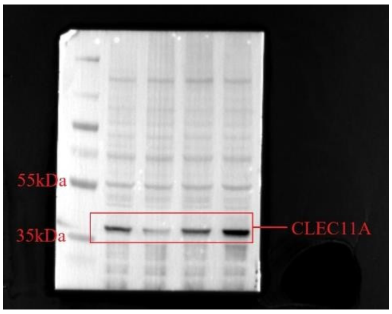

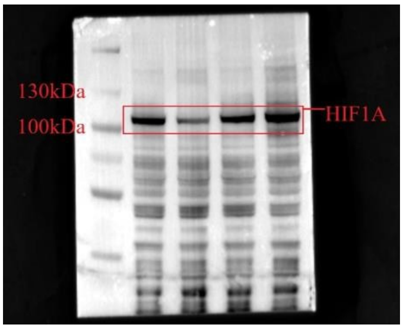

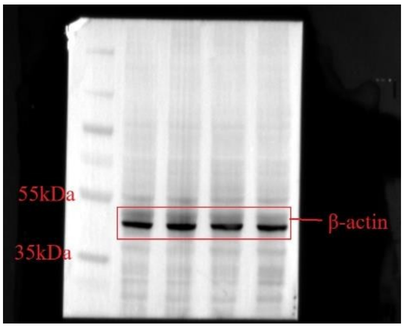

Full unedited blot for Figure 6D:

SW480:

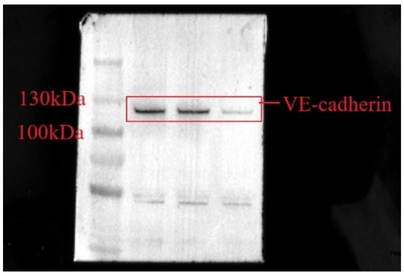

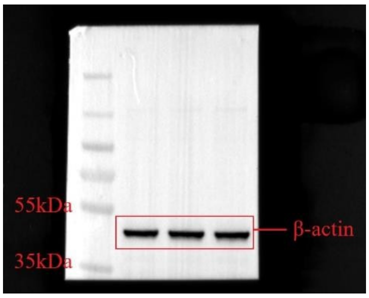

HCT116:

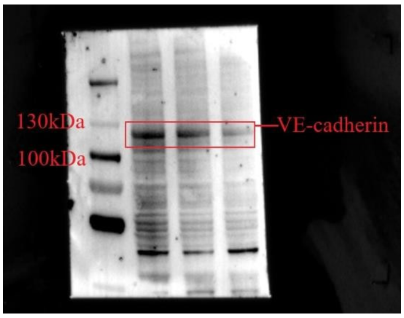

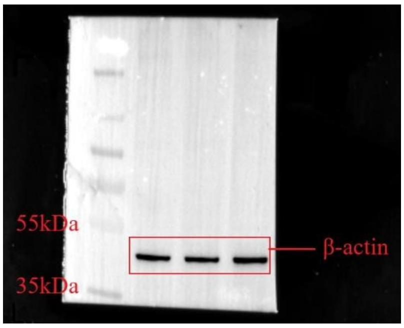

Full unedited blot for Figure 7D

HCT116:

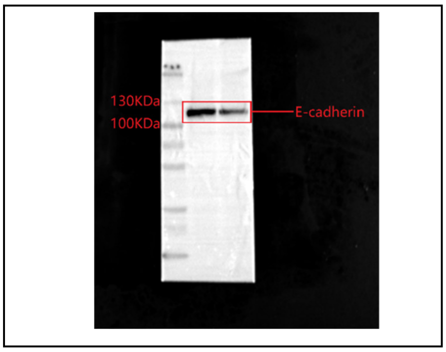

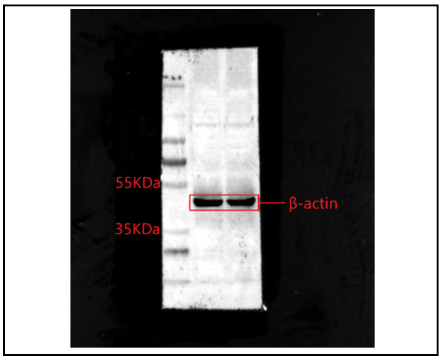

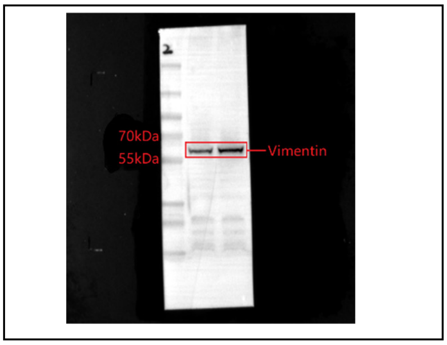

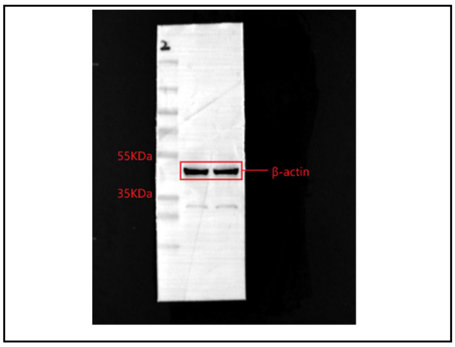

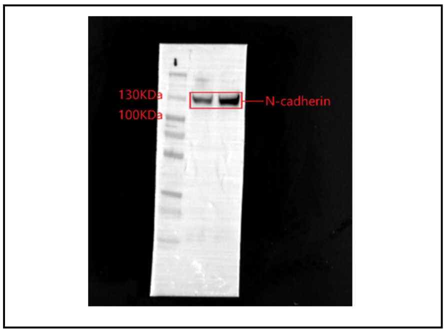

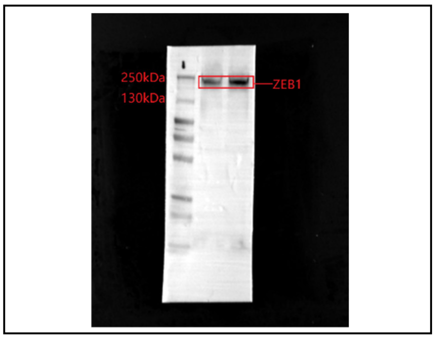

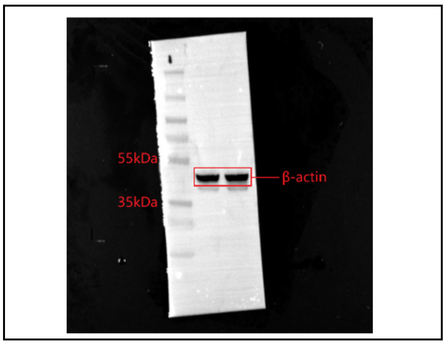

Full unedited blot for Figure 7D

SW480:

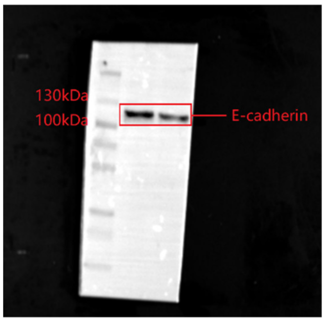

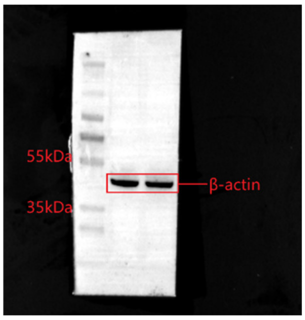

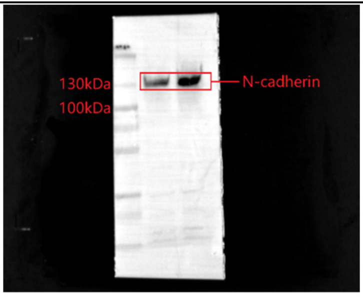

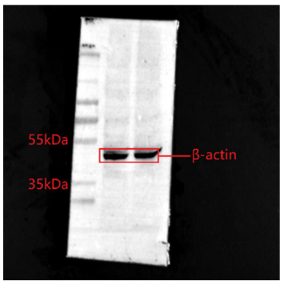

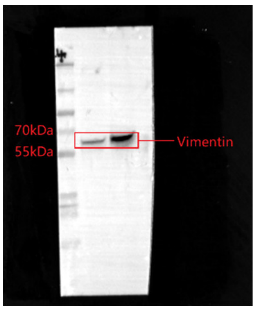

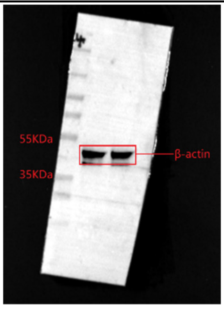

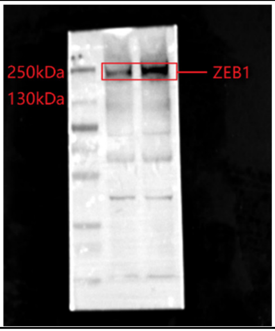

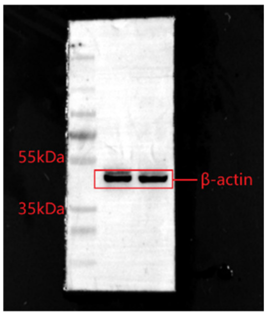

Full unedited blot for Figure 7G

SW480:

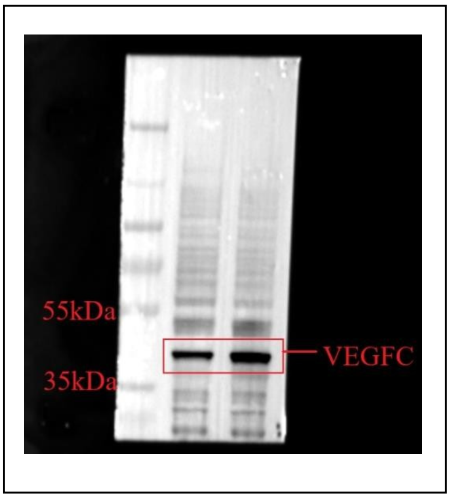

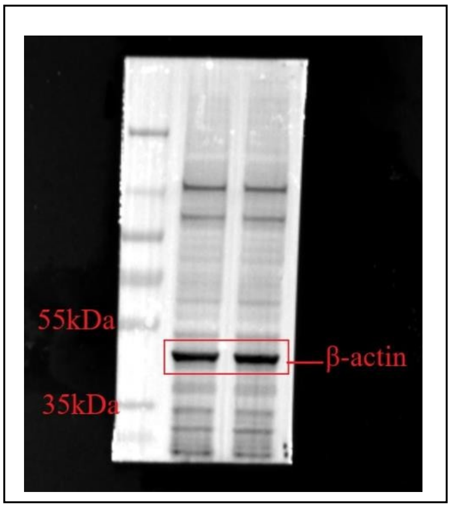

HCT116:

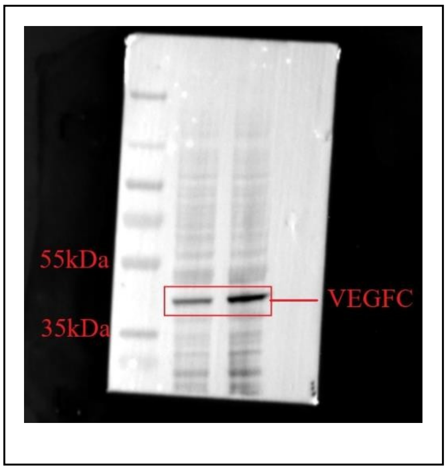

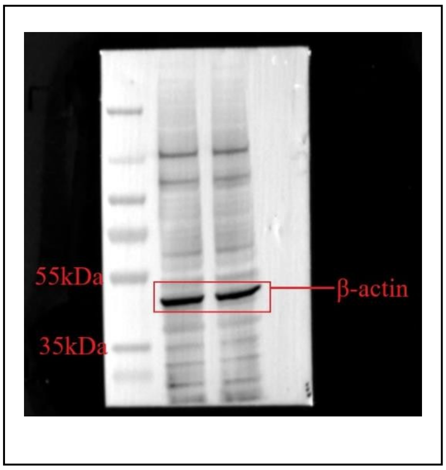

Full unedited blot for Figure 8G:

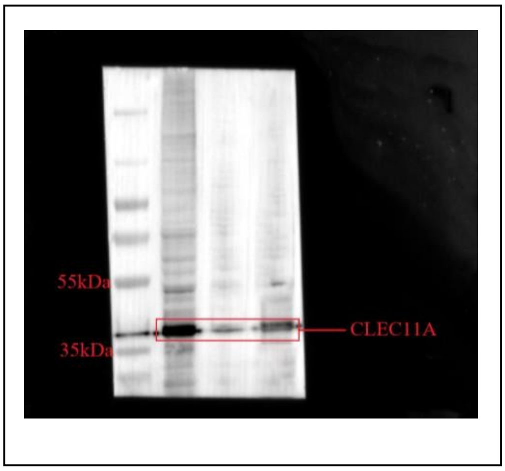

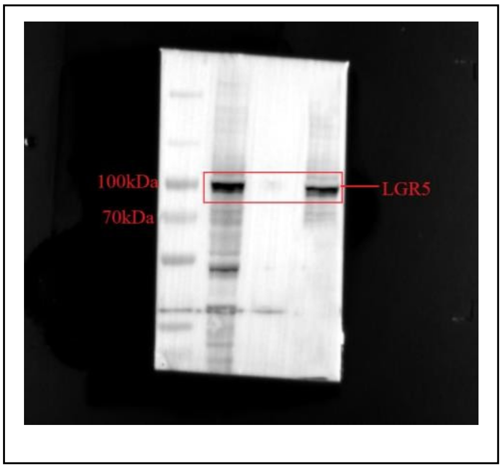

Full unedited blot for Figure 9F:

HCT116:

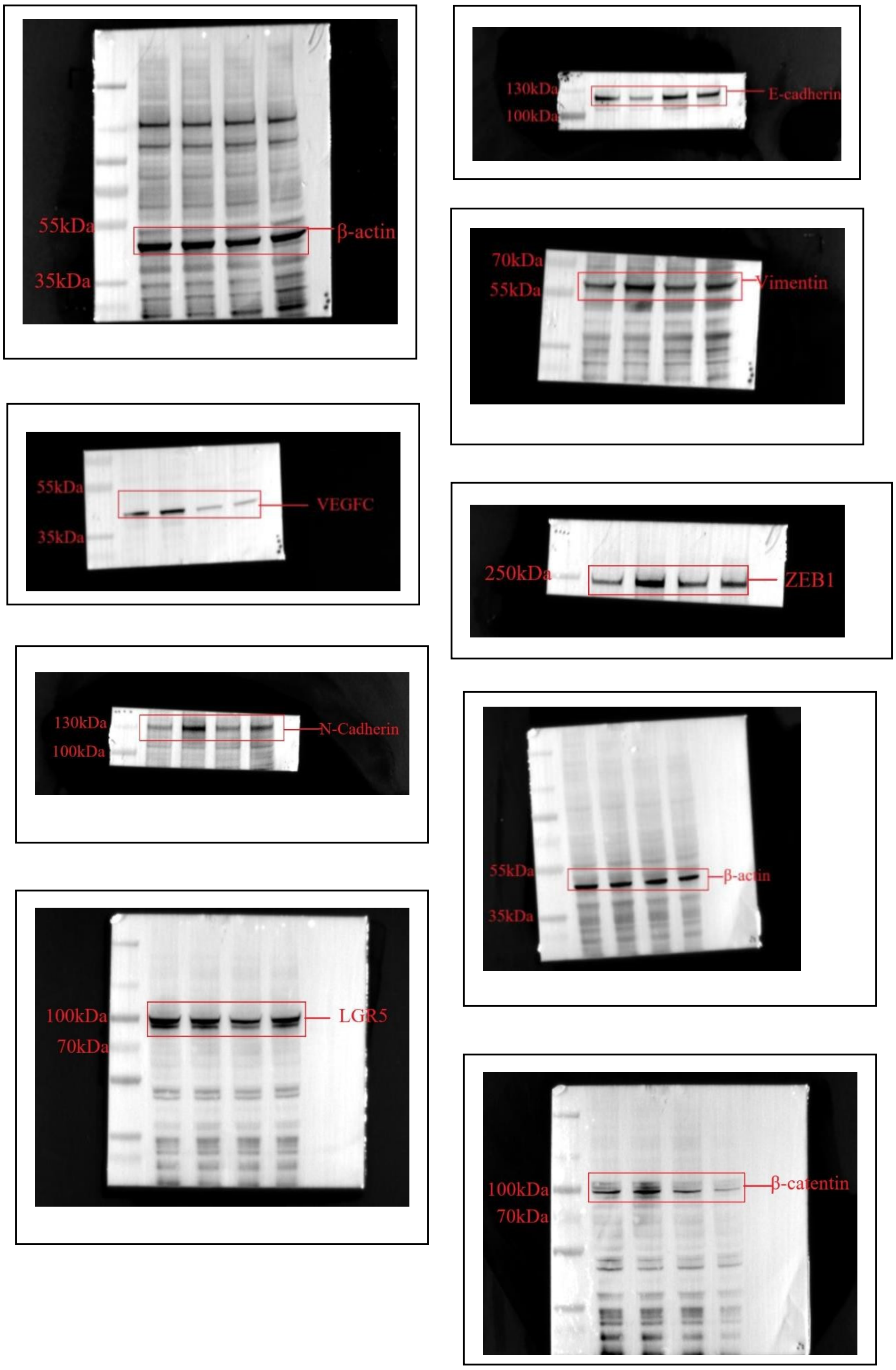

Full unedited blot for Figure 9F:

SW480:

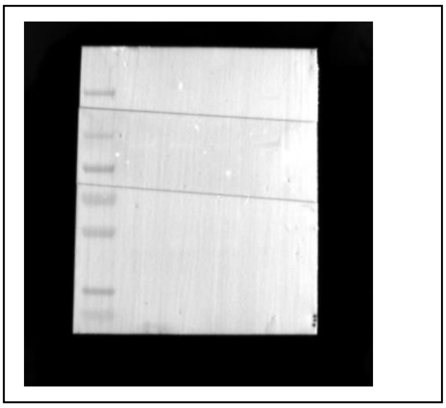

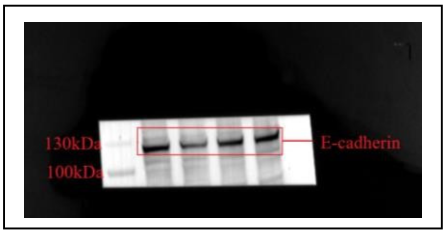

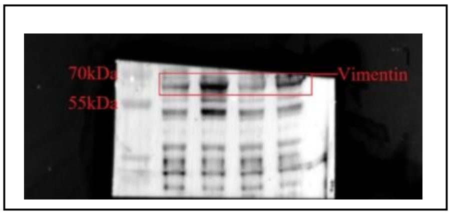

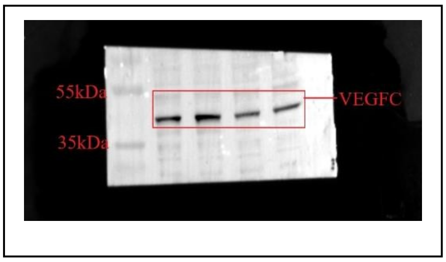

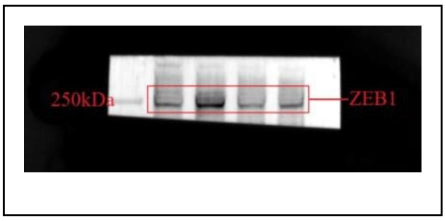

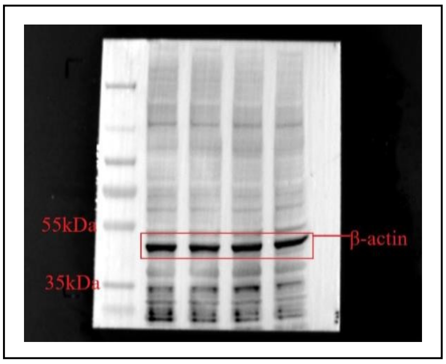

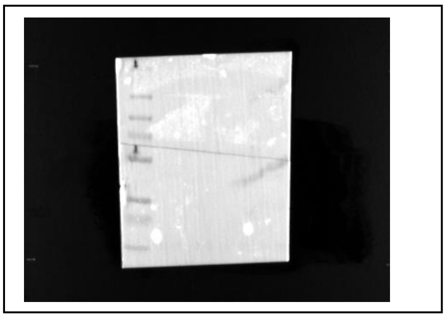

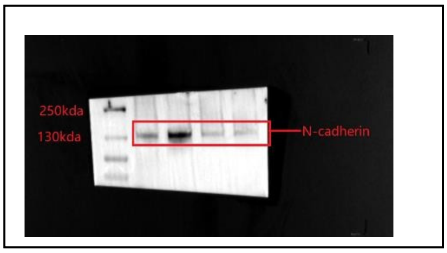

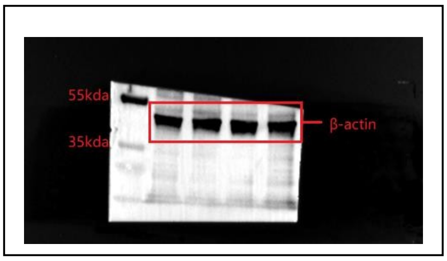

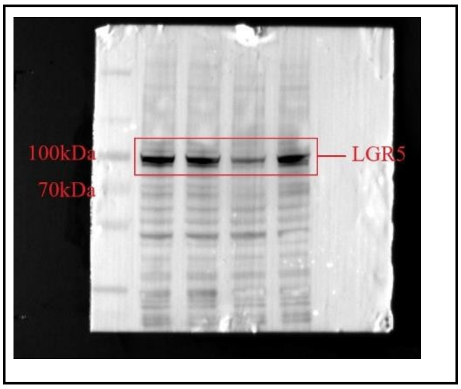

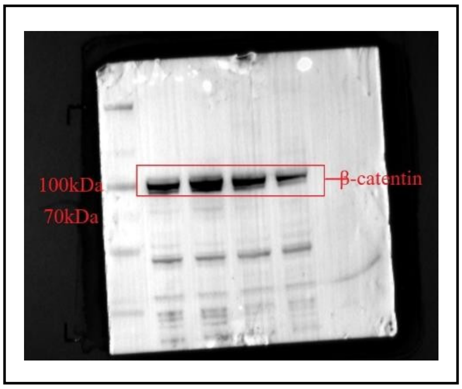

Full unedited blot for Supplemental Figure 7 B:

SW480：

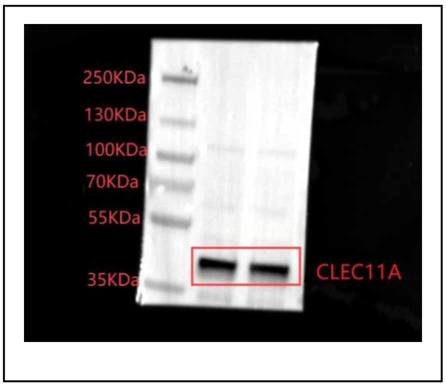

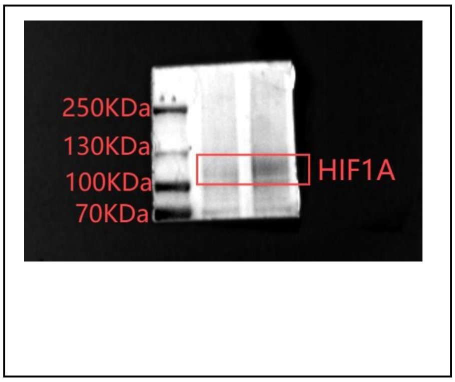

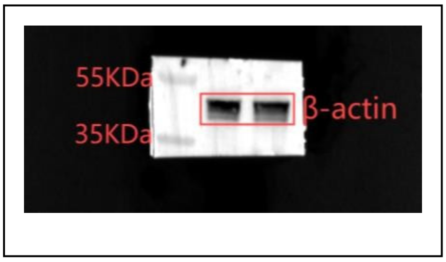

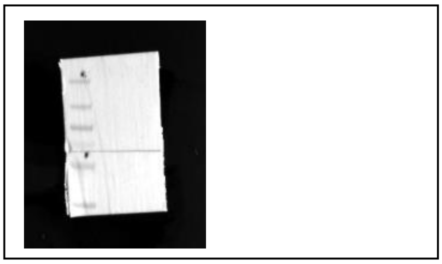

T Cell:

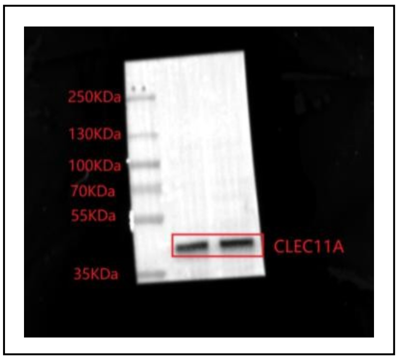

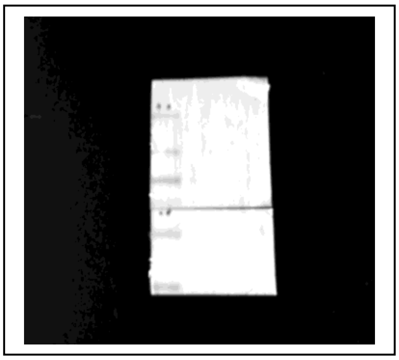

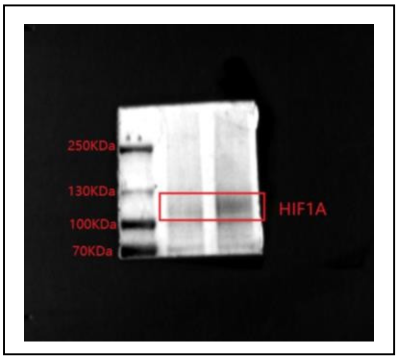

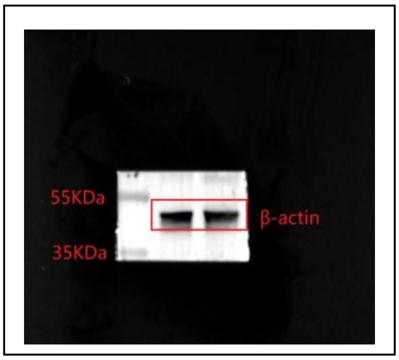

Full unedited blot for Supplemental Figure 7 B:

HUVEC:

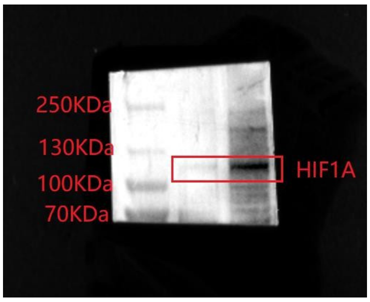

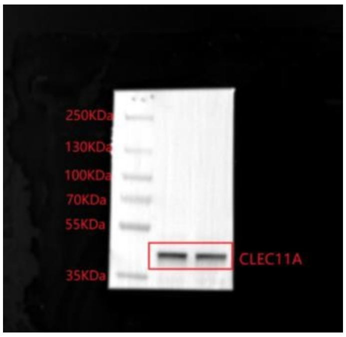

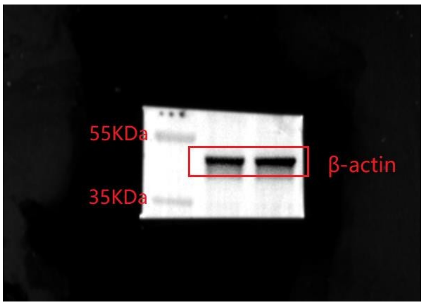

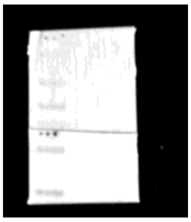

Pericyte:

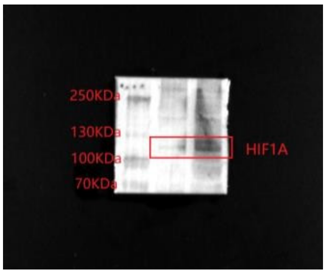

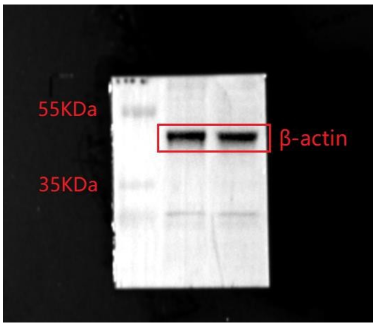

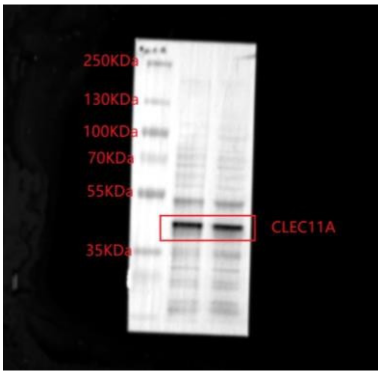

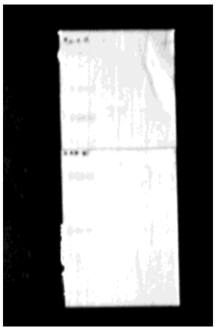

Full unedited blot for Supplemental Figure 7 B:

CAF:

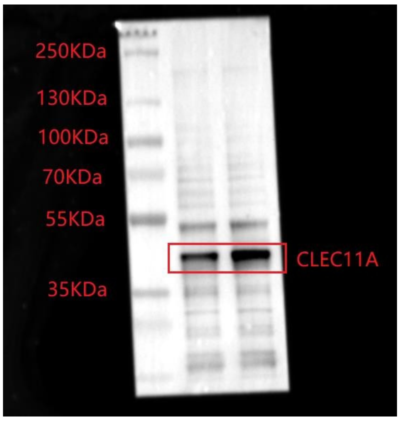

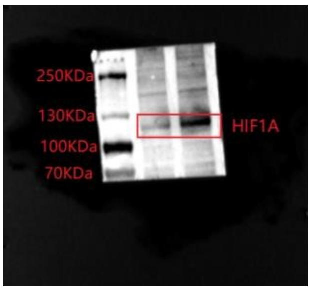

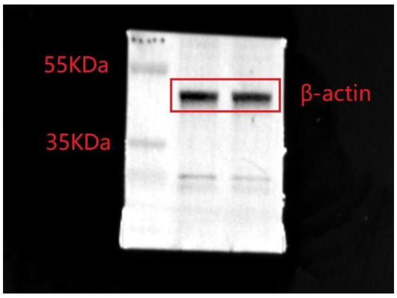

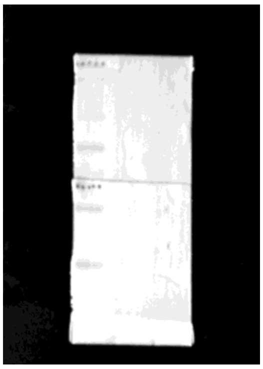

Full unedited blot for Supplemental Figure 16

CAF-OE-CLEC11A:

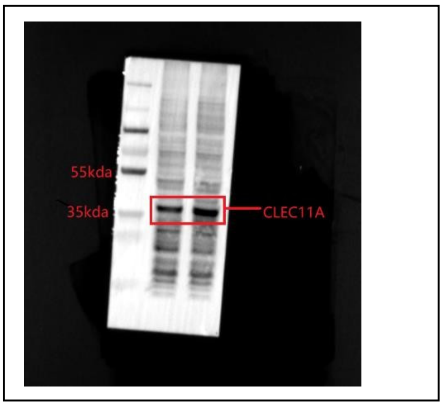

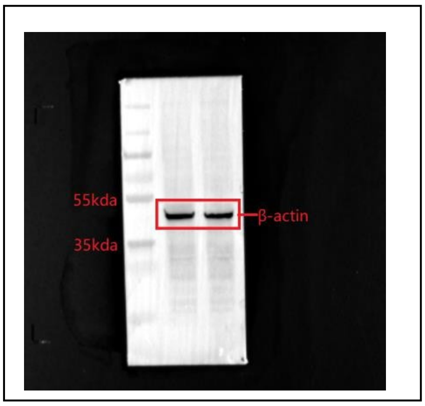

CAF-sh-CLEC11A:

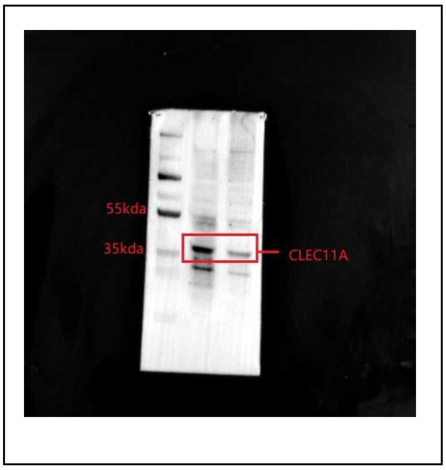

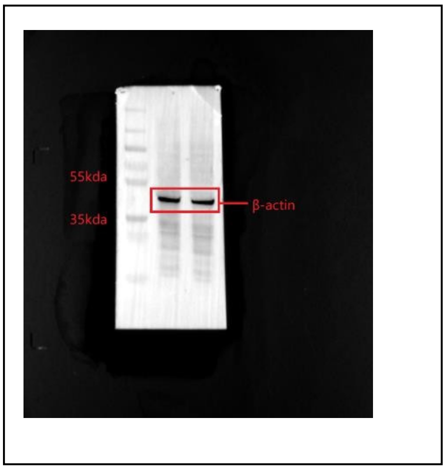

Full unedited blot for Ref Figure 7

HCT116 (Control CAF-NC CAF-CLEC11A):

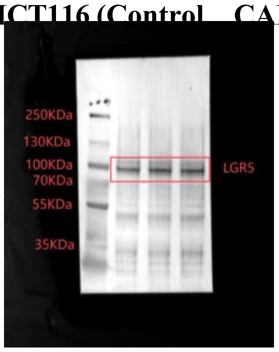

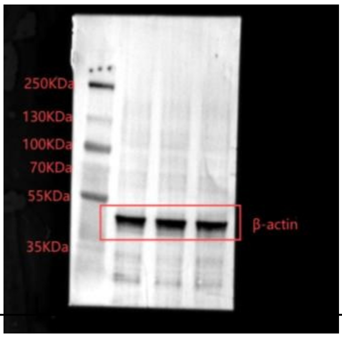

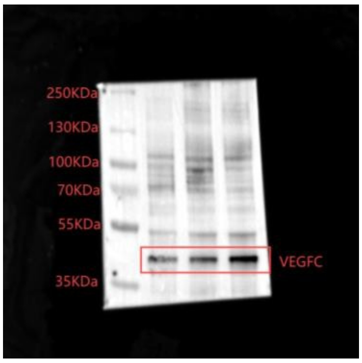

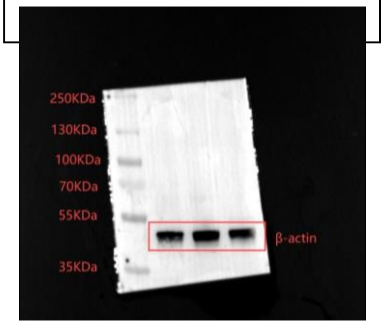

HCT116(Control rhCLEC11A):

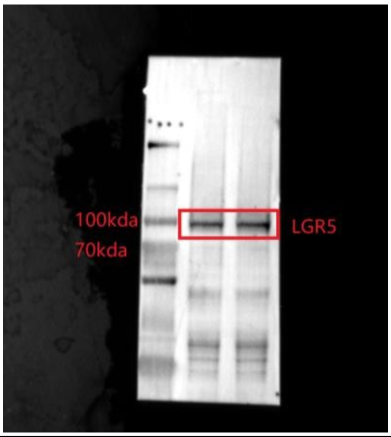

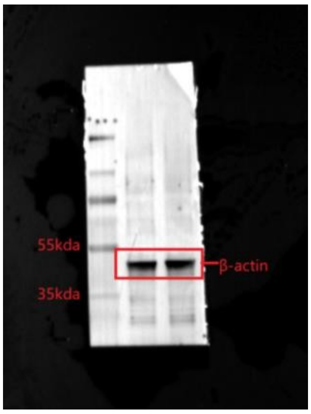

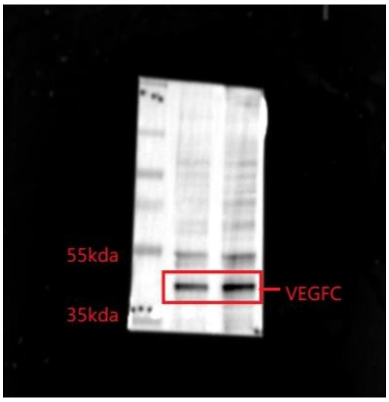

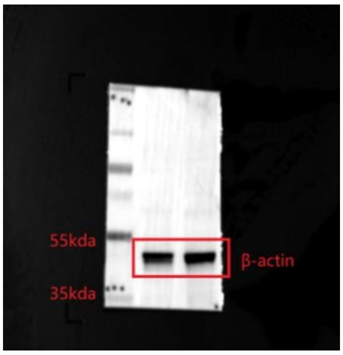

Supplement: Unedited blot and gel images [file jci-135-194243-s344.pdf]
